# Suzuki coupling-based synthesis of VATPase inhibitor archazolid natural product derived fragments†

**DOI:** 10.1039/c9ra07050h

**Published:** 2019-10-10

**Authors:** Cooper T. Vincent, Evan T. Long, Holly C. Jones, Jeffrey C. Young, P. Clint Spiegel, Gregory W. O'Neil

**Affiliations:** Department of Chemistry, Western Washington University Bellingham WA USA 98229 oneilg@wwu.edu; Department of Biology, Western Washington University Bellingham WA USA 98229

## Abstract

An archazolid natural product fragment that displays dose-dependent inhibition of the vacuolar-type ATPase (VATPase) has been synthesized by a high-yielding Suzuki coupling of two complex subunits. Similarly, a further simplified fragment was prepared and evaluated for VATPase inhibitory activity. This compound did inhibit the VATPase, as evidenced by growth inhibition of etiolated *Arabidopsis* seedlings, however at approximately 10× lower potency than the more complex fragment. Cyclooxygenase (COX) enzyme inhibition was not observed for either fragment.

## Abstract 2

An archazolid natural product fragment that displays dose-dependent inhibition of the vacuolar-type ATPase (VATPase) has been synthesized by a high-yielding Suzuki coupling of two complex subunits.

## Graphical abstract

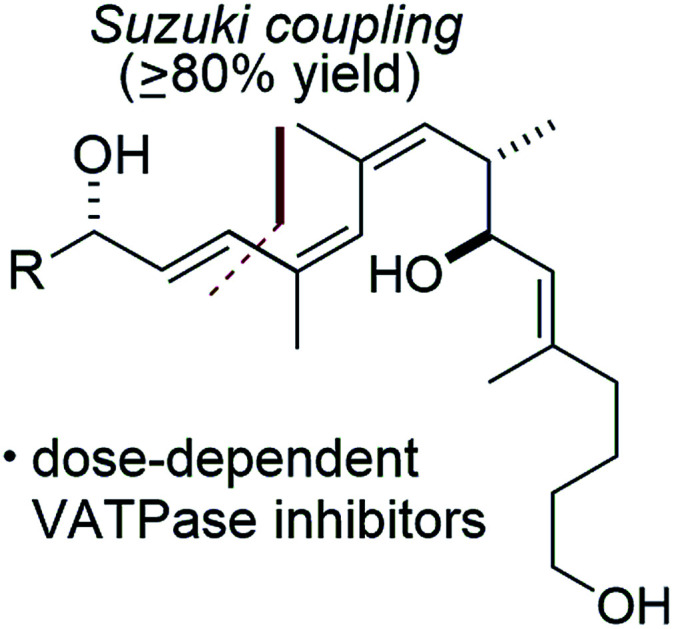

## Introduction

**Scheme 1:**
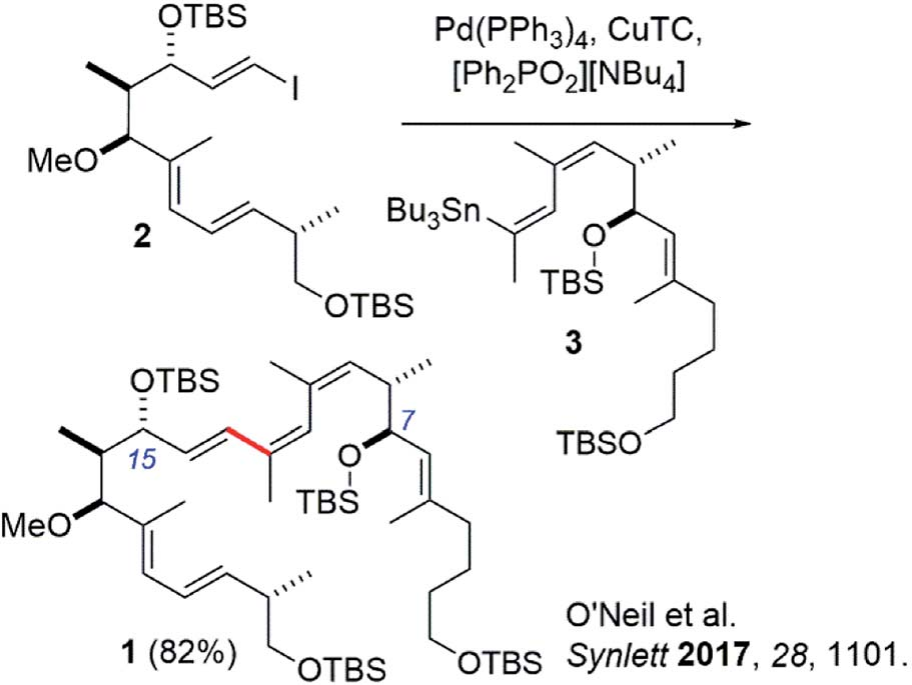
Previous synthesis of archazolid-based VATPase inhibitor compound 1 by Stille coupling.

Recently our group reported the synthesis of an archazolid natural product fragment (1) that, after removal of the TBS protecting groups, displayed dose-dependent inhibition of the vacuolar-type ATPase (VATPase).^1^ The VATPase has continued to emerge as a promising therapeutic target associated with several severe forms of cancer, as evidenced by a number of recent studies involving the archazolids.^2–10^ Key to our synthesis of 1 was a high-yielding Stille coupling between iodide 2 and stannane 3 to complete the conjugated *Z*,*Z*,*E*-triene (Scheme 1).^11^ This particular triene is unique to the archazolids and happens to link two important pharmacophoric elements, the C7- and C15 hydroxyl groups.^12^

While promising, others have commented on the drawbacks of Stille couplings due to the toxicity associated with organotin compounds.^13,14^ In line with these recommendations, we set out to investigate a tin-free synthesis of our VATPase inhibitor lead compound. Herein we report a similarly effective Suzuki coupling strategy to synthesize compound 1, in which tin has been replaced by a boronic acid derivative. We argue that this new approach represents to date the most direct, convergent, and a greener method for synthesizing the important triene region of this exciting class of compounds.

## Results and discussion

**Scheme 2:**
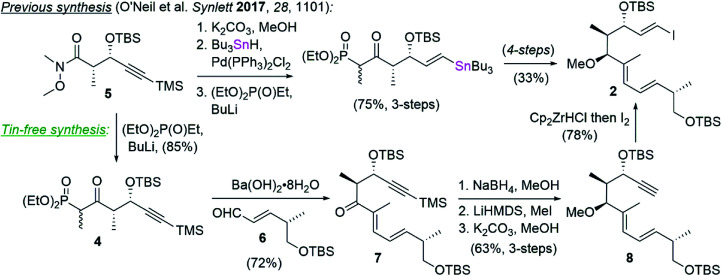
An alternative tin-free synthesis of vinyl iodide 2.

Tin featured not only in our Stille coupling, but also in our synthesis of vinyl iodide 2,^1^ which was prepared by palladium catalyzed hydrostannylation followed by iododestannylation (Scheme 2). To circumvent the use of tin in this instance, we opted instead to carry the TMS-protected alkynyl phosphonate 4, prepared in a single step from known Weinreb amide 5,^15^ into a Horner–Wadsworth–Emmons (HWE) olefination^16^ with aldehyde 6.^17^ This reaction delivered ketone 7 in 72% yield and >10 : 1 *E* : *Z* selectivity by NMR analysis. The ketone in 7 was diastereoselectively reduced with NaBH_4_ and the resulting hydroxyl converted to the corresponding methyl ether by deprotonation with LiHMDS and alkylation with methyl iodide. Removal of the TMS group (K_2_CO_3_/MeOH) revealed the terminal alkyne in 8 and set the stage for vinyl iodide installation by treatment with Schwartz's reagent and trapping with iodine.^18^ In this way, vinyl iodide coupling partner 2 was completed in comparable yields and one fewer step from compound 4 than had previously been described (*i.e.* 6-steps and 36% overall yield *vs.* 7-steps and 25% yield) while also avoiding the use of tin.

**Scheme 3:**
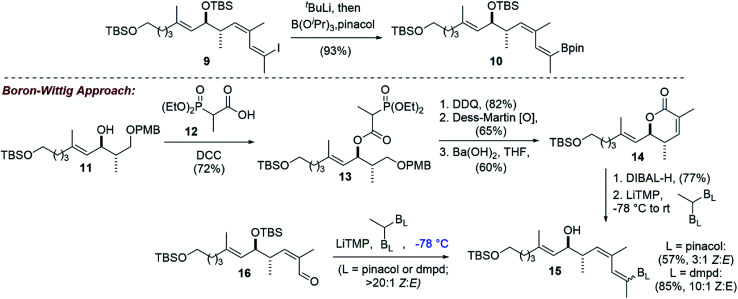
Vinyl boronic ester synthesis by lithium–halogen exchange and an alternative boron-Wittig approach that gave primarily the undesired *Z*-vinyl boronate.

Adapting our vinyl stannane synthesis to incorporate a vinyl boronic ester was possible from advanced vinyl iodide 9 by trapping with B(^i^PrO)_3_ rather than Bu_3_SnCl after lithium–halogen exchange (Scheme 3).^19^ Treatment with pinacol then allowed for isolation of vinyl pinacol boronate 10 in 93% yield. In an attempt to reduce the step count of our vinyl boronic ester synthesis, we became interested in evaluating a potential boron-Wittig reaction. This chemistry was pioneered by Endo and coworkers^20^ and more recently has been expanded upon by the Morken group.^21,22^ To that end, intermediate 11 was acylated with phosphonate acid 12 ^23^ using DCC, giving ester 13 in 72% yield. Removal of the PMB protecting group and oxidation of the resulting primary hydroxyl then allowed for an intramolecular HWE reaction. It was found that Ba(OH)_2_ gave the best overall yields of lactone 14, with some other reagents (*e.g.* KHMDS, DBU/LiCl) leading primarily to elimination. Reduction of 14 with DIBAL-H produced the corresponding lactol, that we thought could be converted directly to the desired *E*-vinyl boronic ester 15 by treatment with the lithium anion of a geminal bis(boronate). However, using Morken's procedure^21^ for the conversion of aldehydes to tri-substituted vinyl pinacol boronates (LiTMP, −78 °C) using 1,1-bis(pinacolboronato)ethane, no reaction was observed.

**Fig. 1:**
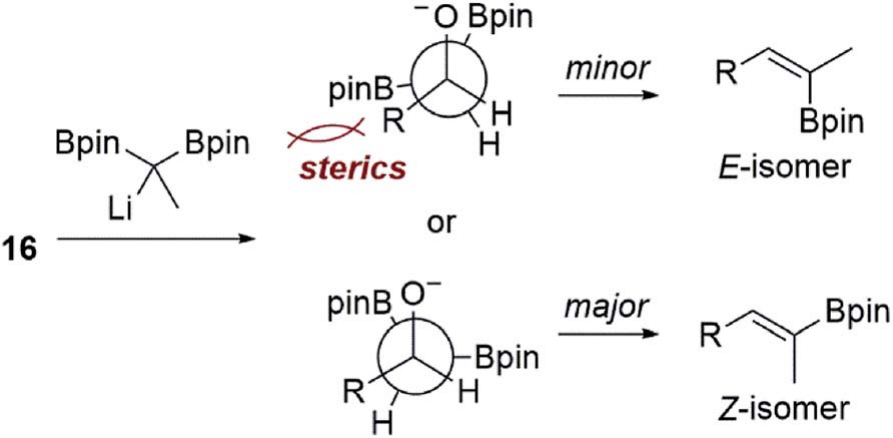
Possible stereochemical rationale for the *E*:*Z* selectivity observed in our boron-Wittig reactions with the lactol derived from 13 or alkdehyde 15 shown in Scheme 3.

We reasoned that the lactol was less reactive than an aldehyde in these reactions, and therefore repeated the experiment allowing the reaction to slowly warm to room temperature. Under these conditions, the vinyl boronate ester product 15 was indeed obtained, albeit as a 3 : 1 mixture of geometrical isomers by NMR analysis. Using 2D-NMR techniques (*e.g.* COSY and NOESY), it was determined that the major product from this reaction was the undesired *Z*-vinyl boronate. Morken's group had reported that in some cases switching to the dimethylpentanediolato (dpmd) diboronate led to a reversal of stereoselectivity when compared to the pinacol diboronate.^21^ Unfortunately, the reaction with 1,1-bis(dimethylpentanediolato)ethane was even more selective for the undesired *Z*-vinyl boronic ester (10 : 1 *Z* : *E*). When using aldehyde 16, reactions that proceeded at the colder temperatures (−78 °C) prescribed by Endo and Morken,^20,21^ both diboronates gave exclusively the undesired *Z*-vinyl boronic ester products. We can perhaps rationalize the stereochemical outcome of these reactions by considering a steric clash between the R group from the substrate and one of the boronic esters after addition of the lithiated diboronate and upon rotation into a conformation with the O- and B-groups *syn*-coplanar that is required for elimination (Fig. 1).^20,22^

**Scheme 4:**
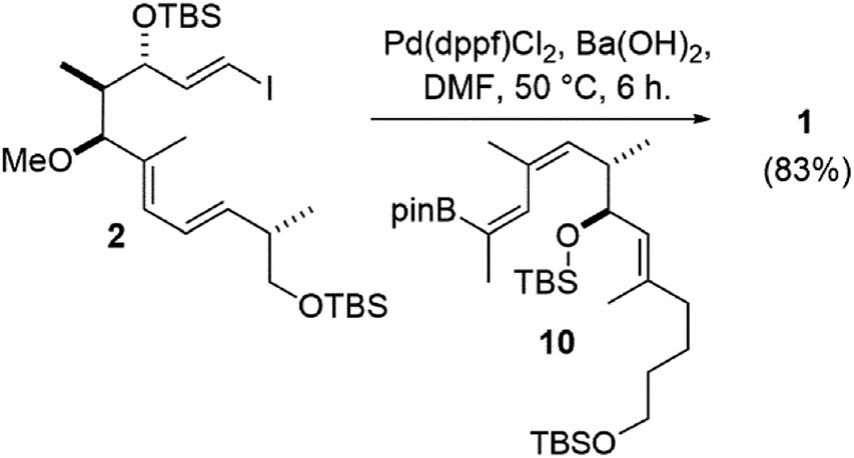
Synthesis of the archazolid conjugated triene by Suzuki coupling.

Nonetheless, the *E*/*Z* isomers from the boron-Wittig reactions were separable by chromatography on silica. Combined with our lithium–halogen exchange approach, sufficient quantities of the desired *Z*,*E*-boronic esters 10 and 15 were obtained to then investigate a Suzuki coupling with vinyl iodide 2.^1^ Gratifyingly, it was found that the use of 10 mol% Pd(dppf)Cl_2_ and Ba(OH)_2_ as base^24^ in DMF at 50 °C for 6 h gave the coupled product 1 in comparable yield to what was previously obtained from a Stille coupling^1^ (83% for the Suzuki *vs.* 82% from Stille coupling, Scheme 4).

**Scheme 5:**
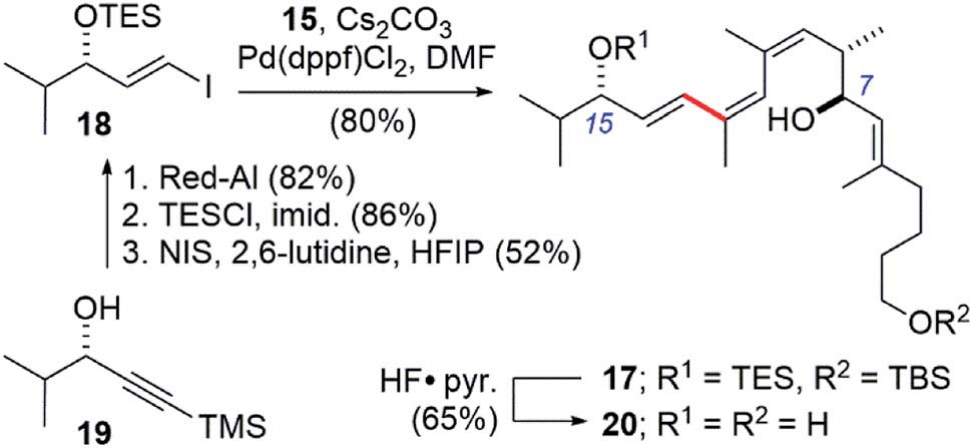
Synthesis of a simplified archazolid fragment 20 containing the pharmacophoric C7- and C15-hydroxyls linked by the *Z*,*Z*,*E*-triene.

We also took this opportunity to prepare a further simplified archazolid fragment 17 (Scheme 5). The requisite vinyl iodide 18 was prepared from alkynol 19, available in by enantioselective Noyori reduction^25^ of the corresponding alkynone.^26^ Red-Al reduction of the alkyne in 19 to the *trans*-alkene, TES protection of the alcohol, and iododesilylation of the TMS group gave 18 in 37% yield for the three steps. The Suzuki coupling with boronic ester 10 again proceeded efficiently, in this case using Pd(dppf)Cl_2_ and Cs_2_CO_3_ as base in DMF at room temperature,^27^ giving the coupled product 17 in 80% yield.

**Fig. 2:**
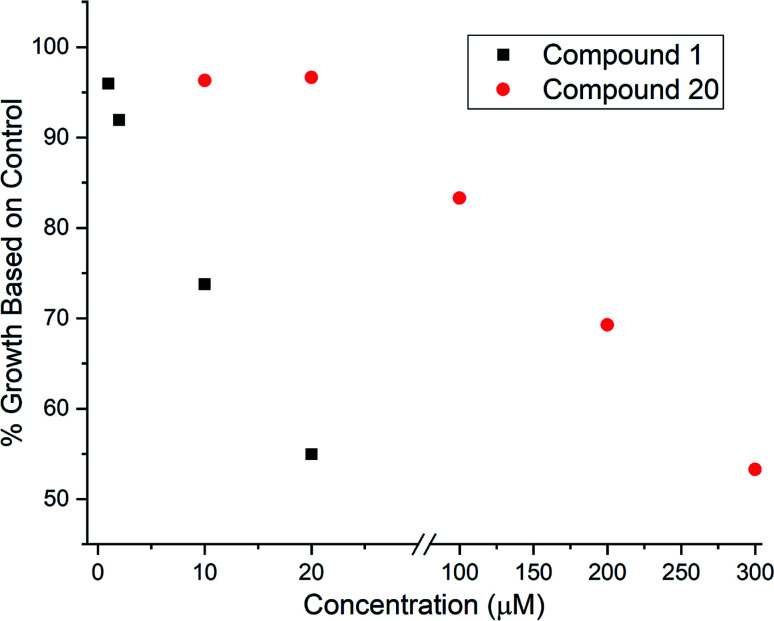
VATPase assay results for compound 1 (after TBS removal) and compound 20 where % growth relative to control represents the average hypocotyl length for 40–60 *Arabidopsis* seedlings at the concentrations indicated relative to the average hypocotyl length of *Arabidopsis* seedlings grown in the absence of any inhibitors.

Given that 17 contained an intact C7–C15 region of the archazolids, we suspected this compound might exhibit VATPase inhibitory activity and opted to assay the desilylated compound 20 using our *Arabidopsis*-based method.^28,29^ As shown in Fig. 2, while 20 did exhibit dose dependent growth inhibition of etiolated *Arabidopsis* as an indicator for impaired VATPase function, its activity was significantly lower (∼10× less potent) than compound 1. This data suggests that while the C7–C15 segment of the archazolids is important for VATPase binding, additional functionality present in compound 1 also contributes to its effectiveness as a VATPase inhibitor.

**Fig. 3:**
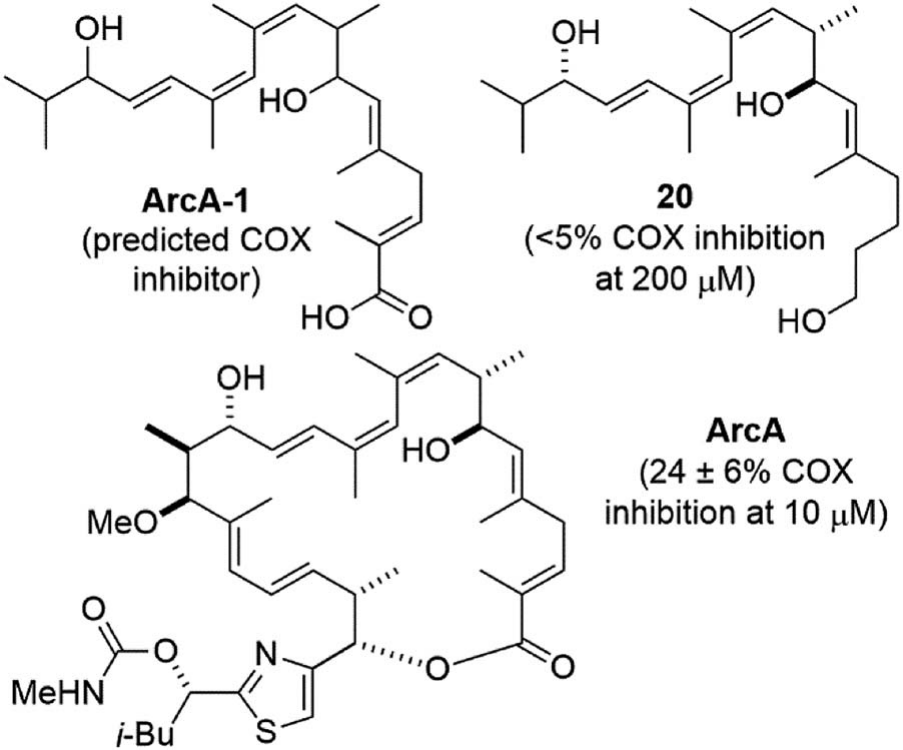
Structures and cyclooxygenase (COX) inhibitory activity of proposed COX inhibitor ArcA-1,^30^ archazolid A (ArcA), and compound 20.

Compound 20 is similar the predicted cyclooxygenase (COX) enzyme inhibitor ArcA-1 ^30^ (Fig. 3). When tested using a commercial COX screening assay for inhibition of COX-1 and COX-2,^31^ no measurable inhibition was observed at concentrations up to 200 μM. This was also true for compound 1, which we previously postulated could be due to its large size and/or lack of a carboxylic acid terminus.^1^ The latter might also apply to compound 20, however neither rationale would explain the modest reported COX inhibitory activity of archazolid A^30^ which is both sterically large and lacks a carboxylic acid.

## Conclusions

We have devised and executed a high-yielding Suzuki coupling-based synthesis of the challenging yet biologically important conjugated triene region of the archazolid natural products. The vinyl iodide synthesis reported here represents an improvement over our previous approach in terms of step count, yield, and avoidance of hazardous tin compounds. Two different vinyl boronic ester syntheses were investigated. Ultimately it was found that while a boron-Wittig reaction did give some of the desired compound, the majority of product obtained was the undesired geometrical isomer. The isomers were, however, separable and combined with an alternative iodo-Wittig followed by lithium–halogen exchange-based synthesis, sufficient amounts of the correct boronic ester isomer were obtained to then conduct Suzuki couplings. In this way, an archazolid natural product fragment 1 that had previously exhibited dose dependent VATPase inhibition along with a further simplified fragment 20 were synthesized. Compound 20 also showed dose dependent VATPase inhibition in our *Arabidopsis* VATPase assay, however with approximately 10× less potency than compound 1. The assay results support the importance of the C7–C15 region of the archazolids for VATPase inhibitory activity, but suggest additional functionality is needed for efficient VATPase inhibition. Compound 20 was also assayed for COX inhibitory activity based on its similarity to a predicted COX inhibitor. However, like compound 1, in our assay no measurable COX inhibition was observed for compound 20. Further studies are underway to better understand the interactions of compounds 1 and 20 with VATPase and COX enzymes toward the design of more efficient inhibitors with therapeutic potential.

## Experimental section

### General information

All reactions were carried out under N_2_ in flame-dried glassware. IR: Nicolet iS10 spectrometer, wavenumbers (*ν̃*) in cm^−1^. The solvents used were dried by passing the solvent through a column of activated alumina under nitrogen immediately prior to use. All reagents were purchased and used as received unless otherwise mentioned. All TLC analysis used 0.25 mm silica layer fluorescence UV_254_ plates. Flash chromatography: SilaCycle silica gel P60 (230–400 mesh). NMR: spectra were recorded on a Varian Mercury 300, or Inova 500 spectrometer in the solvents indicated; chemical shifts (*δ*) are given in ppm, coupling constants (*J*) in Hz. The solvent signals were used as references (C_6_D_6_: *δ*_C_ ≡ 128.0 ppm; residual C_6_H_6_ in C_6_D_6_: *δ*_H_ ≡ 7.16 ppm; CDCl_3_: *δ*_C_ ≡ 77.0 ppm; residual CHCl_3_ in CDCl_3_: *δ*_H_ ≡ 7.26 ppm).

#### Diethyl ((4*S*,5*S*)-5-((*tert*-butyldimethylsilyl)oxy)-4-methyl-3-oxo-7-(trimethylsilyl)hept-6-yn-2-yl)phosphonate (4)

To a solution of diethyl ethylphosphonate (1.02 g, 6.15 mmol) in THF (14 mL) at −78 °C was added a solution of *n*-butyllithium (2.46 mL, 2.5 M in hexanes) and the mixture was stirred for 20 min. Weinreb amide 5 ^15^ (1.0 g, 2.8 mmol) was then added and the reaction was stirred for 30 min before quenching with aq. NH_4_Cl (50 mL) and extracting with EtOAc (2 × 50 mL). The combined organic extracts were dried over MgSO_4_, filtered, and concentrated *in vacuo*. Purification by flash column chromatography on silica (1 : 1 hexanes : ethyl acetate) afforded phosponate 4 (1.1 g, 85%) as an oil.

##### Spectral data for the major diastereomer

IR (ATR): 3036, 29 078, 1710, 1635, 1460, 1364, 1191, 1077, 910, 778 cm^−1^. ^1^H NMR (500 MHz, CDCl_3_): *δ* 4.24 (d, *J* = 8.1 Hz, 1H), 4.13 (q, *J* = 6.4 Hz, 2H), 4.11 (q, *J* = 6.4 Hz, 2H), 3.70 (dq, *J*_HP_ = 26.0 Hz, *J*_HH_ = 6.7 Hz, 1H), 3.30 (m, 1H), 1.33 (d, *J* = 7.0 Hz, 3H), 1.32 (t, *J* = 7.0 Hz, 3H), 1.31 (t, *J* = 7.0 Hz, 3H), 1.14 (d, *J* = 6.6 Hz, 3H), 0.89 (s, 9H), 0.14 (s, 3H), 0.13 (s, 9H), 1.11 (s, 3H). ^13^C NMR (125 MHz, CDCl_3_): *δ* 207.1, 105.0, 91.2, 65.5, 62.5 (t, *J*_CP_ = 6.4 Hz, 1C), 54.2, 48.4, 47.5, 25.70, 25.67, 18.2, 16.4 (t, *J*_CP_ = 5.4 Hz, 1C), 13.2, 10.6 (*J*_CP_ = 6.2 Hz, 1C), −0.3, −0.4, −4.5, −5.1. HRMS-TOF (ESI+) calcd for C_21_H_43_O_5_PSi_2_Na^+^ (M + Na): 485.2284. Found 485.2279.

#### (5*S*,6*S*,8*E*,10*E*,12*S*)-2,2,3,3,6,8,12,15,15,16,16-Undecamethyl-5-((trimethylsilyl)ethynyl)-4,14-dioxa-3,15-disilaheptadeca-8,10-dien-7-one (7)

Ba(OH)_2_·8H_2_O (1.8 g, 5.73 mmol) was first activated by heating under vacuum at 130 °C for 1.5 h and then cooled to room temperature before adding THF (10 mL) and phosphonate 4 (1.1 g, 2.4 mmol) and the mixture was stirred for 30 min. Aldehyde 6 ^17^ (0.66 g, 2.9 mmol) was then added and the reaction was stirred for 8 h before quenching with aq. NaHCO_3_ (30 mL) and extracting with MTBE (2 × 30 mL). The combined organic extracts were dried over MgSO_4_, filtered, and concentrated *in vacuo*. Purification by flash column chromatography on silica (20 : 1 to 10 : 1 hexanes : ethyl acetate) afforded 7 (0.93 g, 72%) as an oil.

[*α*]^20^_D_ = −2.8 (*c* 1.0, CH_2_Cl_2_). IR (ATR) 3064, 2903, 1735, 1640, 1350, 1194, 1067, 910, 730 cm^−1^. ^1^H NMR (500 MHz, CDCl_3_): *δ* 7.07 (d, *J* = 10.9 Hz, 1H), 6.48 (ddd, *J* = 15.0, 10.9, 1.1 Hz, 1H), 6.08 (dd, *J* = 15.2, 7.5 Hz, 1H), 4.38 (d, *J* = 10.5 Hz, 1H), 3.60–3.47 (m, 3H), 2.50 (p, *J* = 6.6 Hz, 1H), 1.88 (s, 3H), 1.15 (d, *J* = 7.0 Hz, 3H), 1.06 (d, *J* = 7.0 Hz, 3H), 0.91 (s, 9H), 0.89 (s, 9H), 0.14 (s, 3H), 0.11 (s, 3H), 0.06 (s, 9H), 0.05 (s, 3H), 0.04 (s, 3H). ^13^C NMR (125 MHz, CDCl_3_): *δ*^13^C NMR (126 MHz, CDCl_3_) *δ* 203.5, 146.2, 139.3, 134.7, 126.3, 106.1, 90.1, 67.4, 65.7, 46.8, 40.3, 25.9, 25.8, 18.3, 16.3, 14.6, 11.8, −0.4, −4.5, −5.1, −5.3. HRMS-TOF (ESI+) calcd for C_29_H_56_O_3_Si_3_Na^+^ (M + Na): 559.3435. Found 559.3428.

#### (5*S*,6*R*,7*S*,8*E*,10*E*,12*S*)-2,2,3,3,6,8,12,15,15,16,16-Undecamethyl-5-((trimethylsilyl)ethynyl)-4,14-dioxa-3,15-disilaheptadeca-8,10-dien-7-ol

To a solution of ketone 7 (0.2 g, 0.37 mmol) in MeOH (1.0 mL) and THF (1.0 mL) at 0 °C was added NaBH_4_ (54 mg, 1.44 mmol) and the mixture was stirred and allowed to slowly warm to room temperature for 2 h. The reaction was quenched with brine (15 mL) and extracted with MTBE (2 × 15 mL). The combined organic extracts were dried over MgSO_4_, filtered, and concentrated *in vacuo*. Purification by flash column chromatography on silica (20 : 1 to 10 : 1 hexanes : ethyl acetate) afforded the alcohol (0.93 g, 72%) as an oil.

[*α*]^20^_D_ = −4.6 (*c* 1.0, CH_2_Cl_2_). IR (ATR) 3201, 2958, 2930, 2859, 1447, 1371, 1308, 1221, 1143, 1085, 1024, 755, 699 cm^−1^. ^1^H NMR (500 MHz, C_6_D_6_): *δ* 6.37 (ddd, *J* = 15.2, 10.8, 1.2 Hz, 1H), 6.13 (dd, *J* = 11.0, 1.5 Hz, 1H), 5.58 (dd, *J* = 15.2, 7.5 Hz, 1H), 5.03 (d, *J* = 2.5 Hz, 1H), 4.14 (dd, *J* = 9.0, 2.8 Hz, 1H), 3.49 (dd, *J* = 9.6, 5.9 Hz, 1H), 3.38 (dd, *J* = 9.6, 6.8 Hz, 1H), 2.41 (hept, *J* = 6.7 Hz, 1H), 2.12 (d, *J* = 2.9 Hz, O–H), 2.00 (dqd, *J* = 9.3, 6.9, 2.5 Hz, 1H), 1.64 (s, 3H), 1.08 (d, *J* = 7.0 Hz, 3H), 1.04 (d, *J* = 6.8 Hz, 3H), 1.02 (s, 9H), 0.97 (s, 9H) 0.29 (s, 3H), 0.19 (s, 3H), 0.17 (s, 9H), 0.05 (s, 6H). ^13^C NMR (125 MHz, C_6_D_6_): *δ* 137.3, 137.0, 126.1, 107.6, 90.0, 79.6, 68.2, 65.0, 43.0, 40.2, 31.9, 26.1, 26.0, 23.0, 18.52, 18.45, 16.9, 14.3, 11.6, 11.5, −0.1, −4.2, −5.0, −5.20, −5.24. HRMS-TOF (ESI+) calcd for C_29_H_58_O_3_Si_3_Na^+^ (M + Na): 561.3591. Found 561.3591.

#### (6*S*,7*E*,9*E*,11*S*,12*R*,13*S*)-13-Ethynyl-11-methoxy-2,2,3,3,6,10,12,15,15,16,16-undecamethyl-4,14-dioxa-3,15-disilaheptadeca-7,9-diene (8)

To a solution of the intermediate alcohol in THF (3.7 mL) at −78 °C was added a solution of LiHMDS (0.44 mL, 1.0 M in toluene) at −78 °C and the mixture was stirred for 20 min. Iodomethane (50 μL, 0.74 mmol) was then added and the reaction was allowed to slowly warm to room temperature over 15 h before quenching with NaHCO_3_ (30 mL) and extracting with MTBE (2 × 30 mL). The combined organic extracts were dried over MgSO_4_, filtered, and concentrated *in vacuo*. The crude methyl ether product was redissolved in MeOH (3.0 mL) and THF (1.0 mL) and the solution was cooled to 0 °C before adding K_2_CO_3_ (0.1 g, 0.72 mmol) and the mixture was slowly warmed to room temperature over 6 h. The reaction was quenched with aq. NH_4_Cl (50 mL) and extracted with MTBE (2 × 30 mL). The combined organic extracts were dried over MgSO_4_, filtered, and concentrated *in vacuo*. Purification by flash column chromatography on silica (20 : 1 to 10 : 1 hexanes : ethyl acetate) afforded 8 (0.11 g, 63% 3-steps) as an oil.

[*α*]^20^_D_ = −3.4 (*c* 1.0, CH_2_Cl_2_). IR (ATR) 2958, 2930, 2859, 1447, 1371, 1308, 1221, 1143, 1085, 1024, 755, 699 cm^−1^. ^1^H NMR (500 MHz, C_6_D_6_): *δ* 6.39 (dd, *J* = 15.2, 10.8 Hz, 1H), 6.09 (d, *J* = 10.0 Hz, 1H), 5.55 (dd, *J* = 15.2, 7.6 Hz, 1H), 5.22 (t, *J* = 2.0 Hz, 1H), 3.53 (d, *J* = 10.0 Hz, 1H), 3.46 (dd, *J* = 9.7, 6.1 Hz, 1H), 3.37 (dd, *J* = 9.7, 6.8 Hz, 1H), 3.09 (s, 3H), 2.40 (p, *J* = 6.9 Hz, 1H), 2.03 (d, *J* = 2.1 Hz, 1H), 2.02 (m, 1H), 1.59 (d, *J* = 1.3 Hz, 3H), 1.12 (d, *J* = 6.9 Hz, 3H), 1.04 (s, 9H), 1.02 (d, *J* = 6.7 Hz, 3H), 0.97 (s, 9H), 0.29 (s, 3H), 0.18 (s, 3H), 0.04 (s, 6H). ^13^C NMR (126 MHz, C_6_D_6_) *δ* 137.3, 133.6, 130.3, 125.5, 87.6, 85.7, 72.3, 67.8, 61.8, 55.2, 42.7, 39.9, 25.7, 18.2, 18.1, 16.4, 10.3, 10.0, −4.6, −5.5, −5.57, −5.59. HRMS-TOF (ESI+) calcd for C_27_H_52_O_3_Si_2_Na^+^ (M + Na): 503.3353. Found 503.3354.

#### (6*S*,7*E*,9*E*,11*S*,12*R*,13*S*)-13-((*E*)-2-Iodovinyl)-11-methoxy-2,2,3,3,6,10,12,15,15,16,16-undecamethyl-4,14-dioxa-3,15-disilaheptadeca-7,9-diene (2)

To a solution of alkyne 8 (0.1 g, 0.21 mmol) in THF (2.0 mL) was added zirconocene hydrochloride (30 mg, 0.1 mmol) and the mixture was stirred at room temperature for 20 min before adding additional zirconocene hydrochloride (30 mg, 0.1 mmol). After another 20 min at room temperature, iodine (66 mg, 0.26 mmol) was added and the solution was stirred for 30 min. The reaction was then diluted with hexanes (10 mL) and filtered through a pad of celite. The resulting solution was washed with sat. aq. Na_2_S_2_O_3_ (2 × 15 mL) and brine (15 mL). The organic phase was dried over MgSO_4_, filtered, and concentrated *in vacuo*. Purification by flash column chromatography on silica (10 : 1 hexanes : ethyl acetate) afforded 2 (0.1 g, 78%) as an oil.

[*α*]^20^_D_ = −7.8 (*c* 1.0, CH_2_Cl_2_). IR (ATR) 2955, 2928, 2865, 1450, 1370, 1310, 1235, 1121, 1065, 978, 884, 755, 699 cm^−1^. ^1^H NMR (500 MHz, CDCl_3_): *δ* 6.55 (dd, *J* = 14.4, 6.0 Hz, 1H), 6.29 (ddd, *J* = 15.2, 10.8, 1.2 Hz, 1H), 6.19 (dd, *J* = 14.4, 1.3 Hz, 1H), 5.89 (dd, *J* = 10.7, 1.5 Hz, 1H), 5.60 (dd, *J* = 15.3, 7.4 Hz, 1H), 4.62 (d, *J* = 6.0 Hz, 1H), 3.51 (dd, *J* = 9.7, 6.4 Hz, 1H), 3.43 (dd, *J* = 9.7, 6.9 Hz, 1H), 3.31 (d, *J* = 9.9 Hz, 1H), 3.11 (s, 3H), 2.40 (p, *J* = 6.7 Hz, 1H), 1.62 (m, 1H), 1.59 (s, 3H), 1.03 (d, *J* = 6.8 Hz, 3H), 0.92 (s, 9H), 0.89 (s, 9H), 0.63 (d, *J* = 7.0 Hz, 3H), 0.06 (s, 3H), 0.04 (s, 3H), 0.03 (s, 3H), 0.02 (s, 3H). ^13^C NMR (126 MHz, CDCl_3_): *δ* 149.2, 137.3, 133.4, 130.3, 125.4, 87.9, 75.5, 73.2, 67.9, 55.5, 41.2, 39.7, 29.7, 18.4, 18.2, 16.5, 10.5, 8.9, −4.1, −5.29, −5.32. HRMS-TOF (ESI+) calcd for C_27_H_53_IO_3_Si_2_Na^+^ (M + Na): 631.2476. Found 631.2476.

#### (*S*,*E*)-2,2,3,3,7,11,11,12,12-Nonamethyl-5-((*S*,3*Z*,5*E*)-4-methyl-6-(4,4,5,5-tetramethyl-1,3,2-dioxaborolan-2-yl)hepta-3,5-dien-2-yl)-4,10-dioxa-3,11-disilatridec-6-ene (10)

To a solution ^*t*^BuLi (0.1 mL, 1.7 M solution in pentane) in Et_2_O (1.0 mL) at −78 °C was added iodide 9 (50 mg, 0.08 mmol) and the mixture was stirred for 5 min before adding triisopropoxyborate (30 μL, 1.5 mmol) and the reaction was stirred for 30 min. Pinacol (20 mg, 2.0 mmol) was then added and the solution was warmed to room temperature and stirred for 15 h. The reaction was diluted with MTBE (20 mL) and washed with aq. NH_4_Cl (20 mL), water (20 mL), and brine (20 mL). The organic phase was dried over MgSO_4_, filtered, and concentrated *in vacuo*. Purification by flash column chromatography on silica (10 : 1 hexanes : ethyl acetate) afforded 10 (45 mg, 93%) as an oil.

[*α*]^20^_D_ = +3.2 (*c* 1.0, CH_2_Cl_2_). IR (ATR) 3056, 2973, 1640, 1565, 1350, 1194, 1067, 910, 730 cm^−1^. ^1^H NMR (500 MHz, CDCl_3_): *δ* 6.70 (s, 1H), 5.10 (dd, *J* = 9.1, 1.2 Hz, 1H), 5.05 (d, *J* = 9.8 Hz, 1H), 4.16 (dd, *J* = 9.0, 5.5 Hz, 1H), 3.60 (t, *J* = 6.2 Hz, 2H), 2.65 (ddd, *J* = 9.7, 6.9, 5.6 Hz, 1H), 1.97 (s, 2H), 1.88 (d, *J* = 1.6 Hz, 3H), 1.85 (d, *J* = 1.4 Hz, 3H), 1.57 (d, *J* = 1.4 Hz, 3H), 1.53–1.40 (m, 4H), 1.28 (s, 9H), 0.89 (s, 12H), 0.87 (d, *J* = 7.0 Hz, 3H), 0.85 (s, 9H), 0.04 (s, 6H), −0.01 (s, 3H), −0.04 (s, 3H). ^13^C NMR (125 MHz, CDCl_3_): *δ* 138.1, 135.3, 133.4, 132.7, 127.2, 83.3, 72.9, 63.0, 39.4, 39.4, 32.5, 26.0, 25.90, 25.86, 24.8, 24.7, 24.0, 23.9, 23.0, 18.4, 18.2, 16.7, 16.0, −4.3, −4.8, −5.3. HRMS-TOF (ESI+) calcd for C_34_H_67_BO_4_Si_2_Na^+^ (M + Na): 629.4569. Found 629.4572.

#### (2*S*,3*R*,*E*)-9-((*tert*-Butyldimethylsilyl)oxy)-1-((4-methoxybenzyl)oxy)-2,5-dimethylnon-4-en-3-yl 2-(diethoxyphosphoryl) propanoate (13)

To a solution of alcohol 11 (500 mg, 1.2 mmol) and phosphate 12 (249 mg, 1.2 mmol) in DCM (1.6 mL) at 0 °C was added DCC (293 mg, 1.42 mmol) and the solution was allowed to warm to room temperature and stir for 24 h. The reaction mixture was filtered through celite, diluted with H_2_O (10 mL), and extracted with DCM (2 × 10 mL). The combined organic extracts were dried over MgSO_4_, filtered and concentrated *in vacuo*. Purification by flash column chromatography on silica (4 : 1 to 1 : 1 to 1 : 2 hexanes : ethyl acetate) afforded ester 13 (604 mg, 82%) as an oil.

##### Spectral data for the mixture of diastereomers

IR (ATR) 2931, 2856, 1730, 1612, 1586, 1513, 1460, 1386, 1301, 1246, 1172, 1093, 1023, 962, 904, 834, 818, 774, 734, 661 cm^−1^. ^1^H NMR (500 MHz, CDCl_3_): *δ* 7.25–7.22 (m, 4H), 6.86 (d, *J* = 8.7 Hz, 4H), 5.61 (dd, *J* = 9.7, 6.5 Hz, 1H), 5.57 (dd, *J* = 9.7, 6.7 Hz, 1H), 5.09 (d, *J* = 9.7 Hz, 2H), 4.41 (d, *J* = 10.2 Hz, 2H), 4.37 (d, *J* = 10.2 Hz, 2H), 4.16–4.07 (m, 8H), 3.80 (s, 6H), 3.59 (t, *J* = 5.9 Hz, 4H), 3.42 (dd, *J* = 9.3, 5.8 Hz, 1H), 3.35 (dd, *J* = 9.3, 6.1 Hz, 1H), 3.30 (dd, *J* = 9.3, 6.3 Hz, 1H), 3.31 (dd, *J* = 9.3, 6.1 Hz, 1H), 2.95 (dq, *J* = 23.3, 7.4 Hz, 2H), 2.16 (m, 12H), 2.11 (m, 2H), 2.02 (m, 4H), 1.72 (s, 6H), 1.47–1.36 (m, 8H), 1.33–1.27 (m, 6H), 0.93 (d, *J* = 7.0 Hz, 3H), 0.92 (d, *J* = 7.0 Hz, 3H), 0.88 (s, 18H), 0.03 (s, 12H). ^13^C NMR (125 MHz, CDCl_3_): *δ* 159.1, 142.3, 130.6, 129.2, 120.4, 113.72, 113.70, 73.7, 72.7, 71.7, 62.9, 62.5, 55.3, 40.0, 39.5, 37.8, 32.41, 32.35, 26.0, 24.0, 18.4, 16.8, 12.7, 11.8, −5.3. HRMS-TOF (ESI+) calcd for C_32_H_57_O_8_PSiNa^+^ (M + Na): 651.3458. Found 651.3458.

#### (2*S*,3*R*,*E*)-9-((*tert*-Butyldimethylsilyl)oxy)-1-hydroxy-2,5-dimethylnon-4-en-3-yl-2-(diethoxyphosphoryl)propanoate

To a solution of 13 (250 mg, 0.398 mmol) in a DCM : H_2_O mixture (20 mL : 0.2 mL), DDQ (180 mg, 0.795 mmol) was added portion wise and stirred at room temperature for 1 h. The resulting solution was quenched with aqueous sodium bicarbonate (50 mL) and extracted with DCM (2 × 50 mL). The combined organic extracts were dried over MgSO_4_, filtered and concentrated *in vacuo*. Purification by flash column chromatography on silica (1 : 2 to 0 : 1 hexanes : ethyl acetate) afforded the corresponding alcohol (168 mg, 83%) as an oil.

##### Spectral data for the mixture of diastereomers

IR (ATR) 3406, 2928, 2856, 1471, 1455, 1384, 1360, 1253, 1100, 1081, 1030, 1002, 977, 935, 834, 773, 737, 661 cm^−1^. ^1^H NMR (500 MHz, CDCl_3_): *δ* 5.43 (dd, *J* = 8.7, 8.7 Hz, 1H), 5.39 (dd, *J* = 9.4, 9.4 Hz, 1H), 5.11 (d, *J* = 9.2 Hz, 1H), 5.06 (d, *J* = 9.8 Hz, 1H), 4.17–4.09 (m, 8H), 3.75 (dd, *J* = 11.5, 3.3 Hz, 1H), 3.64 (dd, *J* = 11.5, 3.8 Hz, 1H), 3.58 (t, *J* = 6.1 Hz, 4H), 3.50 (dd, *J* = 11.0, 4.9 Hz, 1H), 3.48 (dd, *J* = 11.5, 4.5 Hz, 1H), 2.99 (dq, *J* = 23.0, 7.5 Hz, 2H), 2.00 (m, 12H), 1.83 (m, 2H), 1.73 (d, *J* = 1.2 Hz, 3H), 1.71 (d, *J* = 1.2 Hz, 3H), 1.70 (m, 2H), 1.65 (m, 2H), 1.48–1.41 (m, 4H), 1.39 (d, *J* = 7.0 Hz, 3H), 1.35 (d, *J* = 7.0 Hz, 3H), 1.34–1.28 (m, 4H), 0.91 (d, *J* = 7.0 Hz, 3H), 0.89 (d, *J* = 7.0 Hz, 3H), 0.87 (s, 18H), 0.02 (s, 12H). ^13^C NMR (125 MHz, CDCl_3_): *δ* 169.3, 141.9, 122.3, 75.6, 75.1, 64.2, 62.9, 40.3, 39.4, 32.3, 26.0, 23.9, 18.3, 16.8, 16.4, 13.8, 11.7, −5.3. HRMS-TOF (ESI+) calcd for C_24_H_49_O_7_PSiNa^+^ (M + Na): 531.2883. Found 531.2883.

#### (5*S*,6*S*)-6-((*E*)-6-((*tert*-Butyldimethylsilyl)oxy)-2-methylhex-1-en-1-yl)-3,5-dimethyl-5,6-dihydro-2H-pyran-2-one (14)

To a solution of the alcohol (624 mg, 1.23 mmol) in DCM (6.13 mL) at 0 °C was added NaHCO_3_ (515 mg, 6.13 mmol) followed by the portionwise addition of Dess–Martin periodinane (520 mg, 1.23 mmol) and the resulting mixture was stirred for 1 h. The solution then was diluted with DCM (20 mL) and stirred vigorously with sodium thiosulfate pentahydrate (50 mL) for 30 min. The layers were separated and the aqueous phase was extracted with DCM (2 × 50 mL). The combined organic extracts were dried over MgSO_4_, filtered and concentrated *in vacuo*. The crude aldehyde (371 mg, 60%) was used directly in the next reaction.

Ba(OH)_2_·8H_2_O (340 mg, 1.1 mmol) was heated at 120 °C for 1.5 hours under vacuum and then allowed to cool to room temperature. THF (7 mL) was added to the flask and the solution was stirred for 10 minutes before adding the aldehyde (181 mg, 0.36 mmol) and the resulting mixture was stirred at room temperature for 15 h. The reaction was quenched with aq. NaHCO_3_ (20 mL) and extracted with MTBE (2 × 20 mL). The combined organic extracts were dried over MgSO_4_, filtered and concentrated *in vacuo*. Purification by flash column chromatography on silica (20 : 1 to 10 : 1 to 4 : 1 hexanes : ethyl acetate) afforded lactone 14 (72.2 mg, 57%) as an oil.

[*α*]^20^_D_ = −2.8 (*c* 1.0, CH_2_Cl_2_). IR (ATR) 2952, 2928, 2856, 1716, 1460, 1359, 1253, 1226, 1199, 1154, 1134, 1099, 1005, 981, 834, 810, 773, 731, 661. ^1^H NMR (500 MHz, CDCl_3_): *δ* 6.34 (s, 1H), 5.22 (d, *J* = 9.0 Hz, 1H), 4.68 (dd, *J* = 10.5, 9.0 Hz, 1H), 3.57 (m, 2H), 2.45 (ddtt, *J* = 14.6, 7.2, 4.8, 2.4 Hz, 1H), 2.03 (t, *J* = 5.4 Hz, 2H), 1.87 (dd, *J* = 2.4, 1.5 Hz, 3H), 1.67 (d, *J* = 1.5 Hz, 3H), 1.50–1.41 (m, 3H), 0.98 (d, *J* = 7.3 Hz, 3H), 0.85 (d, *J* = 6.0 Hz, 1H), 0.85 (s, 9H), 0.00 (s, 6H). ^13^C NMR (125 MHz, CDCl_3_): *δ* 165.96, 145.52, 143.82, 127.37, 121.50, 80.36, 62.86, 39.24, 34.59, 32.30, 25.95, 23.78, 18.31, 16.88, 15.94, −5.30. HRMS-TOF (ESI+) calcd for C_20_H_36_O_3_SiH^+^ (M + H): 353.2512. Found 353.2516.

#### (4*Z*,6*S*,7*S*,8*E*)-13-((*tert*-Butyldimethylsilyl)oxy)-4,6,9-trimethyl-2-(4,4,5,5-tetramethyl-1,3,2-dioxaborolan-2-yl)trideca-2,4,8-trien-7-ol (15)

To a solution of lactone 14 (175 mg, 0.498 mmol) in DCM (5 mL) at – 78 °C was added DIBAL-H (0.106 mL, 0.597 mmol) dropwise over 10 min and the resulting mixture was stirred for 1 hour. The reaction was quenched with the addition of aq. Rochelle's salt (2.0 M, 20 mL) and the solution was stirred vigorously for 1 h. The layers were separated and the aqueous phase was extracted with DCM (2 × 20 mL). The combined organic extracts were dried over MgSO_4_, filtered and concentrated *in vacuo*. The resulting lactol product (141 mg, 80%) could be used without further purification.

^1^H NMR (500 MHz, CDCl_3_): *δ* 5.48 (s, 1H), 5.16 (m, 2H), 4.24 (t, *J* = 9.3 Hz, 1H), 3.61 (t, *J* = 6.0 Hz, 3H), 2.89 (s, 1H), 2.09 (m, 1H), 2.06 (m, 2H), 1.73 (m, 3H), 1.71 (d, *J* = 1.5 Hz, 3H), 1.50 (m, 3H), 0.89 (s, 9H), 0.86 (d, *J* = 7.1 Hz, 3H), 0.04 (s, 6H). ^13^C NMR (125 MHz, CDCl_3_): *δ* 141.98, 132.03, 129.75, 12.65, 91.94, 69.63, 63.01, 39.38, 35.14, 32.41, 25.97, 23.96, 18.93, 18.35, 16.95, 16.38, −5.27.

To a solution of tetramethylpiperidine (137 mg, 0.97 mmol) in THF (0.5 mL) at −78 °C was added *n*-BuLi (0.388 mL, 2.5 M). The solution was allowed to warm to 0 °C before adding 1,1-bis(pinacolboronato)ethane (187 mg, 0.65 mmol). After 10 minutes, the lactol (114.6 mg, 0.32 mmol) was added and the solution was allowed to slowly warm to room temperature and stirred for 15 h. The solution was diluted with diethyl ether (10 mL), filtered through a silica plug, and concentrated *in vacuo*. Purification by flash column chromatography on silica (20 : 1 to 10 : 1 to 4 : 1 hexanes : ethyl acetate) afforded the vinyl boronate 15 (90 mg, 57%) as a 3 : 1 mixture of *Z* : *E* isomers.

##### Spectral data for *Z*-15

IR (ATR) 2977, 2933, 2879, 1460, 1305, 1268, 1215, 1142, 1105, 1016, 967, 846, 775, 736, 669. ^1^H NMR (500 MHz, CDCl_3_): *δ* 6.74 (s, 1H), 5.17–5.09 (m, 2H), 4.04 (dd, *J* = 8.9, 7.5 Hz, 1H), 3.61 (t, *J* = 5.9 Hz, 2H), 2.34 (m, 1H), 2.03 (m, 2H), 1.84 (s, 3H), 1.71 (s, 3H), 1.65 (s, 3H) 1.53–1.43 (m, 4H), 1.29 (s, 12H), 1.22 (d, *J* = 3.8 Hz, 3H), 0.89 (s, 9H), 0.04 (s, 6H). ^13^C NMR (125 MHz, CDCl_3_): *δ* 142.5, 139.7, 136.5, 130.3, 125.7, 83.1, 72.2, 63.1, 39.5, 32.5, 29.7, 26.0, 24.8, 24.0, 23.7, 18.4, 16.7, 16.2, 15.4, 10.4, −5.3. HRMS-TOF (ESI+) calcd for C_28_H_53_BO_4_SiNa^+^ (M + Na): 515.3704. Found 515.3706.

##### Spectral data for *E*-15

^1^H NMR (500 MHz, CDCl_3_): *δ* 6.68 (s, 1H), 5.13 (d, 9.24 Hz, 1H) 5.07 (d, *J* = 9.24 Hz, 1H), 4.01 (t, *J* = 8.46 Hz, 1H), 3.60 (m, 2H), 2.50 (m, 1H), 2.04 (d, *J* = 5.90 Hz, 2H), 1.88 (dd, *J* = 5.34, 1.34 Hz, 4H) 1.66 (s, 3H), 1.48 (m, 1H) 1.24 (d, *J* = 3.31 Hz, 3H), 1.21 (d, 4.15 Hz, 12H), 1.04 (d, *J* = 7.31 Hz, 2H), 0.89 (s, 9H), 0.84 (d, *J* = 6.73 Hz, 2H), 0.72 (q, *J* = 7.29 Hz, 1H) 0.04 (s, 6H). ^13^C NMR (125 MHz, CDCl_3_): *δ* 140.12, 139.32, 136.49, 130.68, 126.10, 82.85, 72.41, 63.01, 40.19, 39.50, 32.45, 25.94, 24.81, 24.51, 24.08, 23.94, 23.23, 18.31, 16.80, 9.03, −5.30.

#### Compound 1

To a degassed solution of vinyl boronate 9 (60 mg, 0.1 mmol) and vinyl iodide 2 (61 mg, 0.1 mmol) in DMF (1 mL) were added Pd(dppf)Cl_2_ (4 mg, 0.005 mmol) and Cs_2_CO_3_ (40 mg, 0.12 mmol) and the resulting solution was stirred at room temperature for 8 h. The reaction was diluted with MTBE (20 mL) and washed with water (20 mL). The layers were separated and the aqueous phase was extracted with MTBE (20 mL). The combined organic extracts were dried over MgSO_4_ and concentrated *in vacuo*. Purification by flash column chromatography on silica (20 : 1 to 10 : 1 hexanes : ethyl acetate) afforded the coupled product 1 (77 mg, 80%) as an oil.

[*α*]^20^_D_ = −5.2 (*c* 0.5, CH_2_Cl_2_). IR (ATR) 3062, 2983, 1736, 1614, 1415, 1274, 1267, 1129, 1078, 930 cm^−1^. ^1^H NMR (500 MHz, C_6_D_6_): *δ* 6.80 (d, *J* = 16.0 Hz, 1H), 6.44 (ddd, *J* = 15.3, 10.7, 0.9 Hz, 1H), 6.17 (d, *J* = 10.4 Hz, 1H), 6.02 (s, 1H), 5.90 (dd, *J* = 16.0, 7.2 Hz, 1H), 5.58 (dd, *J* = 15.4, 8.0 Hz, 1H), 5.34 (dd, *J* = 8.4, 0.8 Hz, 1H), 5.29 (ddd, *J* = 9.8, 2.0, 1.0 Hz, 1H), 5.07 (d, *J* = 7.4 Hz, 1H), 4.29 (dd, *J* = 9.0, 6.0 Hz, 1H), 3.63 (d, *J* = 10.0 Hz, 1H), 3.58–3.53 (m, 2H), 3.47 (dd, *J* = 9.7, 6.2 Hz, 1H), 3.38 (dd, *J* = 9.7, 6.8 Hz, 1H), 3.18 (s, 3H), 2.71 (m, 1H), 2.42 (m, 1H), 1.96 (m, 2H), 1.91 (s, 3H), 1.86 (m, 1H), 1.89 (d, *J* = 1.0 Hz, 3H), 1.71 (d, *J* = 1.0 Hz, 3H), 1.59 (s, 3H), 1.50 (m, 2H), 1.39 (m, 2H), 1.08 (s, 6H), 1.03 (s, 6H), 1.02 (s, 3H), 1.01 (s, 6H), 1.00 (s, 3H), 0.99 (d, *J* = 7.0 Hz, 3H), 0.98 (s, 3H), 0.96 (d, *J* = 7.0 Hz, 3H), 0.95 (s, 6H), 0.94 (d, *J* = 7.0 Hz, 3H), 0.93 (s, 3H), 0.19 (s, 3H), 0.17 (s, 3H), 0.13 (s, 6H), 0.08 (s, 6H), 0.05 (s, 6H). ^13^C NMR (125 MHz, C_6_D_6_): *δ*^13^C NMR (126 MHz, C_6_D6) *δ* 137.8, 136.0, 134.9, 134.7, 133.4, 133.3, 133.0, 132.5, 130.9, 130.3, 126.3, 125.4, 73.8, 72.9, 68.5, 63.4, 63.4, 56.0, 43.7, 41.5, 40.6, 40.1, 33.1, 31.4, 30.3, 28.5, 26.7, 26.6, 26.5, 25.4, 24.8, 24.8, 24.0, 21.0, 19.1, 18.9, 18.8, 17.4, 17.2, 17.1, 16.2, 14.3, 11.2, 9.8, 8.7, −3.0, −3.6, −4.2, −4.3, −4.4, −4.7, −4.8, −4.9. HRMS (ESI+): calcd for C_55_H_108_O_5_Si_4_Na^+^ (M + Na)^+^: 983.7172. Found 983.7179.

#### (*S*,*E*)-4-Methyl-1-(trimethylsilyl)pent-1-en-3-ol

Sodium bis(2-methoxyethoxy)aluminum hydride (2.65 mL, 3.07 M) was added dropwise to a solution of (*S*)-19 ^26^ (1.0313 g, 6.05 mmol) in diethyl ether (15 mL) at 0 °C and the mixture was stirred to room temperature for 15 hours. The reaction was quenched with ethyl acetate (50 mL) and stirred vigorously with aq. Rochelle's salt (50 mL, 2.0 M) for 1 hour. The layers were separated and the aqueous phase was extracted with ethyl acetate (2 × 50 mL). The combined organic fractions were dried over MgSO_4_, filtered through cotton, and concentrated under vacuum. Purification by flash chromatography on silica (20 : 1 to 10 : 1 hexanes : ethyl acetate) gave the *trans*-alkene product (0.859 g, 4.98 mmol, 82.4%) as an oil.

[*α*]^20^_D_ = +20.2 (*c* 1.0, CH_2_Cl_2_). IR (ATR): 3355, 3057, 3025, 2956, 2923, 1721, 1621, 1601, 1582, 1493, 1452, 1247, 1025, 989, 866, 835, 762, 742, 694. ^1^H NMR (500 MHz, CDCl_3_): *δ* 6.06 (dd, *J* = 9.5, 5.6 Hz, 1H), 5.88 (dd, *J* = 9.4, 1.3 Hz, 1H), 3.88 (s, 1H), 1.76 (o, *J* = 6.9 Hz, 1H), 0.94 (d, *J* = 7.1 Hz, 3H), 0.92 (d, *J* = 7.2 Hz, 3H), 0.10 (s, 9H). ^13^C NMR (500 MHz, CDCl_3_): *δ* 146.98, 130.48, 79.61, 33.55, 18.35, 17.64, −1.26. HRMS (ESI+): calcd for C_9_H_20_OSiNa^+^ (M + Na)^+^: 195.1181. Found 195.1180.

#### (*S*,*E*)-Triethyl((4-methyl-1-(trimethylsilyl)pent-1-en-3-yl)oxy)silane

Chlorotriethylsilane (0.117 mL, 0.696 mmol) was added to a solution of the alcohol (0.100 g, 0.580 mmol) and imidazole (0.0790 g, 1.16 mmol) in DCM (1.66 mL) at 0 °C and the resulting mixture was warmed to room temperature and stirred for 3 hours. The reaction was quenched with water (10 mL) and extracted with DCM (2 × 10 mL). The combined organic extracts were dried over MgSO_4_, filtered through cotton, and concentrated under vacuum. Purification by flash chromatography on silica (20 : 1 hexanes : ethyl acetate) gave the TES-protected alcohol (0.1453 g, 0.501 mmol, 86.3%) as an oil.

[*α*]^20^_D_ = +18.0 (*c* 1.0, CH_2_Cl_2_). IR (ATR): 2954, 2911, 2876, 1620, 1459, 1414, 1259, 1059, 1004, 992, 872, 834, 721, 691. ^1^H NMR (500 MHz, CDCl_3_): *δ* 5.97 (dd, *J* = 18.7, 6.5 Hz, 1H), 5.75 (dd, *J* = 18.8, 1.1 Hz, 1H), 3.77 (ddd, *J* = 6.4, 1.0 Hz, 1H), 1.66 (o, *J* = 6.5 Hz, 1H), 0.96 (t, *J* = 8.0 Hz, 9H), 0.90 (d, *J* = 6.8, 3H), 0.85 (d, *J* = 6.8 Hz, 3H), 0.59 (dq, *J* = 7.7, 2.5 Hz, 6H), 0.08 (s, 9H). ^13^C NMR (500 MHz, CDCl_3_): *δ* 147.91, 129.98, 81.17, 34.35, 18.39, 18.18, 6.88, 5.00, −1.32. HRMS (ESI+): calcd for C_15_H_34_OSi_2_Na^+^ (M + Na)^+^: 309.2046. Found 309.2045.

#### (*S*,*E*)-Triethyl((1-iodo-4-methylpent-1-en-3-yl)oxy)silane (18)

*N*-Iodosuccinimide (0.169 g, 0.751 mmol) was added to the vinyl silane (0.144 g, 0.501 mmol) and 2,6-lutidine (0.081 mL, 0.701 mmol) in 1,1,1,3,3,3-hexafluoroisopropanol (2.00 mL) at 0 °C and the resulting mixture was stirred for 15 minutes. The reaction was quenched with water (5 mL) and extracted with DCM (2 × 10 mL). The combined organic extracts were washed with sodium thiosulfate (25 mL), hydrochloric acid (25 mL, 1 M), water (25 mL), and sodium bicarbonate (25 mL), then dried over MgSO_4_, filtered through cotton, and concentrated under vacuum. Purification by flash chromatography on silica (20 : 1 hexanes : ethyl acetate) gave 18 (0.0885 g, 0.260 mmol, 52.0%) as an oil.

[*α*]^20^_D_ = +8.2 (*c* 0.5, CH_2_Cl_2_). IR (ATR): 2955, 2910, 2875, 1607, 1459, 1414, 1384, 1364, 1238, 1182, 1162, 1118, 1103, 1071, 1004, 948, 828, 723. ^1^H NMR (500 MHz, CDCl_3_): *δ* 6.53 (dd, *J* = 14.4, 6.8 Hz, 1H), 6.20 (dd, *J* = 14.4, 1.1 Hz, 1H), 3.83 (ddd, *J* = 6.7, 5.7, 1.1 Hz), 1.69 (do, *J* = 6.8, 1.1 Hz, 1H), 0.97 (t, *J* = 8.0 Hz, 9H), 0.91 (d, *J* = 6.8, 3H), 0.87 (d, *J* = 6.8, 3H), 0.60 (q, *J* = 8.2 Hz, 6H). ^13^C NMR (500 MHz, CDCl_3_): 147.81, 80.18, 76.26, 34.34, 18.01, 17.82, 6.82, 4.90. HRMS (ESI+): calcd for C_12_H_25_IOSiNa^+^ (M + Na)^+^: 363.0617. Found 363.0612.

#### (5*S*,6*E*,8*Z*,10*Z*,12*S*,13*S*,14*E*)-13-((*tert*-Butyldimethylsilyl)oxy)-3,3-diethyl-5-isopropyl-8,10,12,15,21,21,22,22-octamethyl-4,20-dioxa-3,21-disilatricosa-6,8,10,14-tetraene (17)

To a solution of vinyl boronate *E*-15 (100 mg, 0.2 mmol) and vinyl iodide 18 (69 mg, 0.2 mmol) in degassed DMF (1 mL) was added Pd(dppf)Cl_2_ (4 mg, 0.004 mmol) and Cs_2_CO_3_ (40 mg, 0.12 mmol) and the resulting solution was stirred for 15 h. The reaction mixture was diluted with MTBE (5 mL) and washed with water (20 mL). The aqueous phase was extracted with MTBE (2 × 20 mL). The combined organic extracts were dried with MgSO_4_ and concentrated *in vacuo*. Purification by flash column chromatography on silica (10 : 1 to 4 : 1 hexanes : ethyl acetate) afforded 17 (93 mg, 80%) as an oil. The product was unable to be purified away from a side product and this mixture was taken direction into the next reaction.

[*α*]^20^_D_ = −3.2 (*c* 0.5, CH_2_Cl_2_). IR (ATR) 2955, 2930, 2875, 1460, 1352, 1344, 1254, 1143, 1103, 1004, 969, 835, 774, 741, 724, 666. ^1^H NMR (500 MHz, CDCl_3_): *δ* 6.37 (d, *J* = 15.7 Hz, 1H), 5.84 (s, 1H), 5.64 (dd, *J* = 15.7, 7.9 Hz, 1H), 5.16 (d, 9.8 Hz, 1H), 5.10 (d, *J* = 8.8 Hz, 1H), 4.02 (t, *J* = 8.8 Hz, 1H), 3.82 (t, *J* = 7.0 Hz, 1H), 3.60 (t, *J* = 5.9 Hz, 2H), 2.34 (m, 1H), 2.02 (m, 2H), 1.86 (s, 3H), 1.84 (s, 3H), 1.67 (m, 1H), 1.66 (s, 3H), 1.53–1.43 (m, 4H), 1.24 (d, *J* = 3.8 Hz, 3H), 0.93 (t, *J* = 7.9 Hz, 9H), 0.89 (s, 9H), 0.84 (d, *J* = 5.8 Hz, 3H), 0.82 (d, *J* = 5.8 Hz, 3H), 0.56 (q, *J* = 7.9 Hz, 6H) 0.04 (s, 6H). ^13^C NMR (125 MHz, CDCl_3_): *δ* 139.8, 135.1, 132.4, 130.9, 129.0, 128.7, 125.7, 82.9, 72.3, 63.0, 40.5, 39.5, 35.1, 32.5, 26.0, 24.8, 24.6, 20.4, 18.4, 16.7, 16.4, 9.1, 6.9, 5.0, −5.3. HRMS (ESI+): calcd for C_34_H_66_O_3_Si_2_Na^+^ (M + Na)^+^: 601.4448. Found 601.4446.

#### (5*E*,7*S*,8*S*,9*Z*,11*Z*,13*E*,15*S*)-5,8,10,12,16-Pentamethylheptadeca-5,9,11,13-tetraene-1,7,15-triol (20)

To a solution of 17 (77 mg, 0.053 mmol) in THF (1.2 mL) and pyridine (0.3 mL) at 4 °C was added HF pyr (0.2 mL, 60% HF). The reaction mixture was kept at 4 °C for 15 h, and then quenched with aq. NaHCO_3_ (20 mL) and extracted with ethyl acetate (2 × 10 mL). The organic layers were dried over MgSO_4_ and concentrated *in vacuo*. Purification by flash column chromatography on silica (1 : 1 to 1 : 2 hexanes : ethyl acetate) afforded 20 (30.1 mg, 65%) as an oil.

[*α*]^20^_D_ = −2.8 (*c* 0.5, CH_2_Cl_2_). IR (ATR) 3338, 2957, 2929, 2870, 1667, 1638, 1585, 1447, 1378, 1366, 1328, 1257, 1203, 1175, 1136, 1106, 1065, 1003, 973, 935, 858, 817, 769, 736, 697, 667. ^1^H NMR (500 MHz, C_6_D_6_): *δ* 6.84 (d, *J* = 15.8, 1H), 5.78 (dd, *J* = 15.8, 6.6 Hz, 1H), 5.73 (s, 1H), 5.27–5.24 (m, 2H), 4.12 (t, *J* = 8.0 Hz, 1H), 3.90 (t, *J* = 6.2 Hz, 1H), 3.40 (m, 2H), 2.60 (m, 1H), 1.89 (m, 2H), 1.81 (s, 3H), 1.78 (s, 3H), 1.57 (s, 3H), 1.40–1.34 (m, 5H), 1.03 (d, *J* = 6.2 Hz, 3H) 0.95 (dd, *J* = 11.6, 7.1 Hz, 6H). ^13^C NMR (125 MHz, C_6_D_6_): *δ* 138.5, 133.6, 132.8, 132.0, 130.5, 129.1, 77.7, 72.5, 62.1, 40.4, 39.1, 34.1, 32.1, 24.4, 23.7, 19.9, 18.5, 18.1, 16.8, 16.4. HRMS (ESI+): calcd for C_22_H_38_O_3_Na^+^ (M + Na)^+^: 373.2719. Found 373.2711.

## Conflicts of interest

There are no conflicts to declare.

## Supplementary Material

RA-009-C9RA07050H-s001
