# Correction: Suzuki coupling-based synthesis of VATPase inhibitor archazolid natural product derived fragments

**DOI:** 10.1039/c9ra90080b

**Published:** 2019-10-29

**Authors:** Cooper A. Vincent, Evan T. Long, Holly C. Jones, Jeffrey C. Young, P. Clint Spiegel, Gregory W. O’Neil

**Affiliations:** Department of Chemistry, Western Washington University Bellingham WA USA 98229 oneilg@wwu.edu; Department of Biology, Western Washington University Bellingham WA USA 98229

## Abstract

Correction for ‘Suzuki coupling-based synthesis of VATPase inhibitor archazolid natural product derived fragments’ by Cooper T. Vincent *et al.*, *RSC Adv.*, 2019, **9**, 32210–32218.

The authors regret that the name of one of the authors (Cooper A. Vincent) was shown incorrectly in the original article. The corrected author list is as shown above.

The Royal Society of Chemistry apologises for these errors and any consequent inconvenience to authors and readers.

## Supplementary Material

