# Supplementary material for: Suzuki coupling-based synthesis of VATPase inhibitor archazolid natural product derived fragments
Source: RSC Adv. 2019 Oct 10;9(55):32210–8. doi: 10.1039/c9ra07050h (PMC9072946; doi:10.1039/c9ra07050h)

This journal is © The Royal Society of Chemistry 2019

Supplementary Material for

Suzuki coupling-based synthesis of VATPase inhibitor archazolid natural product derived fragments.

Cooper T. Vincent,[a] Evan T. Long,[a] Holly C. Jones,[a] Jeffrey C. Young,[b] P. Clint Spiegel,[a] and Gregory W. O'Neil*,[a]

[a]*Department of Chemistry, Western Washington University, Bellingham, WA, 98225*

[b]*Department of Biology, Western Washington University, Bellingham, WA, 98225*

**oneilg@wwu.edu*

Contents

Compound	^{1}H NMR spectrum	^{13}C NMR spectrum
**4**	2	2
**7**	3	3
**8**	5	5
**2**	6	6
**10**	7	7
**13**	8	8
**14**	10	10
**Z-15**	12	12
**E-15**	13	13
**1**	14	14
**18**	19	19
**17**	20	20
**20**	21	21
NOESY Spectrum of ***E*****-** and ***Z*****-15**	22	
**VATPase Assay**	23	
**COX Assay**	24	

General: All reactions were carried out under N_2 in flame-dried glassware. IR: Nicolet iS10 spectrometer, wavenumbers ($\tilde{\nu}$) in cm^{-1}. The solvents used were dried by passing the solvent through a column of activated alumina under nitrogen immediately prior to use. All reagents were purchased and used as received unless otherwise mentioned. All TLC analysis used 0.25 mm silica layer fluorescence UV_{254} plates. Flash chromatography: SilaCycle silica gel P60 (230-400 mesh). NMR: Spectra were recorded on a Varian Mercury 300, or Inova 500 spectrometer in the solvents indicated; chemical shifts (δ) are given in ppm, coupling constants (J) in Hz. The solvent signals were used as references (C_6D_6: $\delta_C \equiv 128.0$ ppm; residual C_6H_6 in C_6D_6: $\delta_H \equiv 7.16$ ppm; $CDCl_3$: $\delta_C \equiv 77.0$ ppm; residual $CHCl_3$ in $CDCl_3$: $\delta_H \equiv 7.26$ ppm).

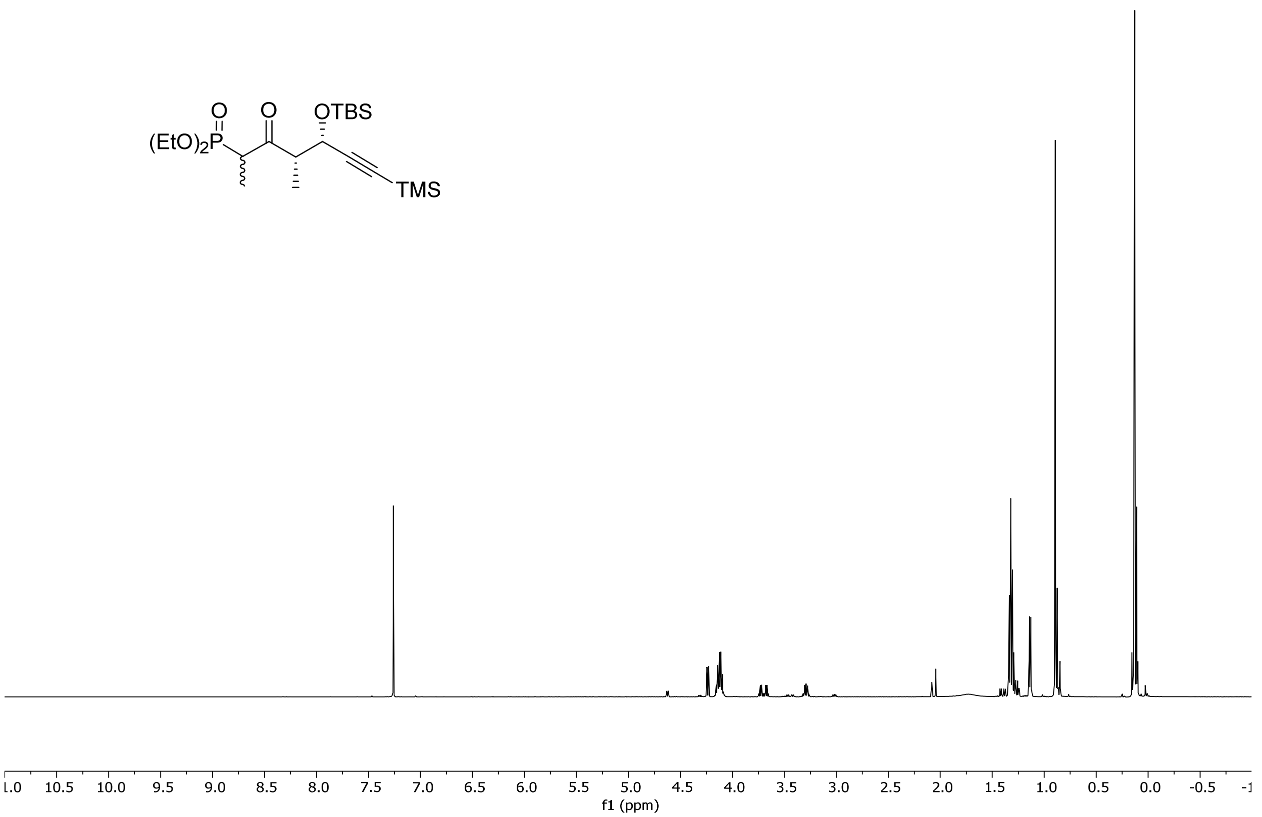
O
O
OTBS
(EtO)2P
TMS
1.0 10.5 10.0 9.5 9.0 8.5 8.0 7.5 7.0 6.5 6.0 5.5 5.0 4.5 4.0 3.5 3.0 2.5 2.0 1.5 1.0 0.5 0.0 -0.5
f1 (ppm)

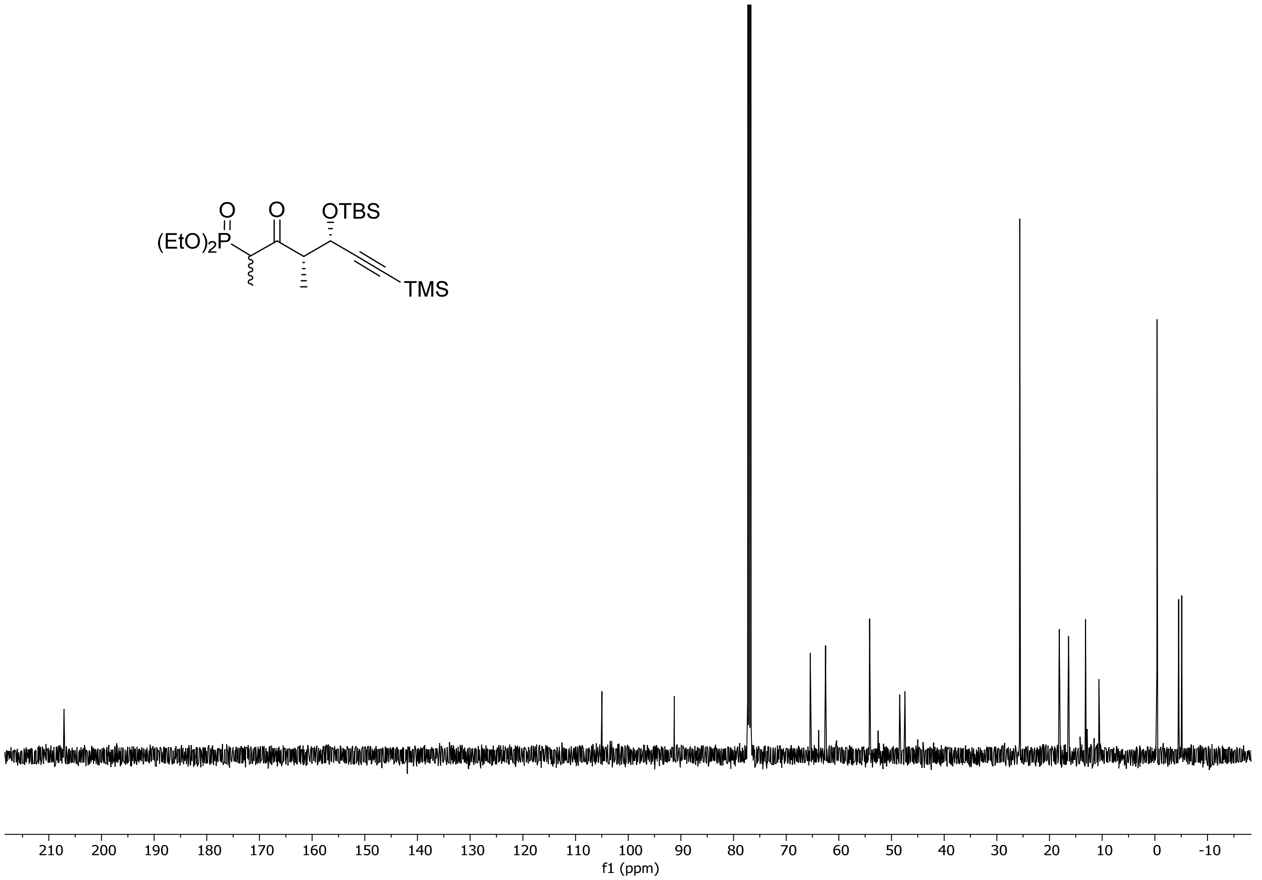
O
O
OTBS
(EtO)2P
TMS
210 200 190 180 170 160 150 140 130 120 110 100 90 80 70 60 50 40 30 20 10 0 -10
f1 (ppm)

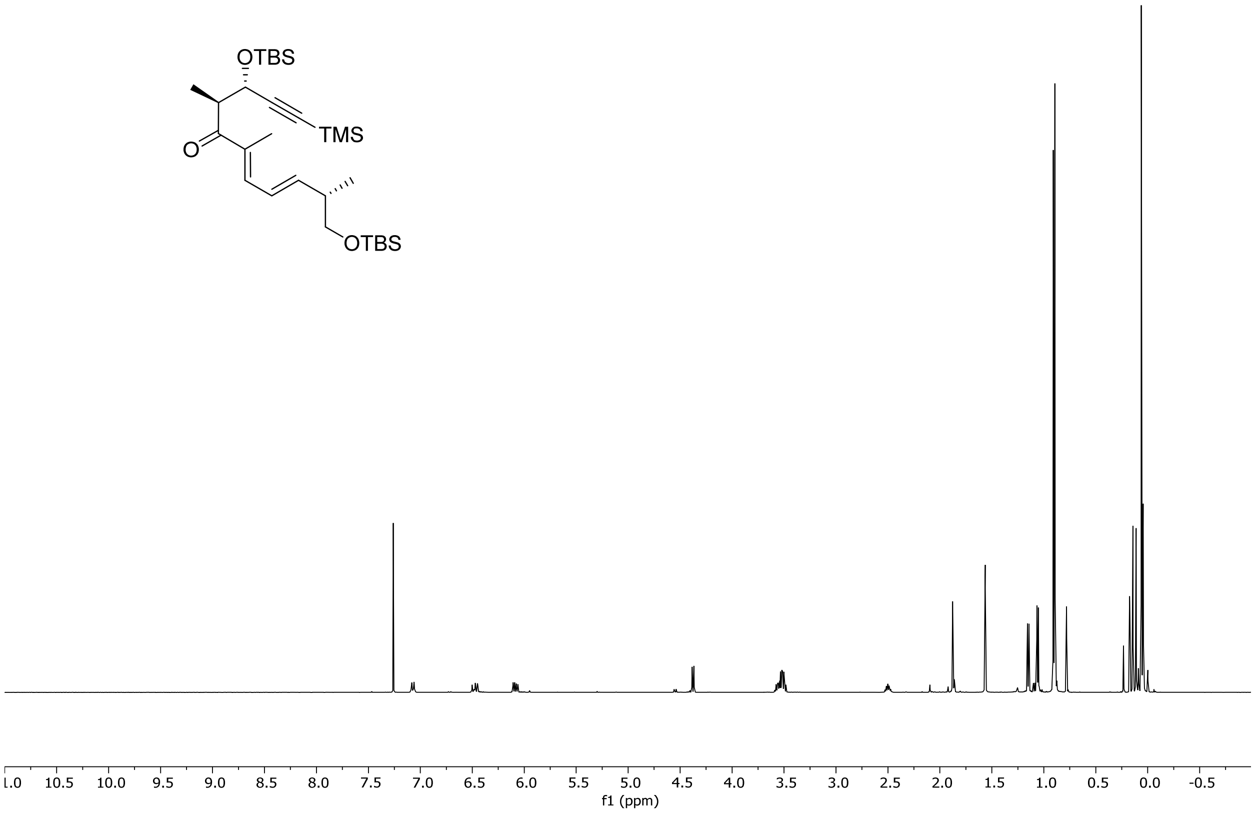
OTBS
TMS
O
OTBS
1.0 10.5 10.0 9.5 9.0 8.5 8.0 7.5 7.0 6.5 6.0 5.5 5.0 4.5 4.0 3.5 3.0 2.5 2.0 1.5 1.0 0.5 0.0 -0.5
f1 (ppm)

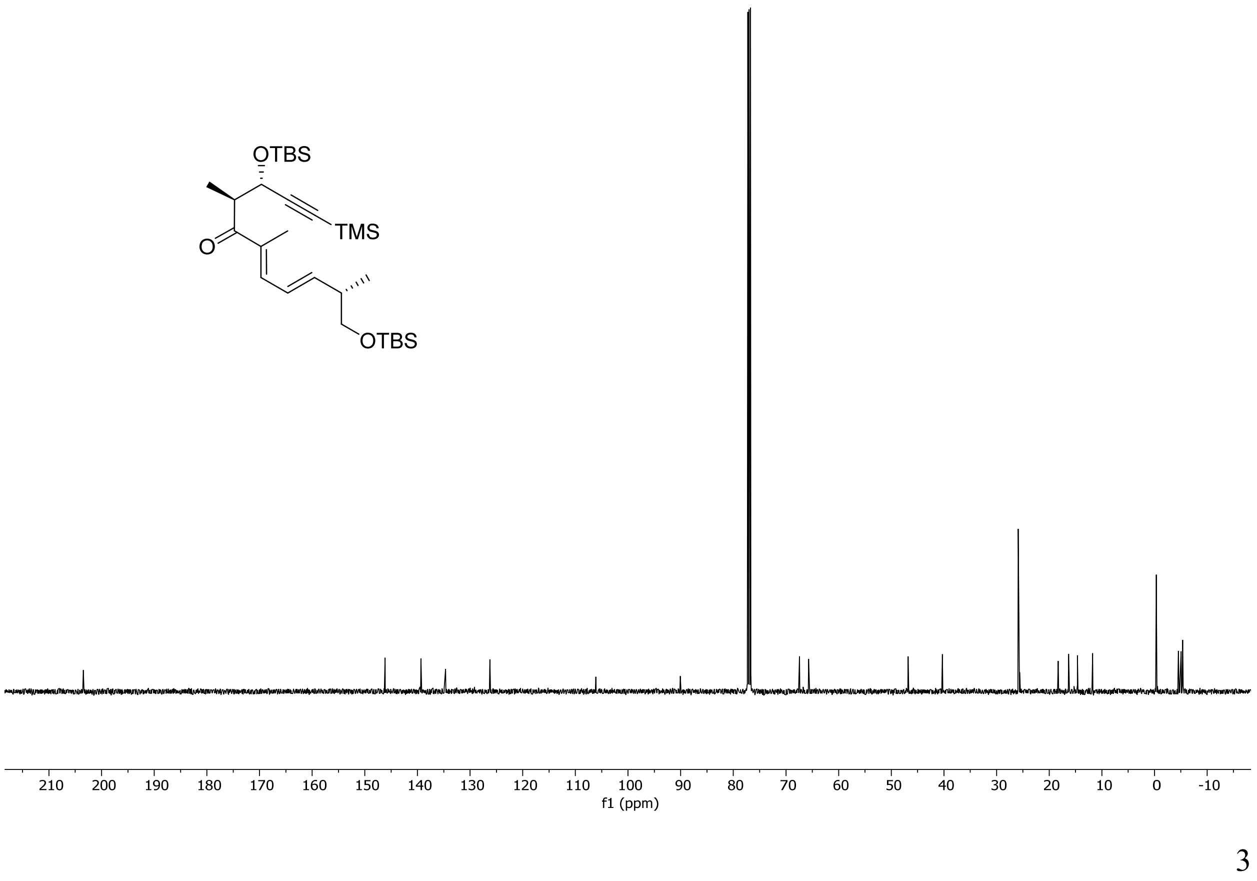
OTBS
TMS
O
OTBS
210 200 190 180 170 160 150 140 130 120 110 100 90 80 70 60 50 40 30 20 10 0 -10
f1 (ppm)

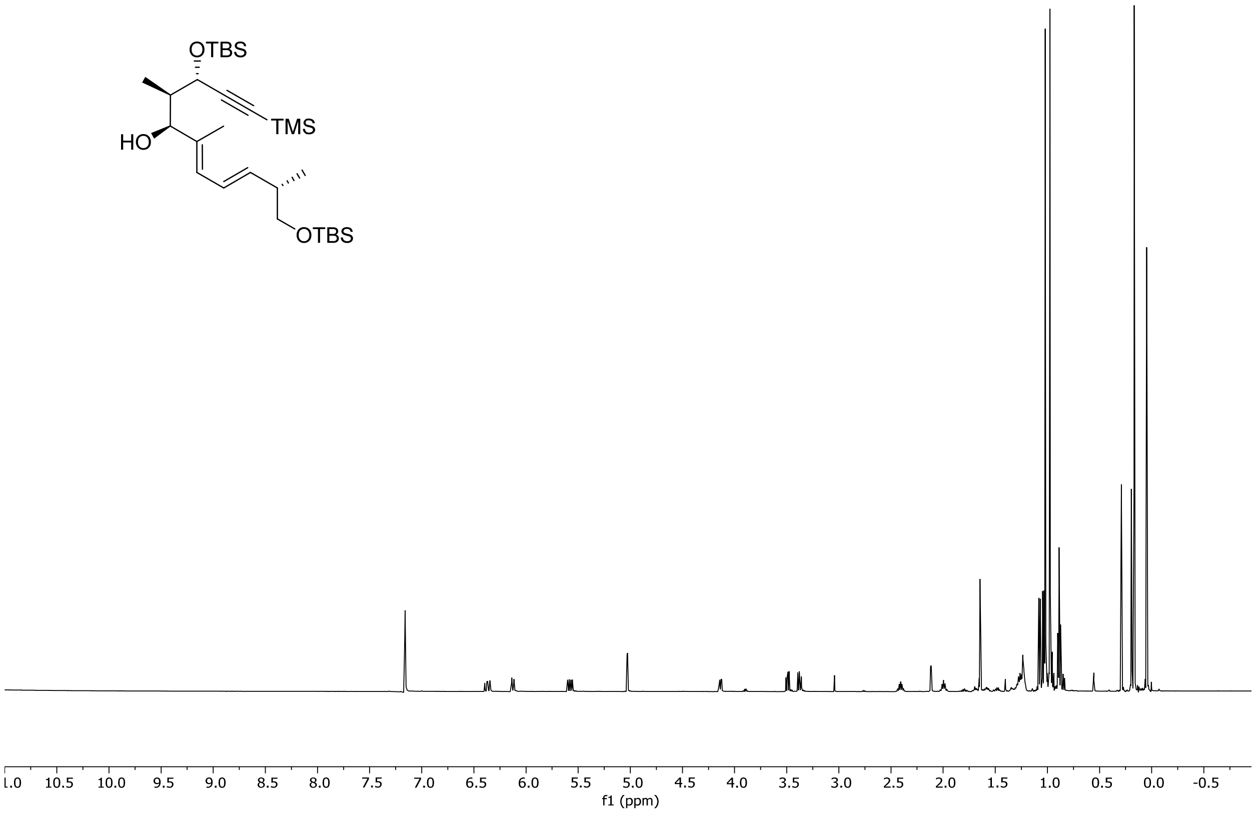
OTBS
TMS
HO
OTBS
f1 (ppm)

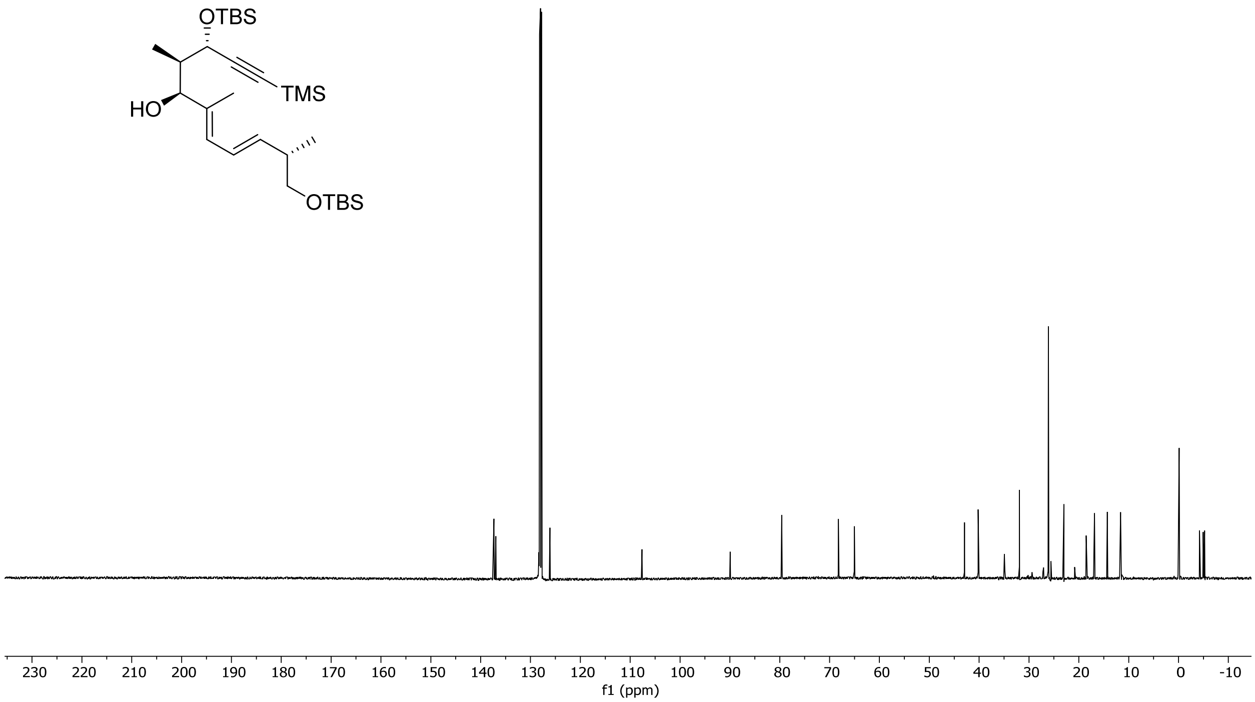
OTBS
TMS
HO
OTBS
f1 (ppm)

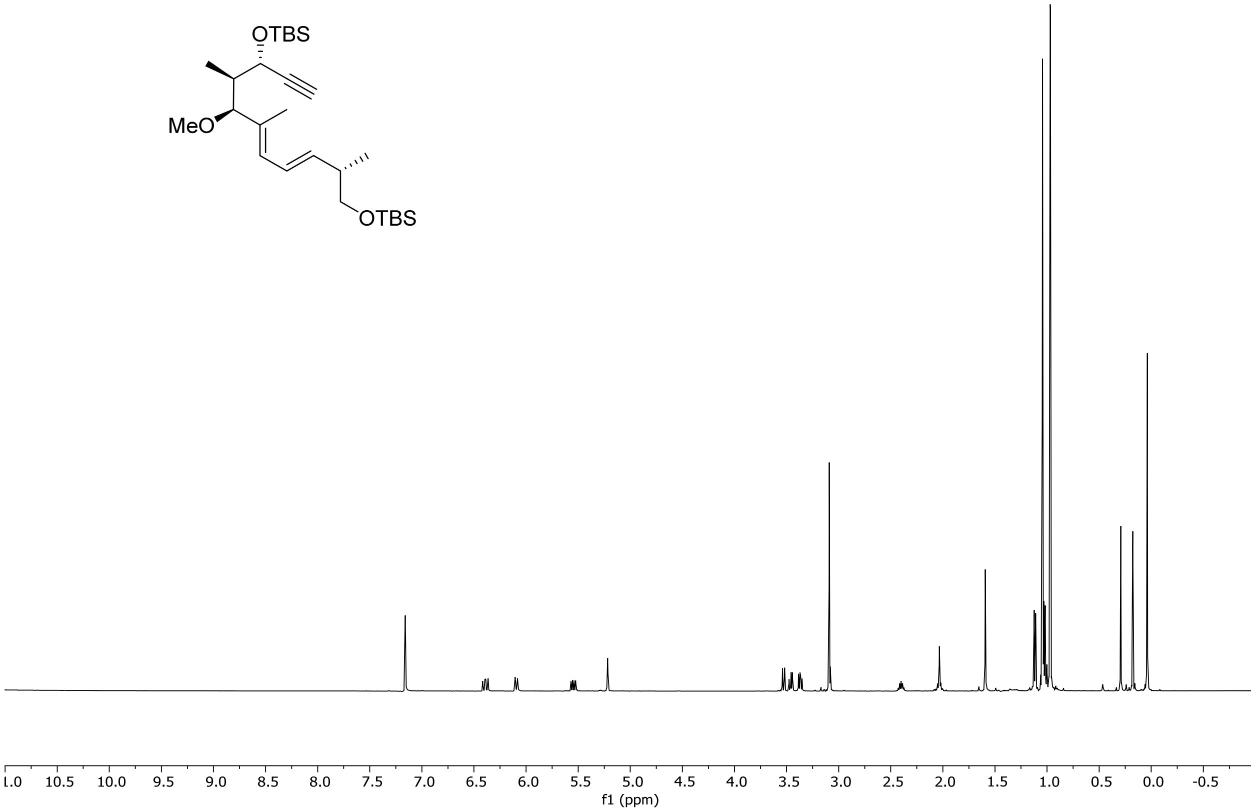
OTBS
MeO
OTBS
f1 (ppm)

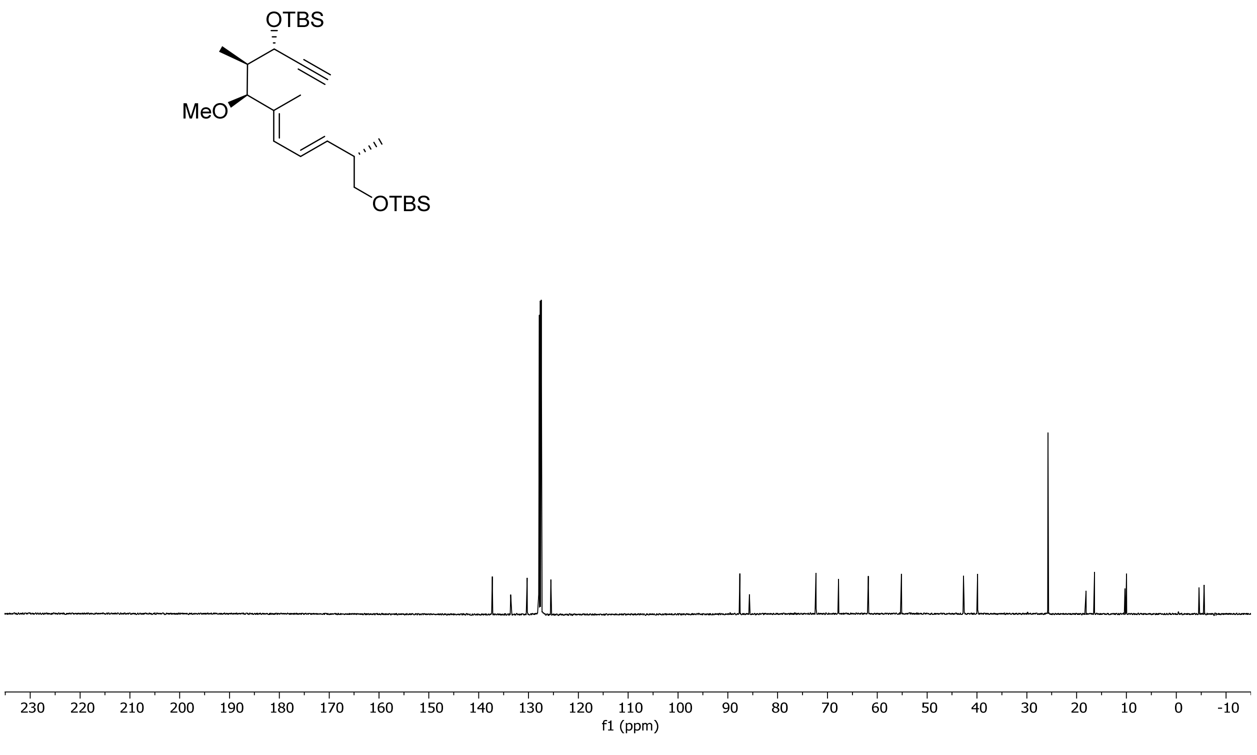
OTBS
MeO
OTBS
f1 (ppm)

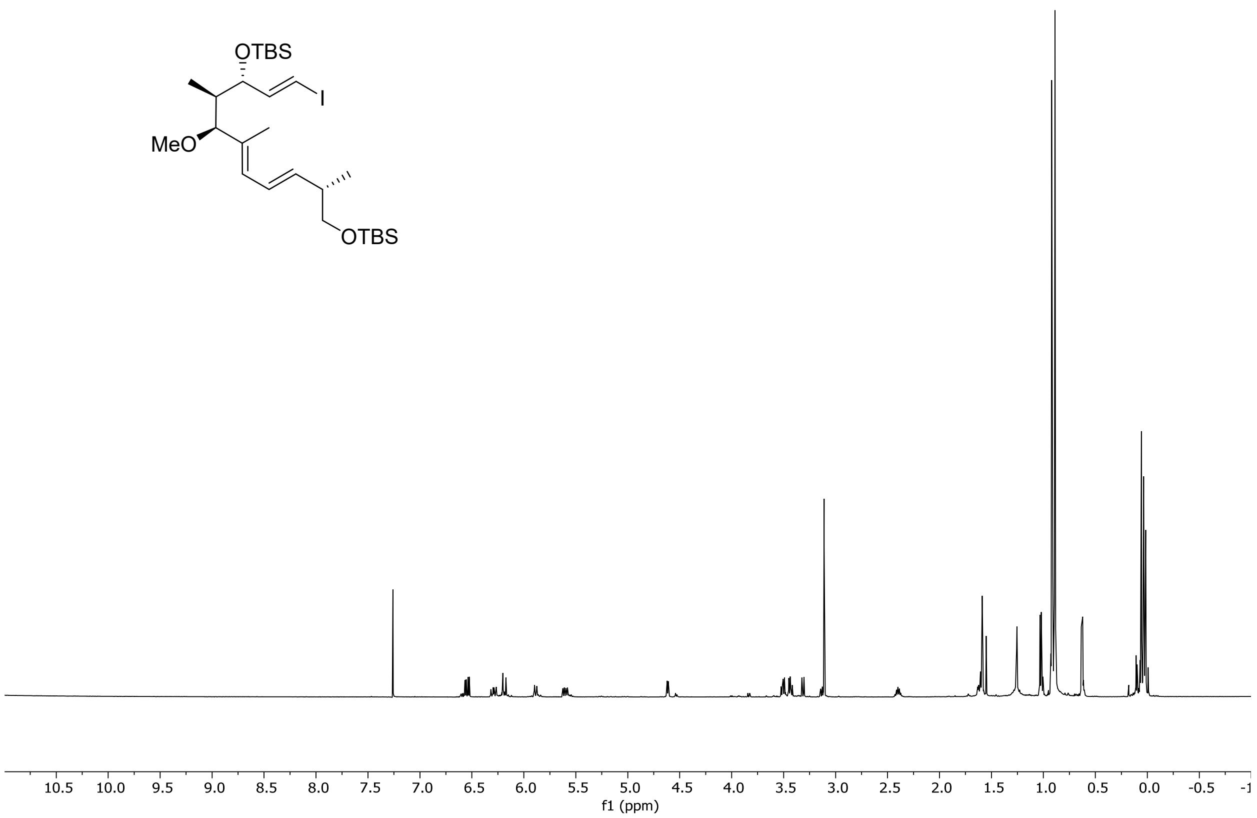
OTBS
I
MeO
OTBS
10.5 10.0 9.5 9.0 8.5 8.0 7.5 7.0 6.5 6.0 5.5 5.0 4.5 4.0 3.5 3.0 2.5 2.0 1.5 1.0 0.5 0.0 -0.5
f1 (ppm)

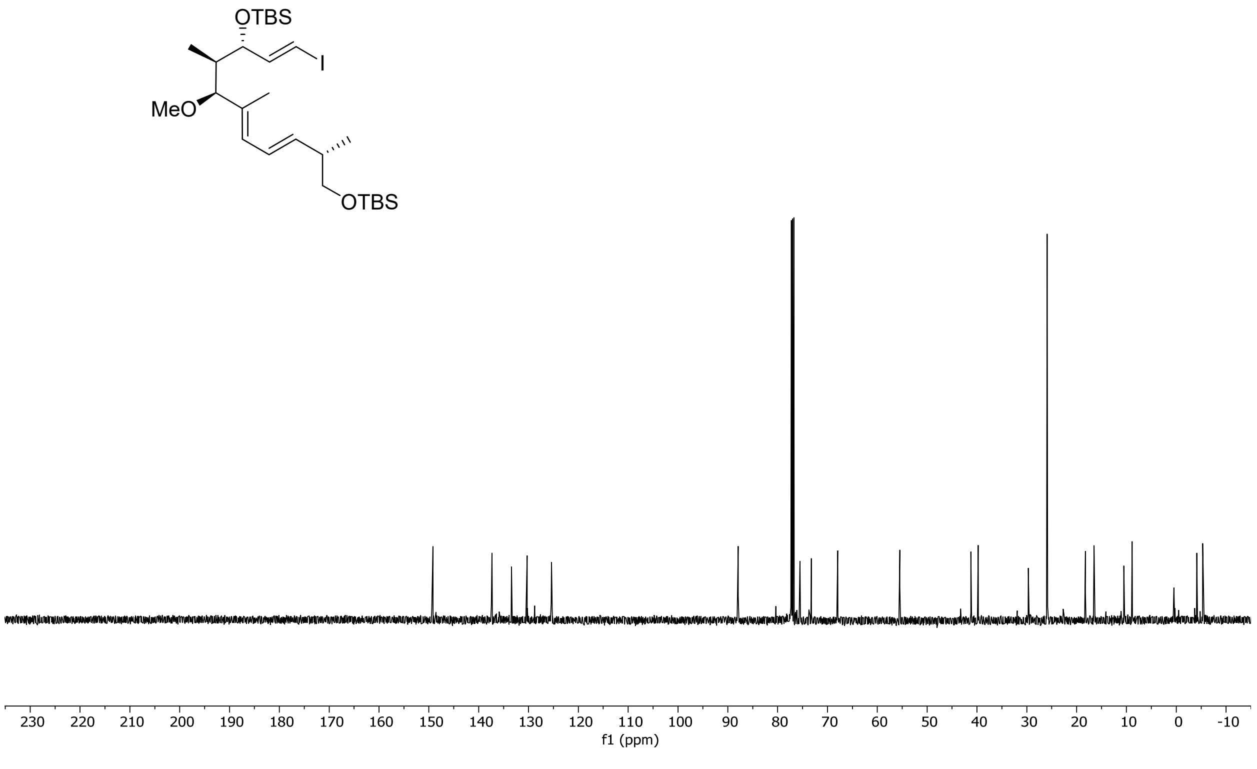
OTBS
I
MeO
OTBS
230 220 210 200 190 180 170 160 150 140 130 120 110 100 90 80 70 60 50 40 30 20 10 0 -10
f1 (ppm)

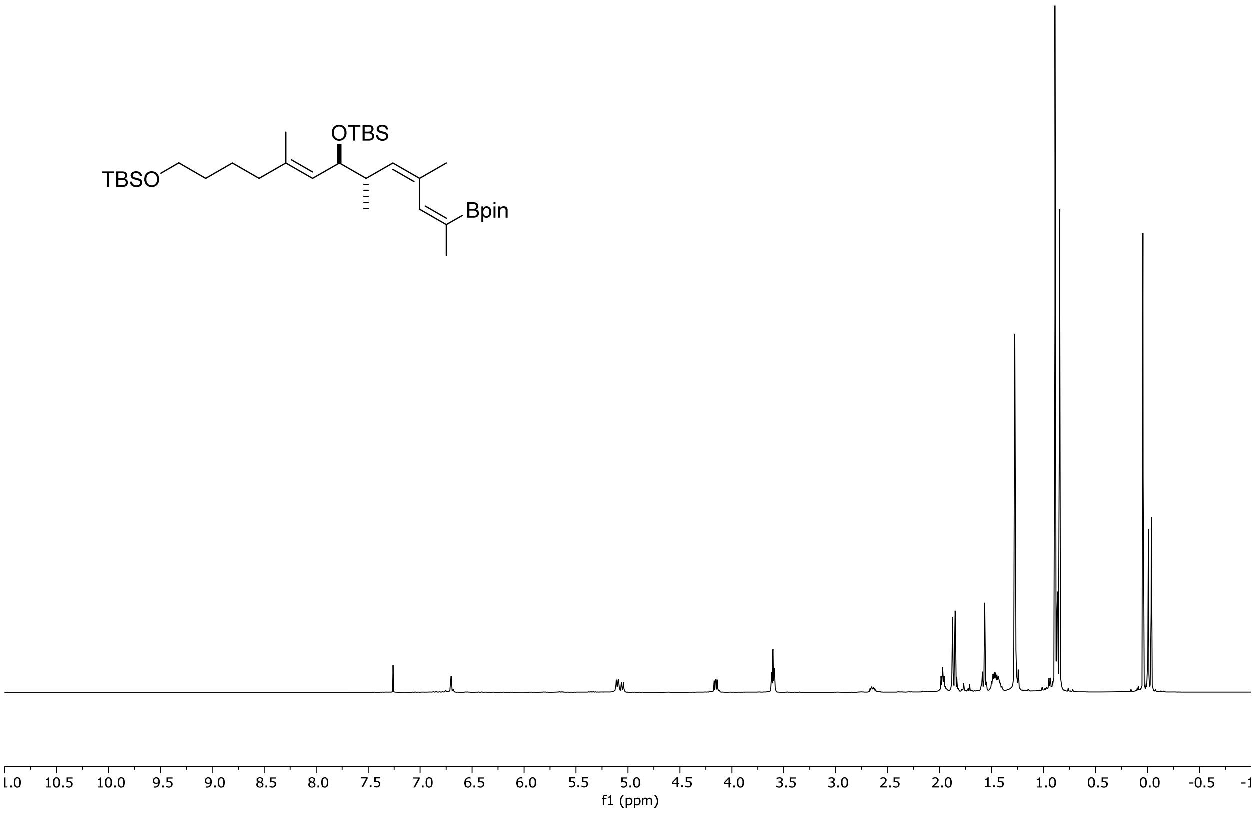

TBSO
OTBS
Bpin
f1 (ppm)

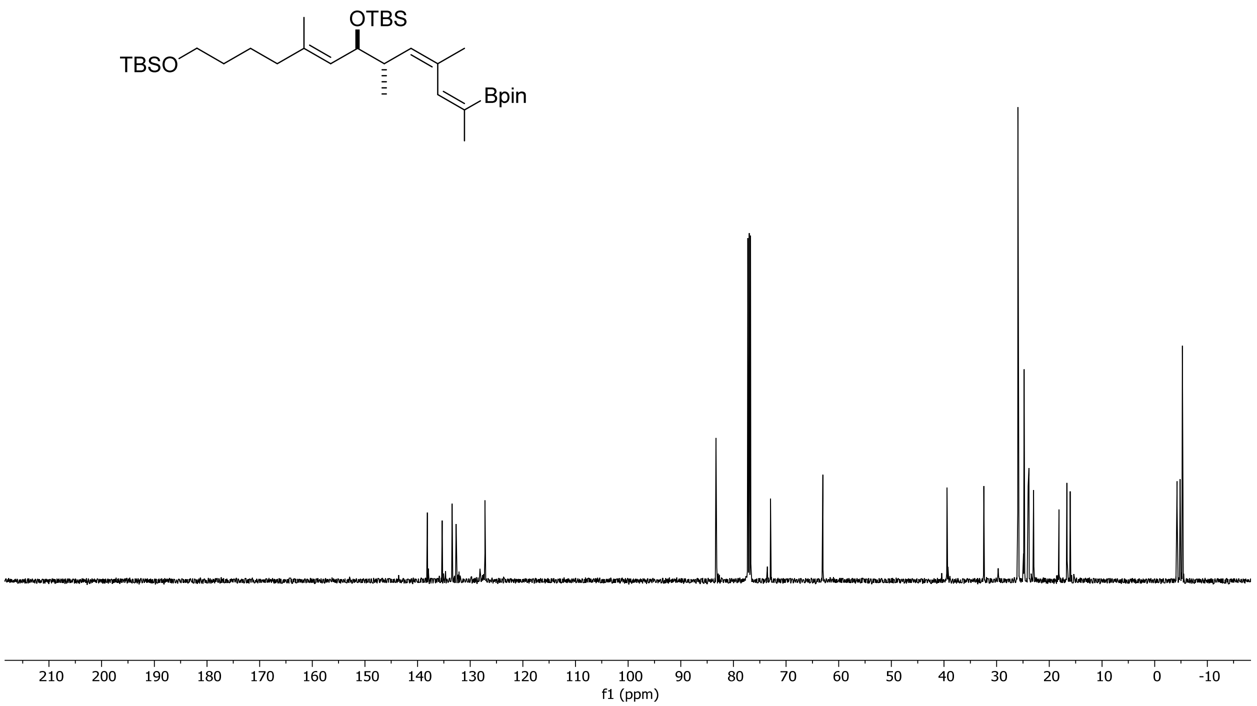

TBSO
OTBS
Bpin
f1 (ppm)

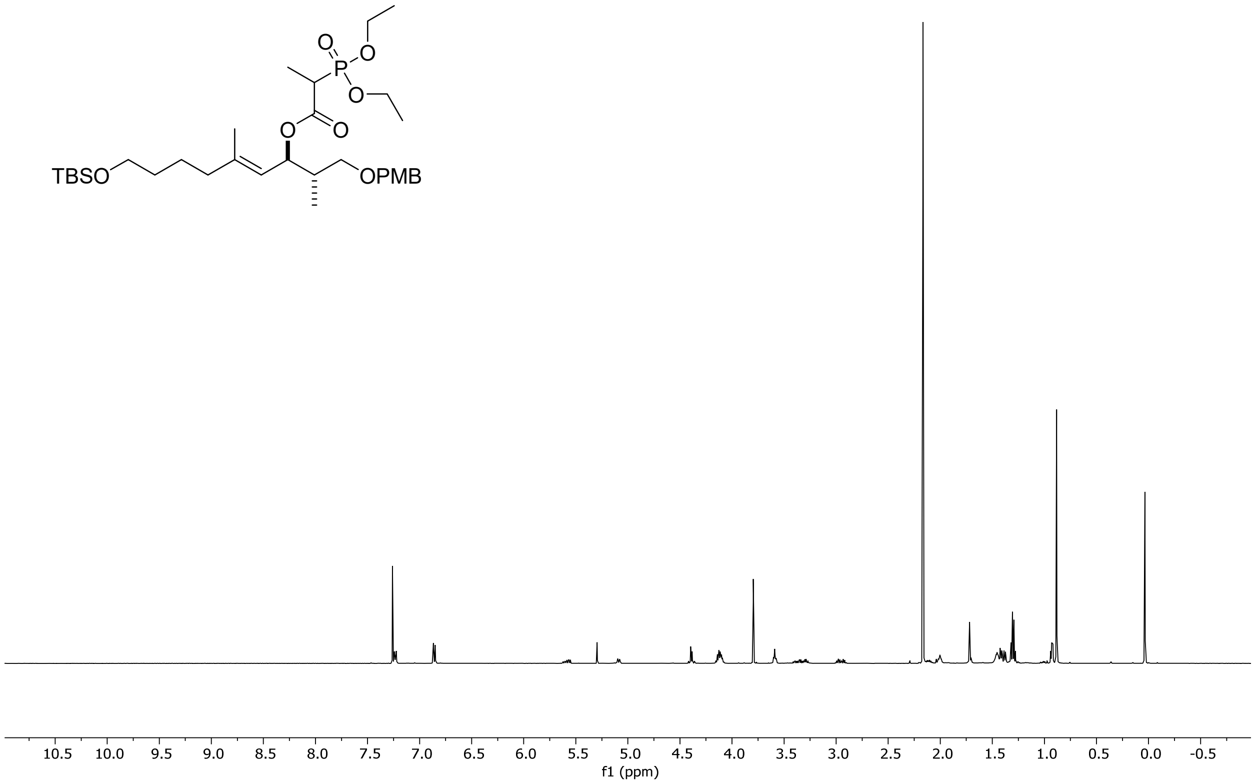
TBSO
OPMB
O
P
10.5 10.0 9.5 9.0 8.5 8.0 7.5 7.0 6.5 6.0 5.5 5.0 4.5 4.0 3.5 3.0 2.5 2.0 1.5 1.0 0.5 0.0 -0.5
f1 (ppm)

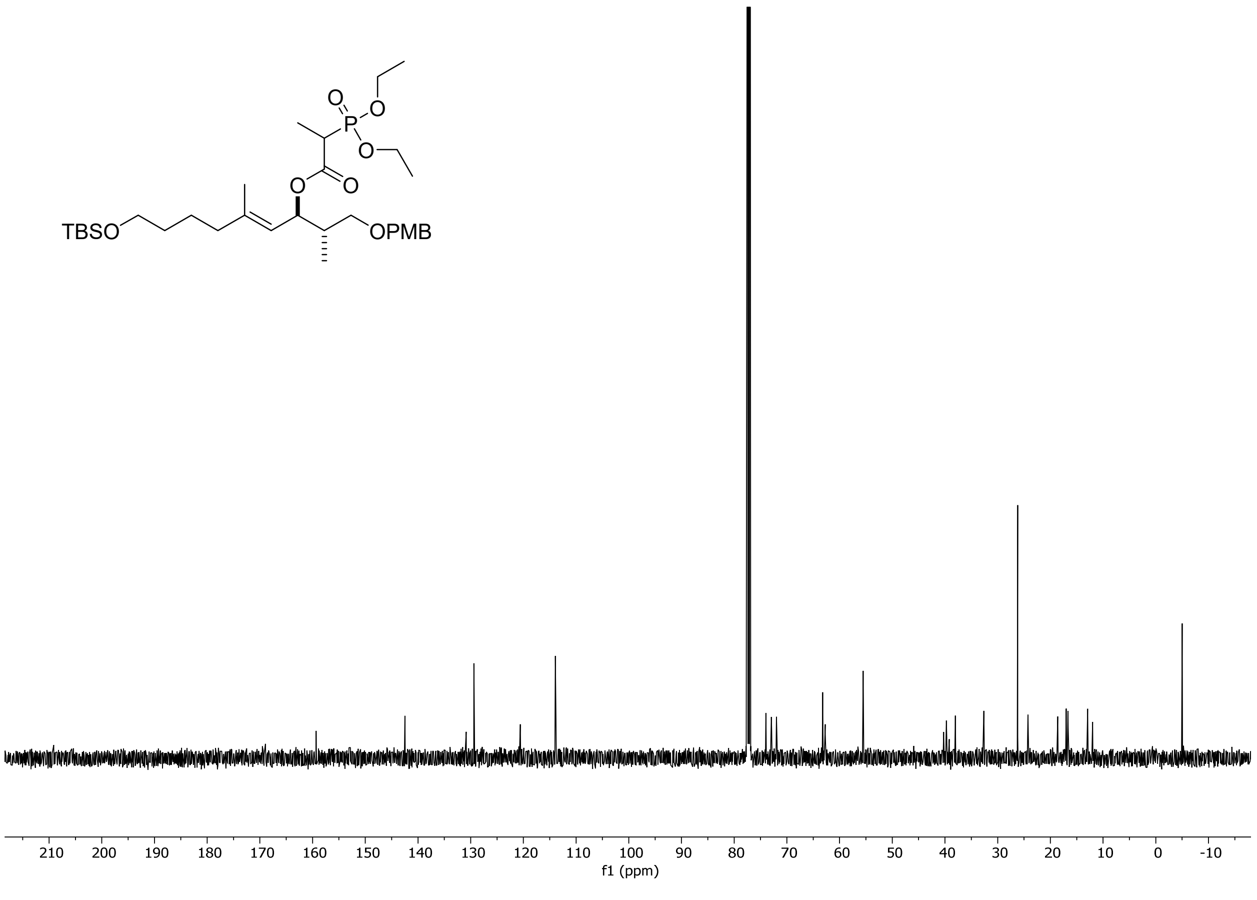
TBSO
OPMB
O
P
210 200 190 180 170 160 150 140 130 120 110 100 90 80 70 60 50 40 30 20 10 0 -10
f1 (ppm)

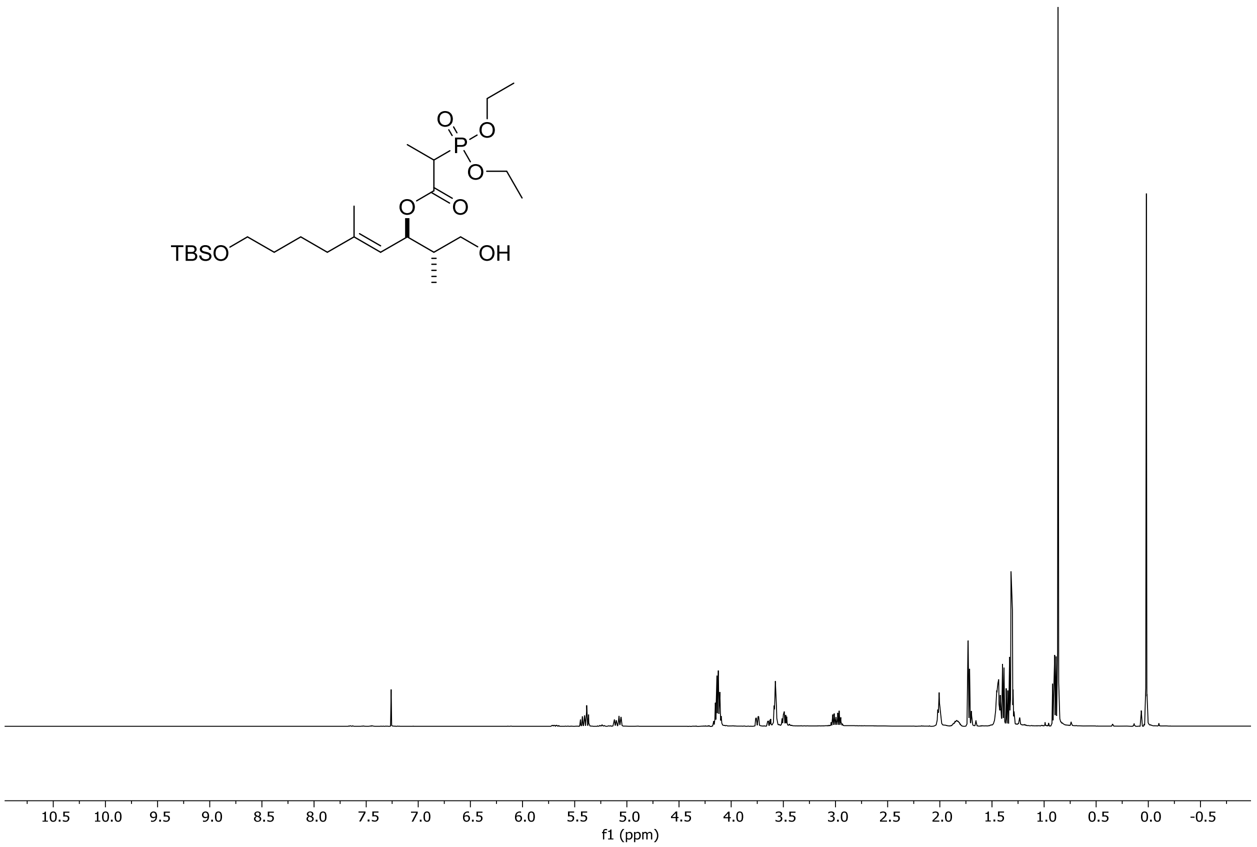
TBSO
OH
10.5 10.0 9.5 9.0 8.5 8.0 7.5 7.0 6.5 6.0 5.5 5.0 4.5 4.0 3.5 3.0 2.5 2.0 1.5 1.0 0.5 0.0 -0.5
f1 (ppm)

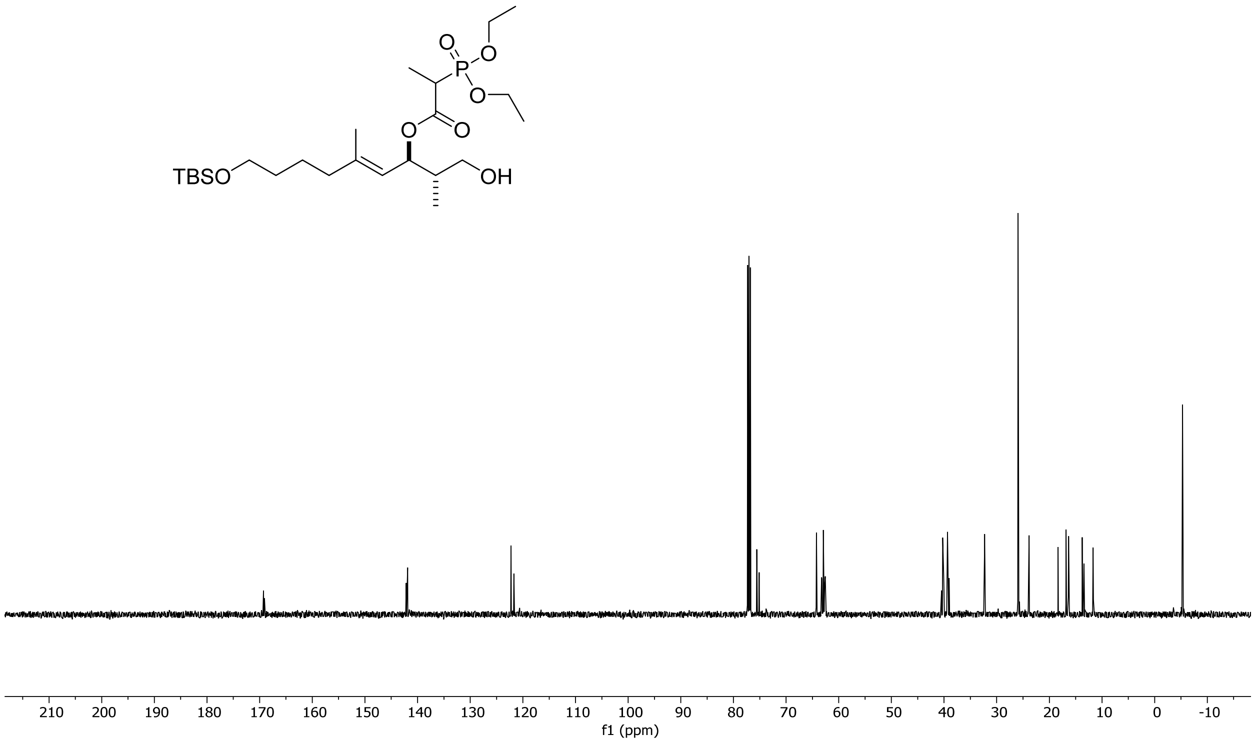
TBSO
OH
210 200 190 180 170 160 150 140 130 120 110 100 90 80 70 60 50 40 30 20 10 0 -10
f1 (ppm)

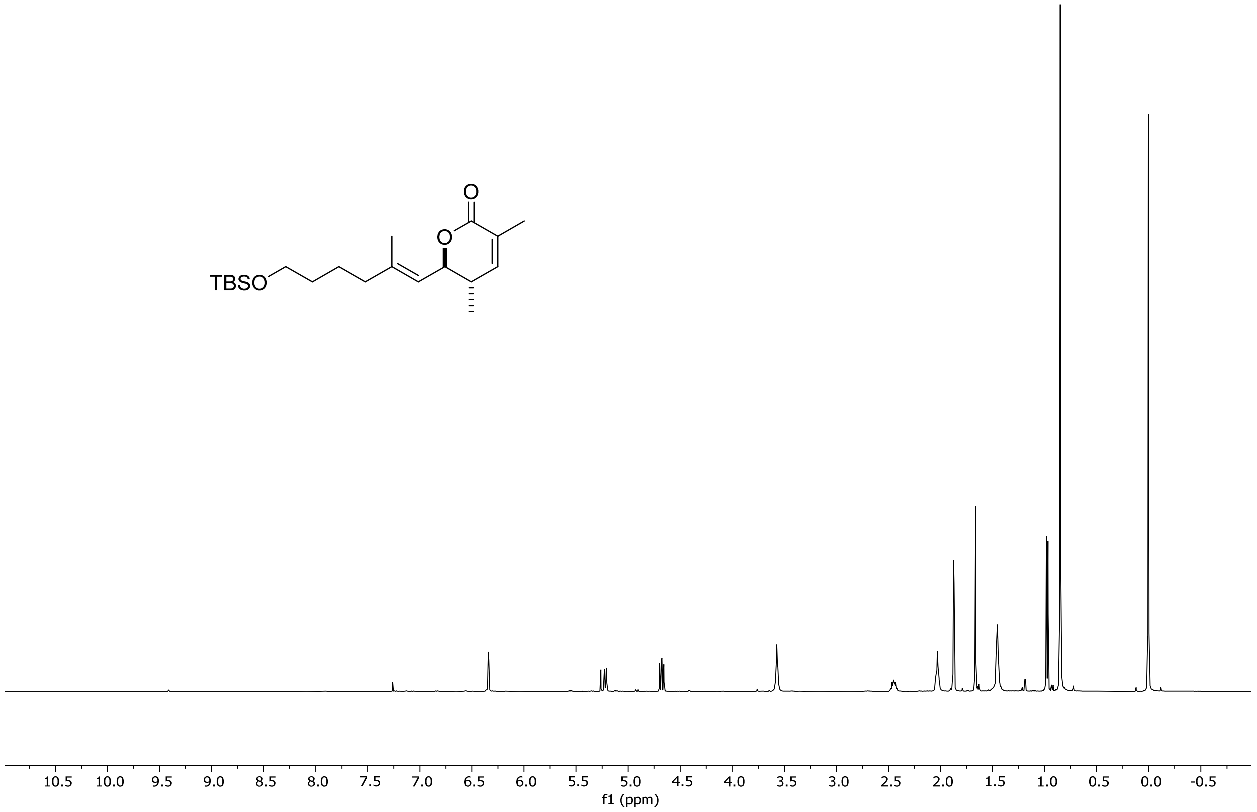
TBSO
O
O
10.5 10.0 9.5 9.0 8.5 8.0 7.5 7.0 6.5 6.0 5.5 5.0 4.5 4.0 3.5 3.0 2.5 2.0 1.5 1.0 0.5 0.0 -0.5
f1 (ppm)

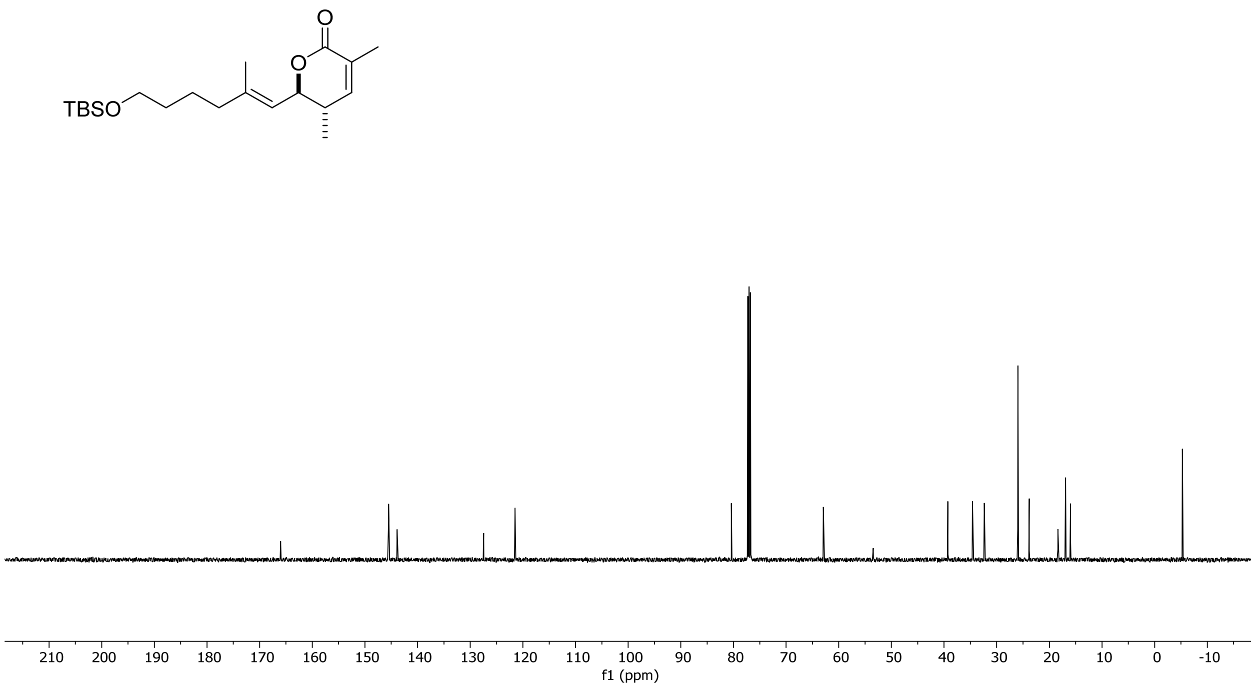
TBSO
O
O
210 200 190 180 170 160 150 140 130 120 110 100 90 80 70 60 50 40 30 20 10 0 -10
f1 (ppm)

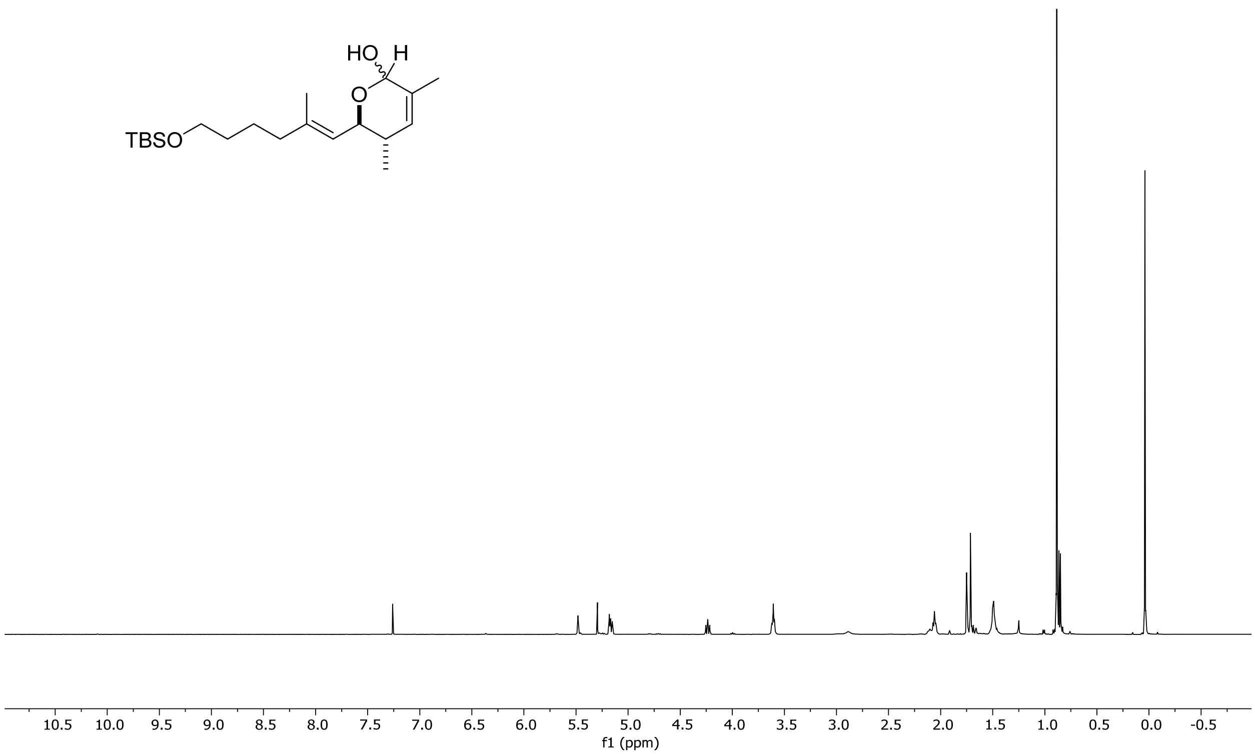
HO
H
O
TBSO
10.5
10.0
9.5
9.0
8.5
8.0
7.5
7.0
6.5
6.0
5.5
5.0
4.5
4.0
3.5
3.0
2.5
2.0
1.5
1.0
0.5
0.0
-0.5
f1 (ppm)

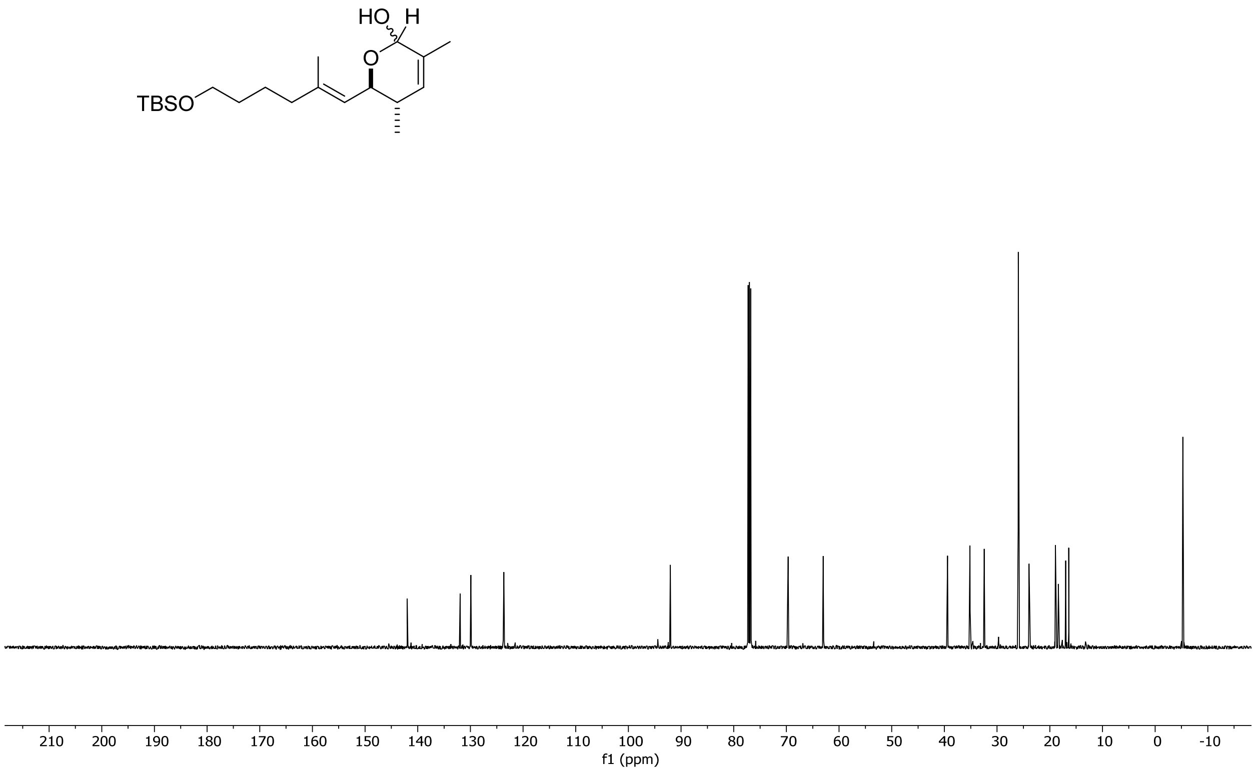
HO
H
O
TBSO
210
200
190
180
170
160
150
140
130
120
110
100
90
80
70
60
50
40
30
20
10
0
-10
f1 (ppm)

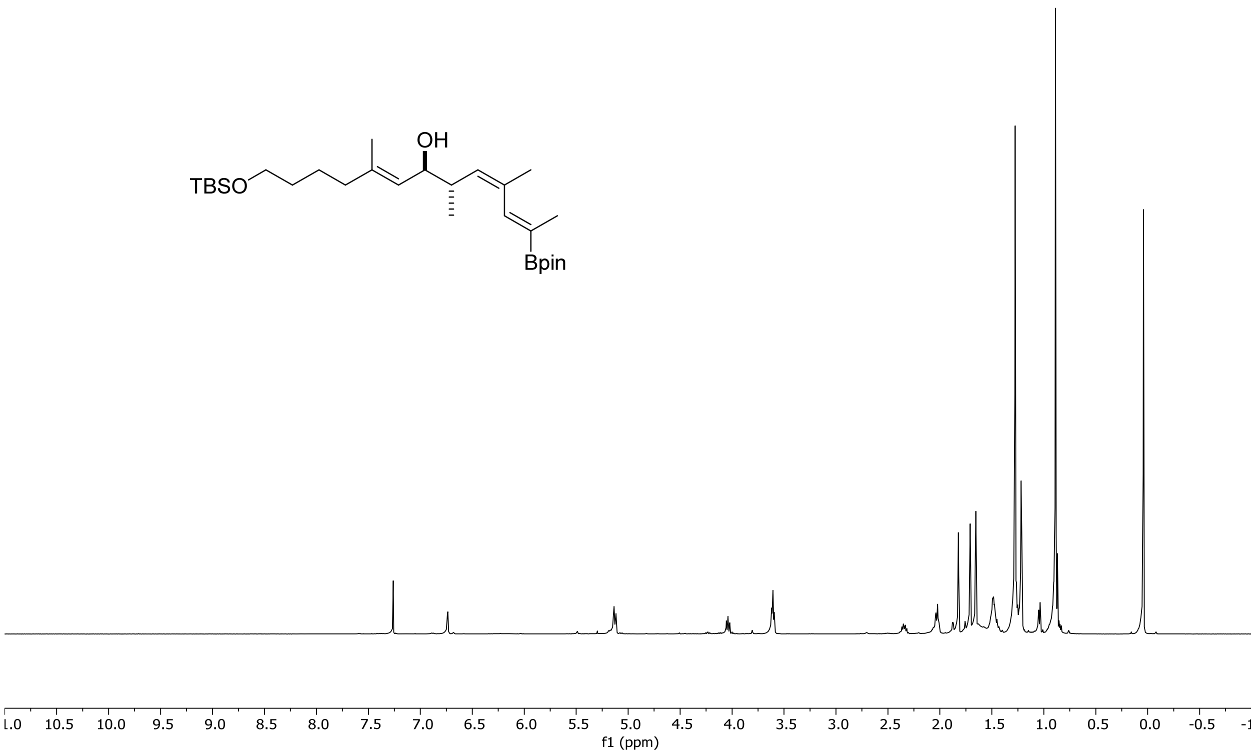
TBSO
OH
Bpin
f1 (ppm)

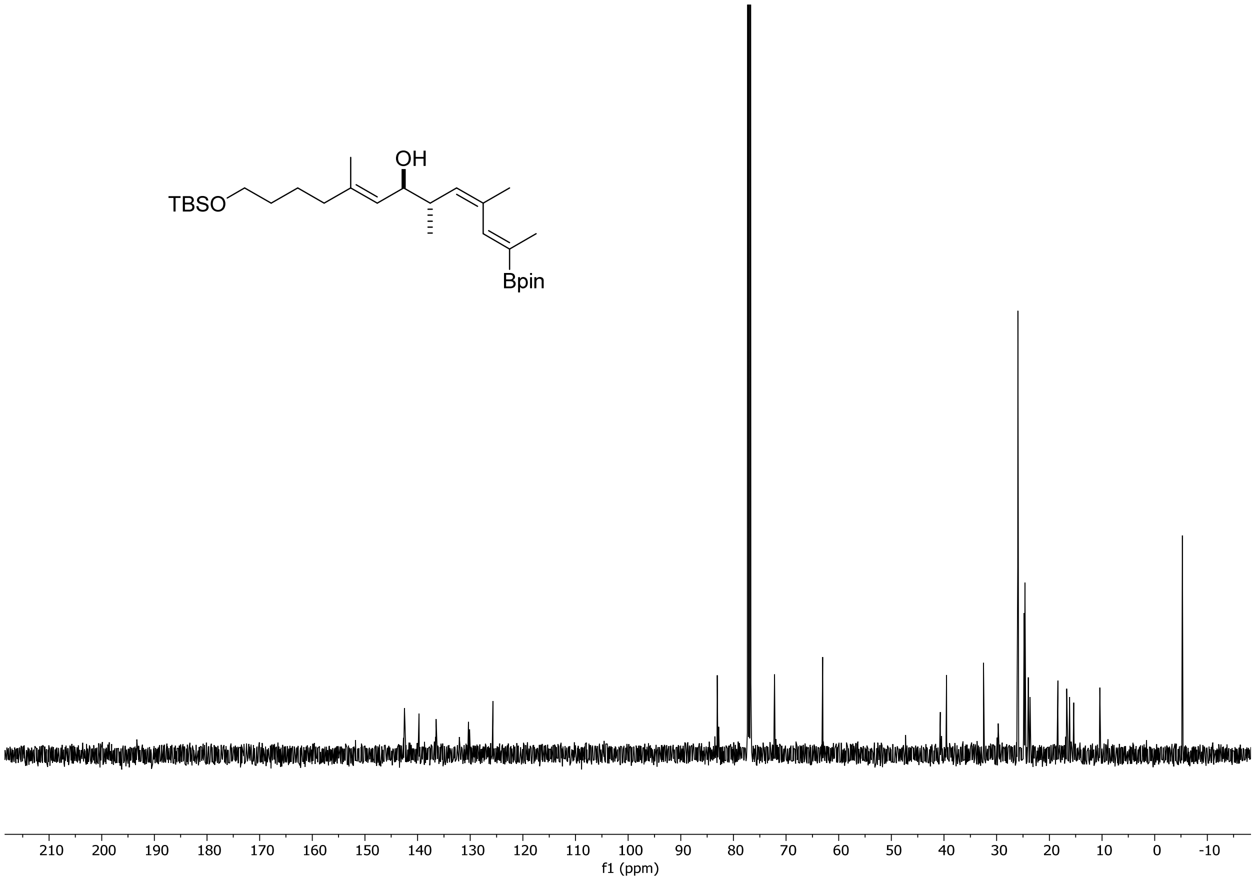
TBSO
OH
Bpin
f1 (ppm)

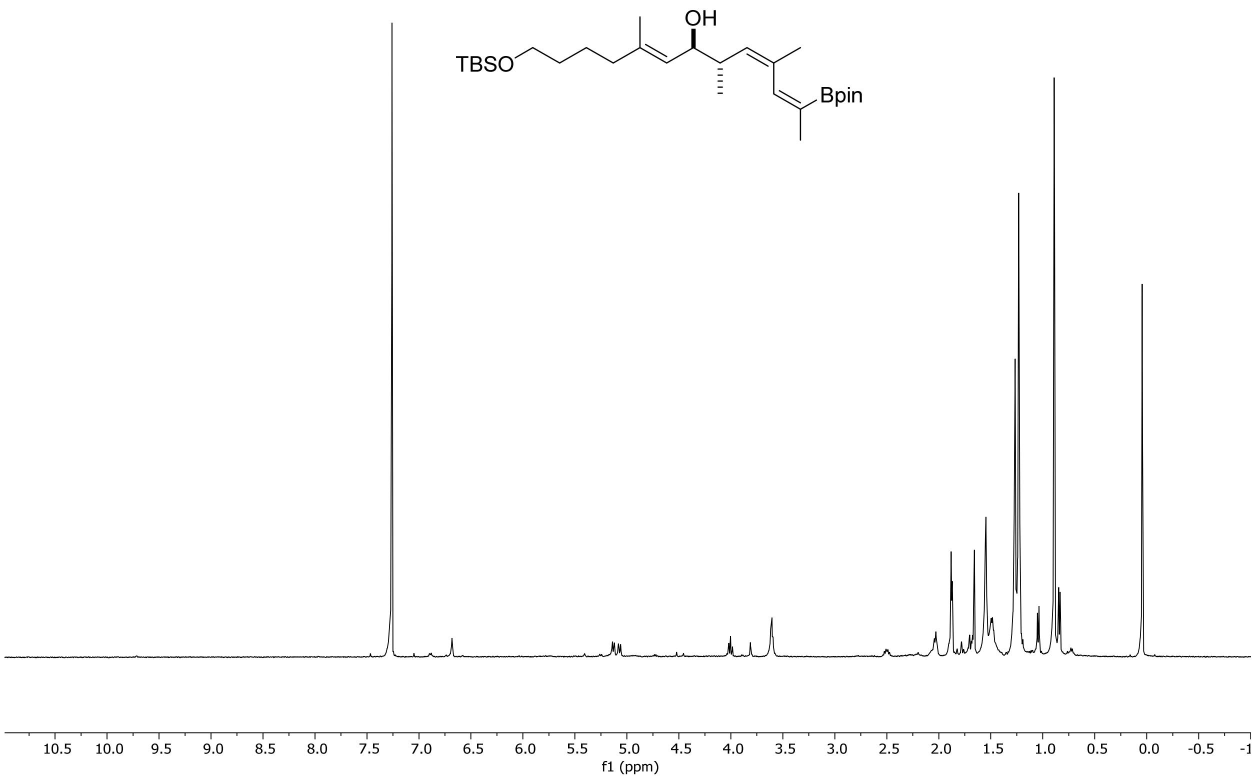
TBSO
OH
Bpin
10.5 10.0 9.5 9.0 8.5 8.0 7.5 7.0 6.5 6.0 5.5 5.0 4.5 4.0 3.5 3.0 2.5 2.0 1.5 1.0 0.5 0.0 -0.5
f1 (ppm)

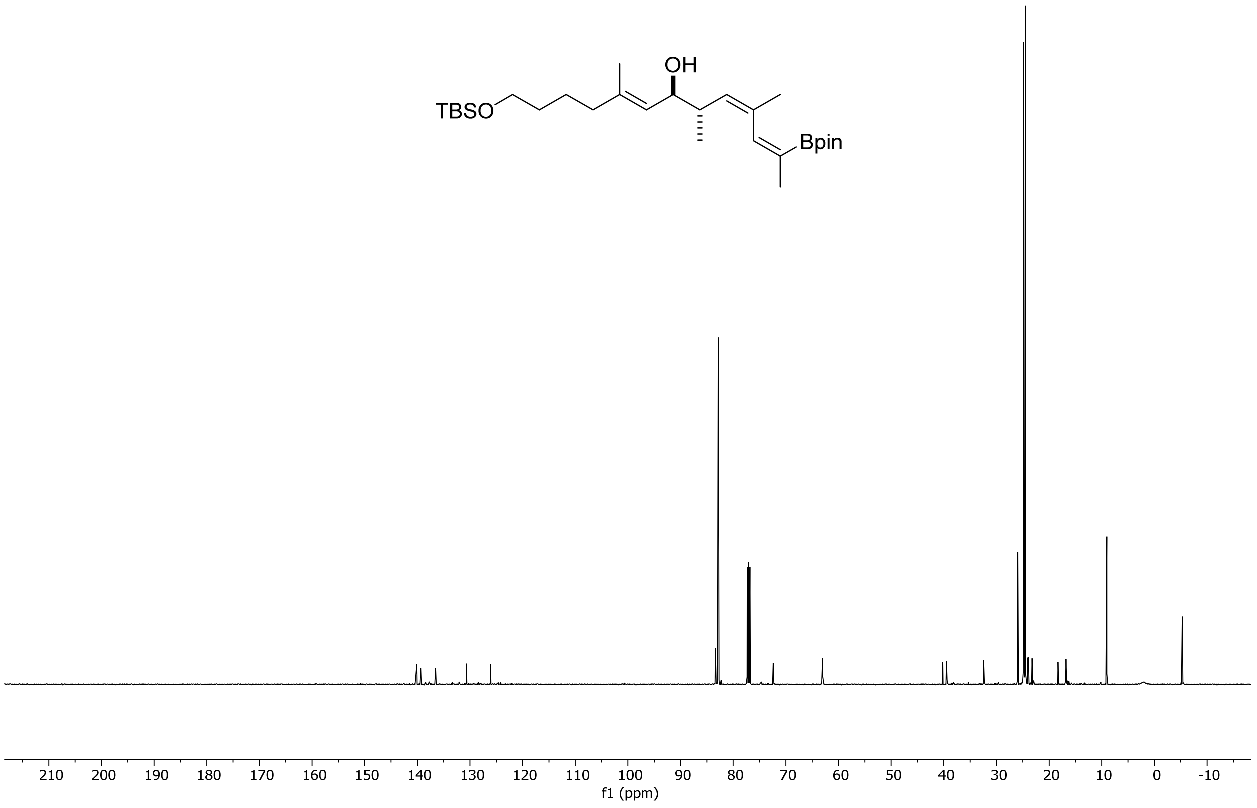
TBSO
OH
Bpin
210 200 190 180 170 160 150 140 130 120 110 100 90 80 70 60 50 40 30 20 10 0 -10
f1 (ppm)

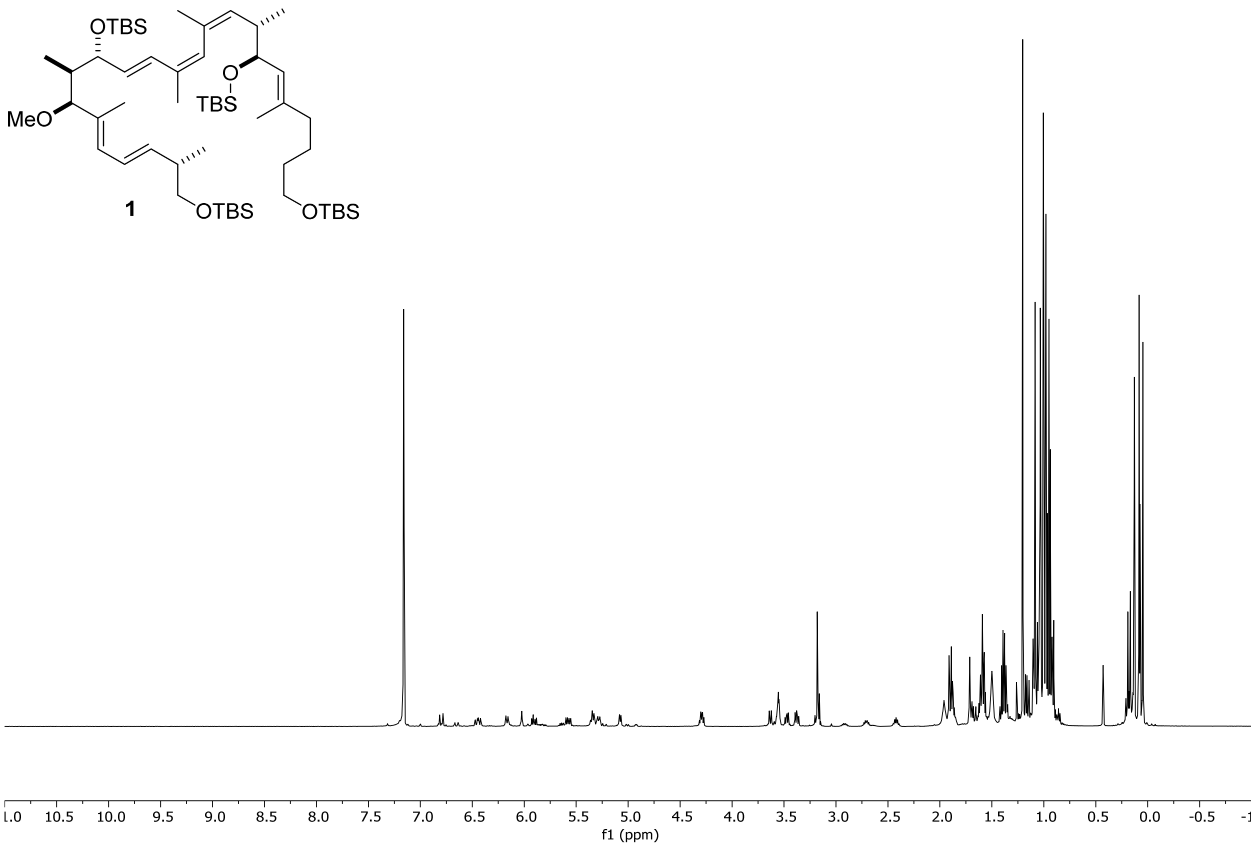
OTBS
O
TBS
MeO
1
OTBS
OTBS
[illegible] 10.5 10.0 9.5 9.0 8.5 8.0 7.5 7.0 6.5 6.0 5.5 5.0 4.5 4.0 3.5 3.0 2.5 2.0 1.5 1.0 0.5 0.0 -0.5 [illegible]
f1 (ppm)

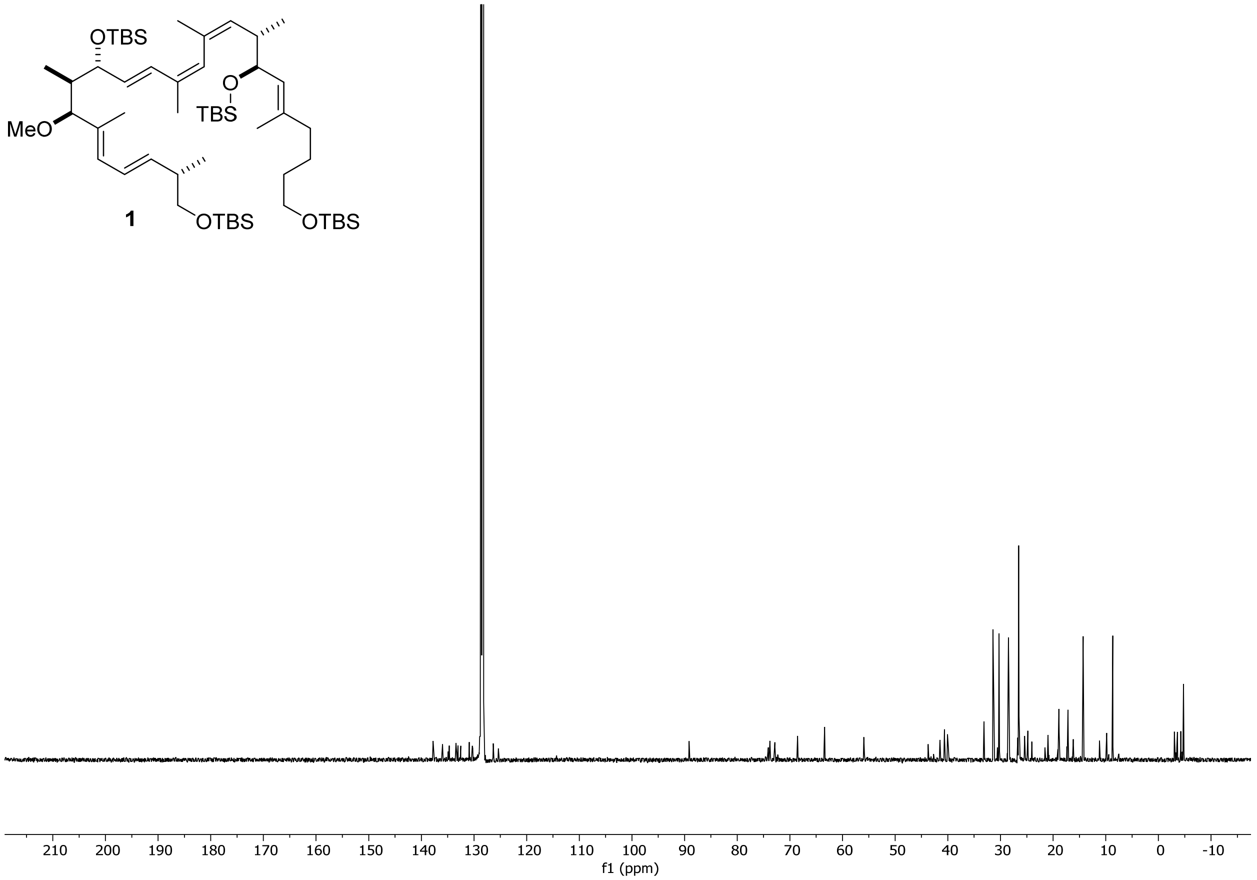
OTBS
O
TBS
MeO
1
OTBS
OTBS
210 200 190 180 170 160 150 140 130 120 110 100 90 80 70 60 50 40 30 20 10 0 -10
f1 (ppm)

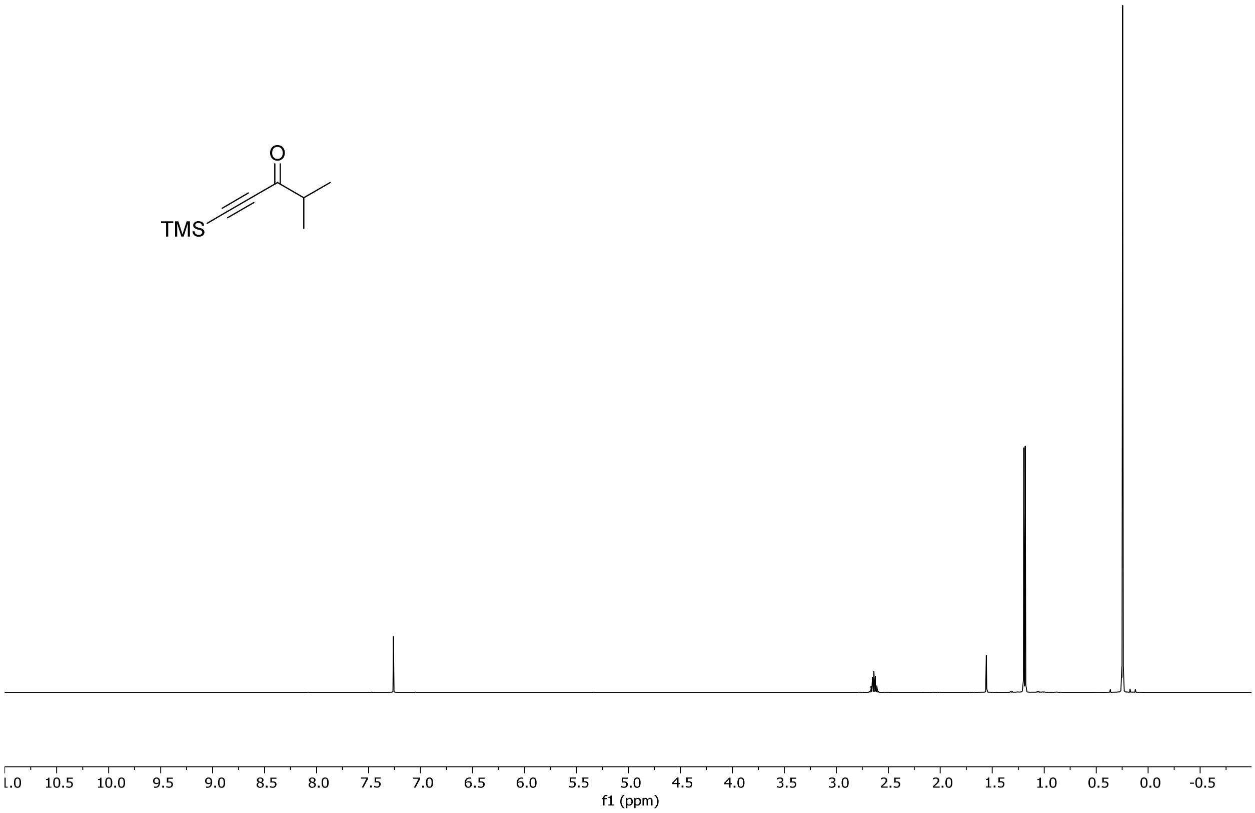
O
TMS
1.0 10.5 10.0 9.5 9.0 8.5 8.0 7.5 7.0 6.5 6.0 5.5 5.0 4.5 4.0 3.5 3.0 2.5 2.0 1.5 1.0 0.5 0.0 -0.5
f1 (ppm)

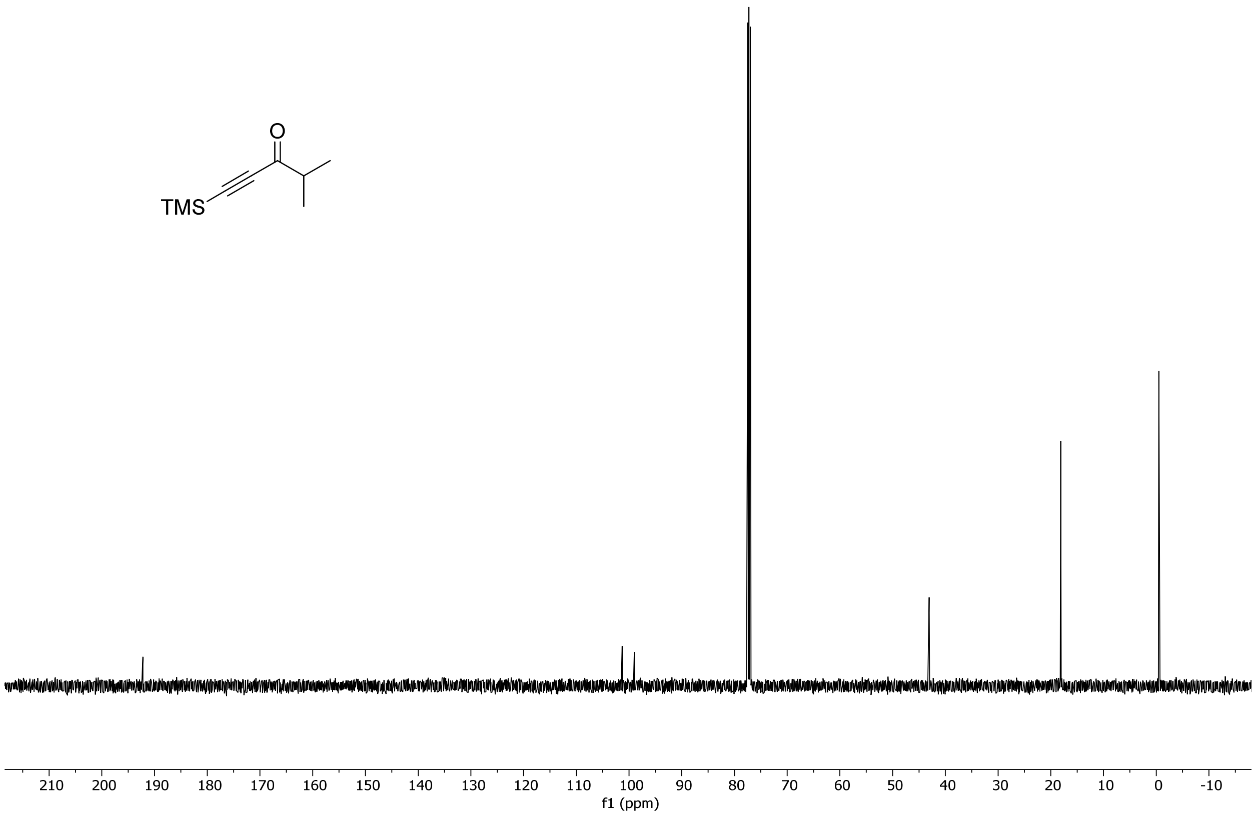
O
TMS
210 200 190 180 170 160 150 140 130 120 110 100 90 80 70 60 50 40 30 20 10 0 -10
f1 (ppm)

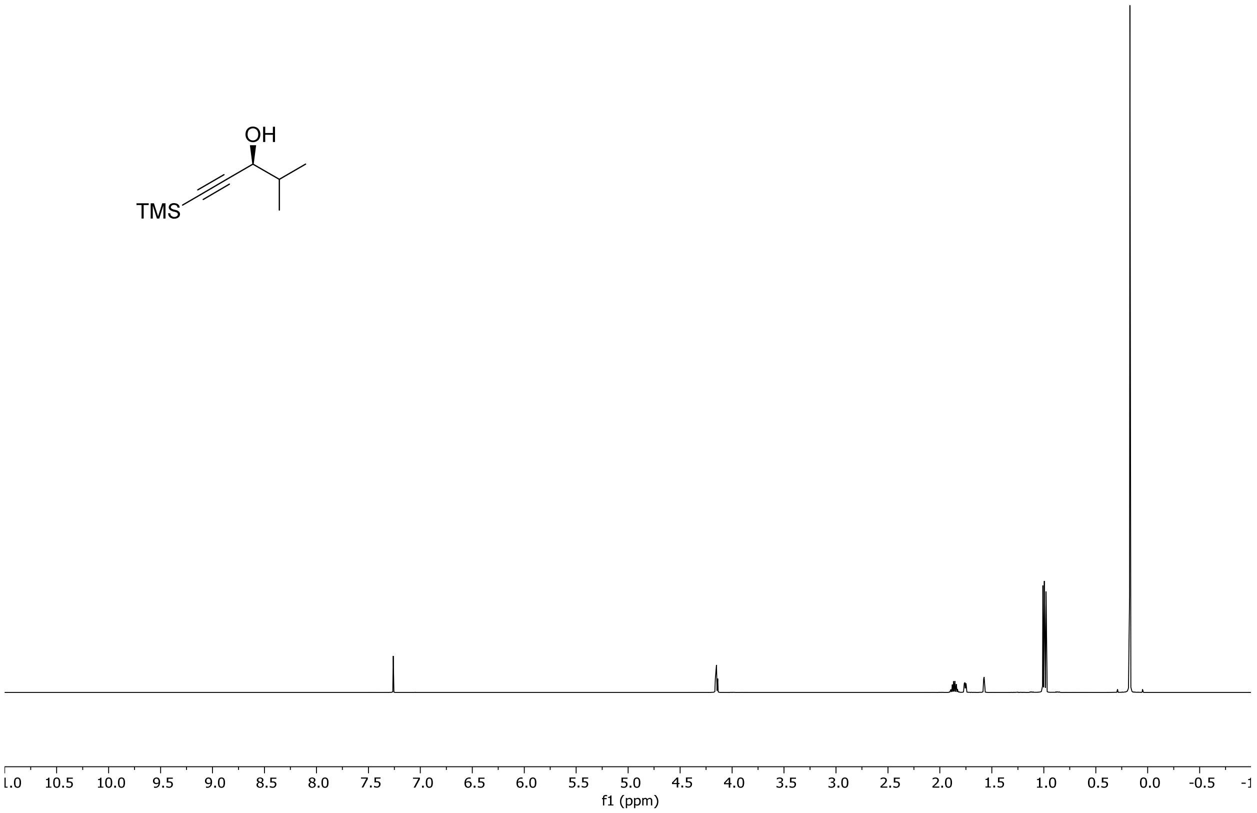
OH
TMS
f1 (ppm)

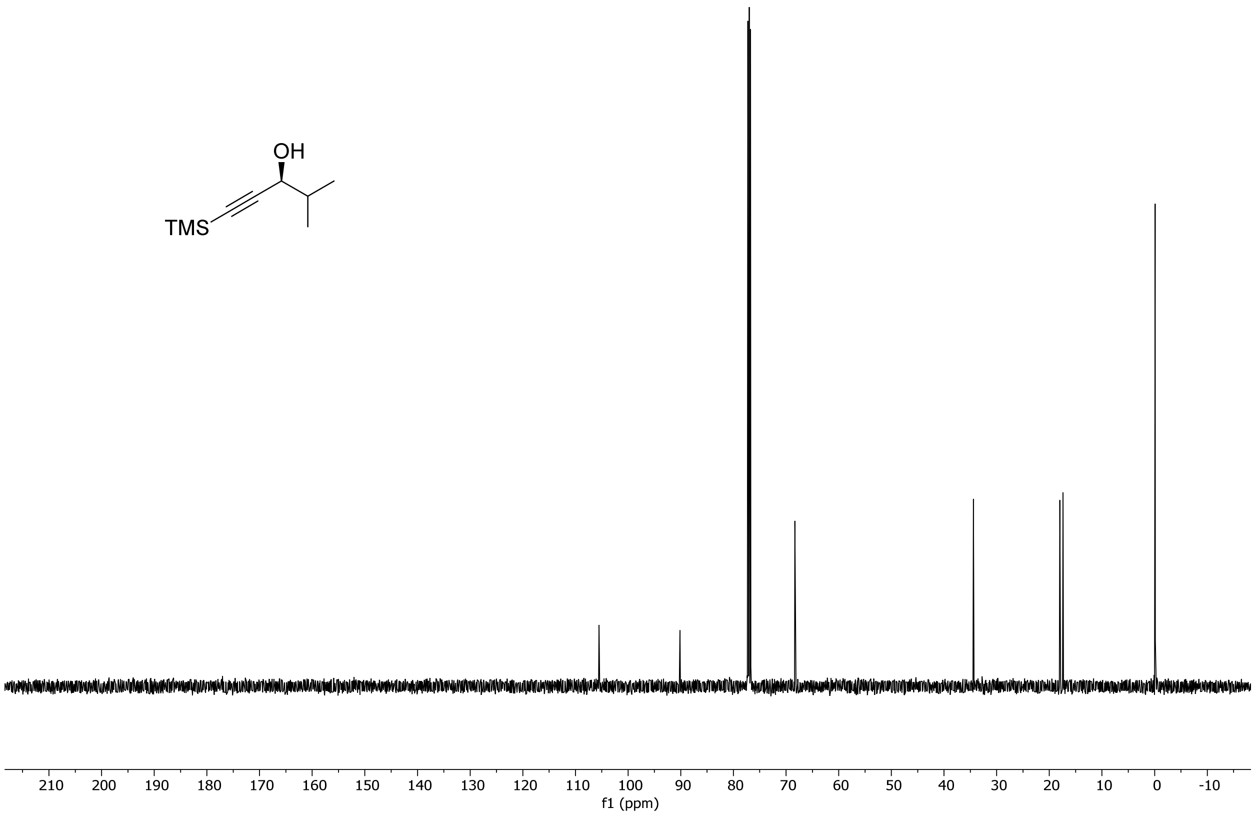
OH
TMS
f1 (ppm)

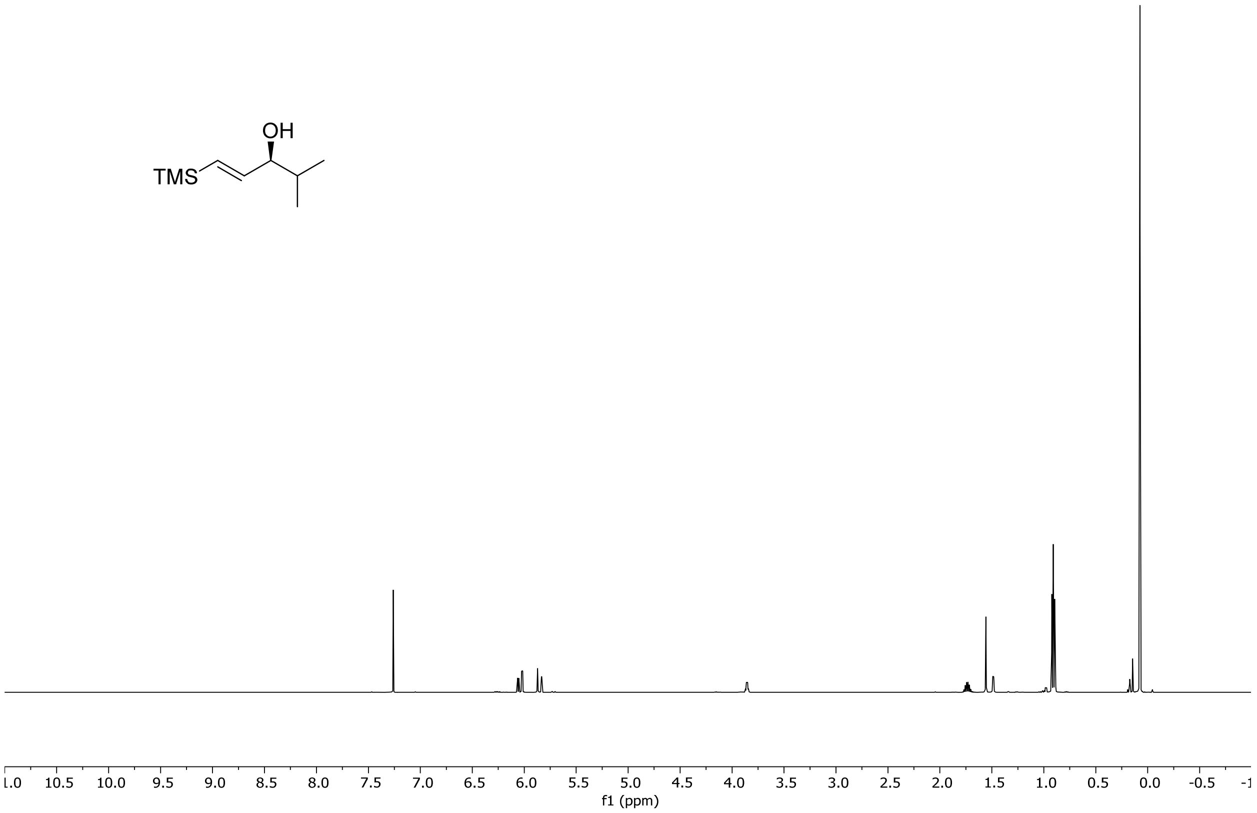
OH
TMS
f1 (ppm)

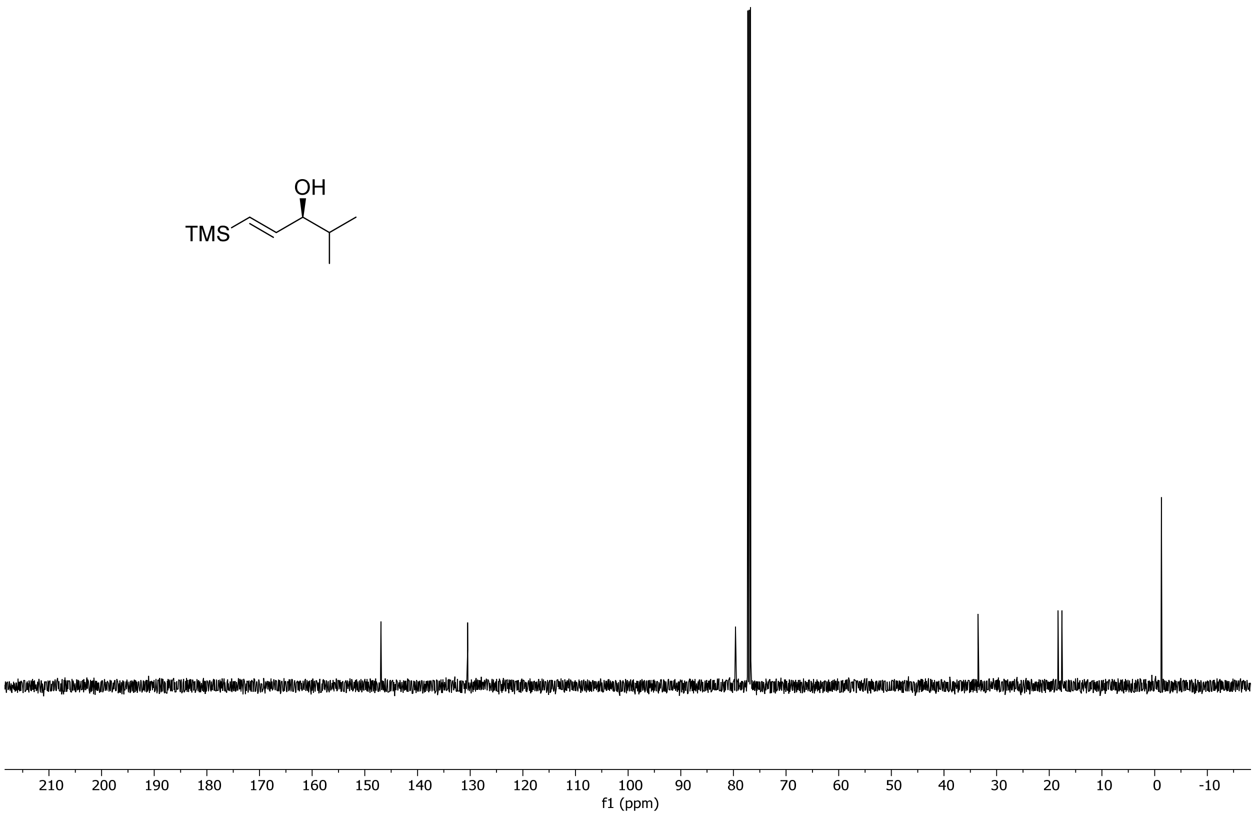
OH
TMS
f1 (ppm)

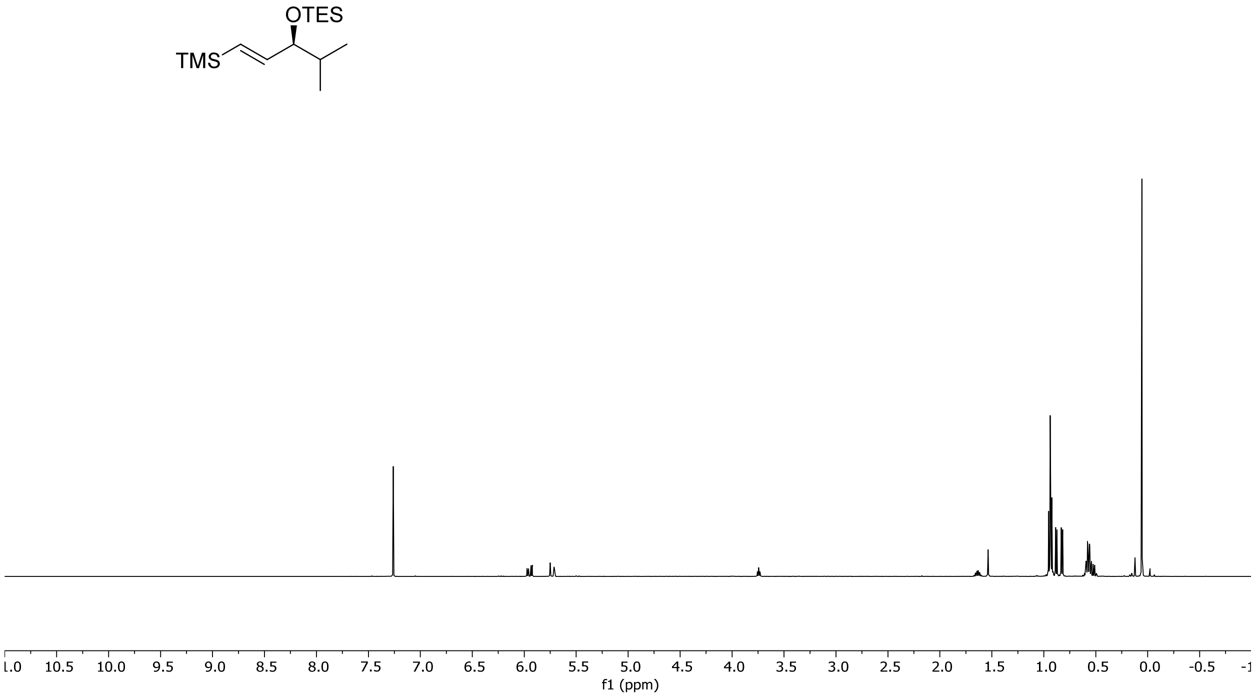
OTES
TMS
f1 (ppm)

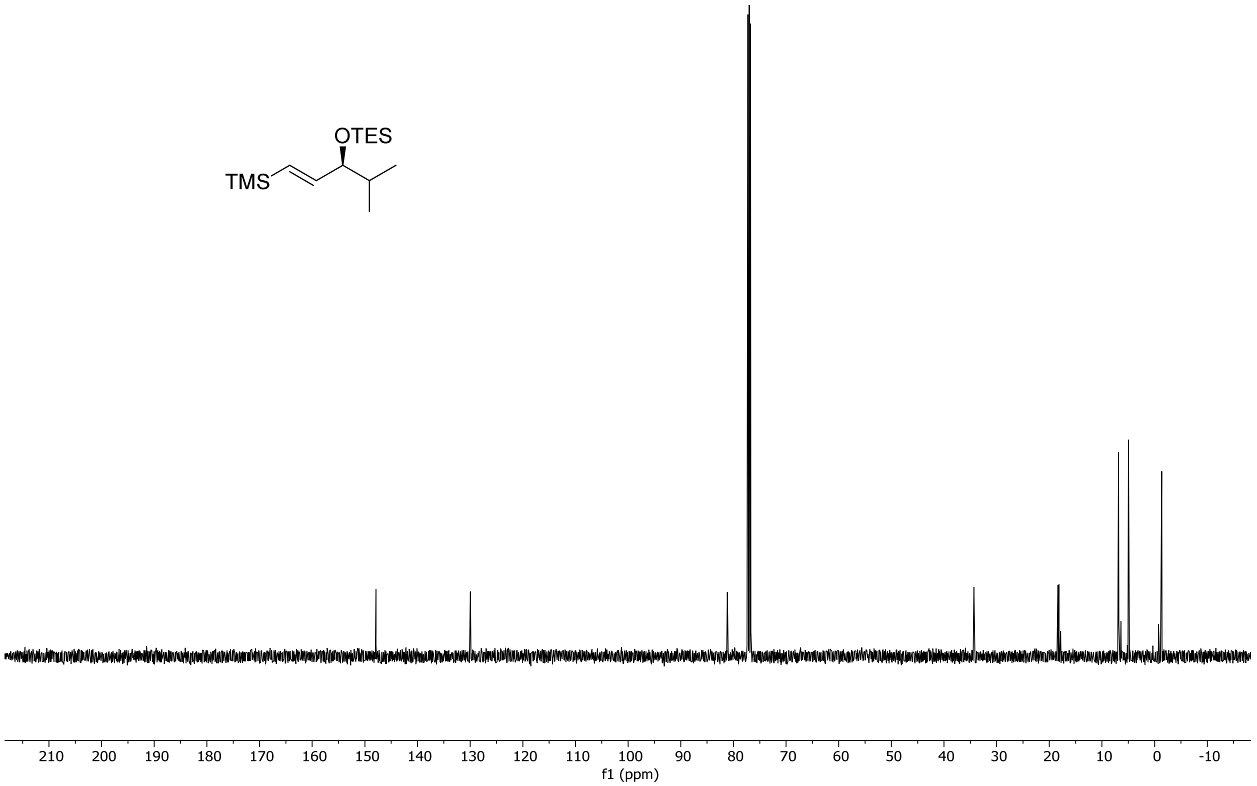
OTES
TMS
f1 (ppm)

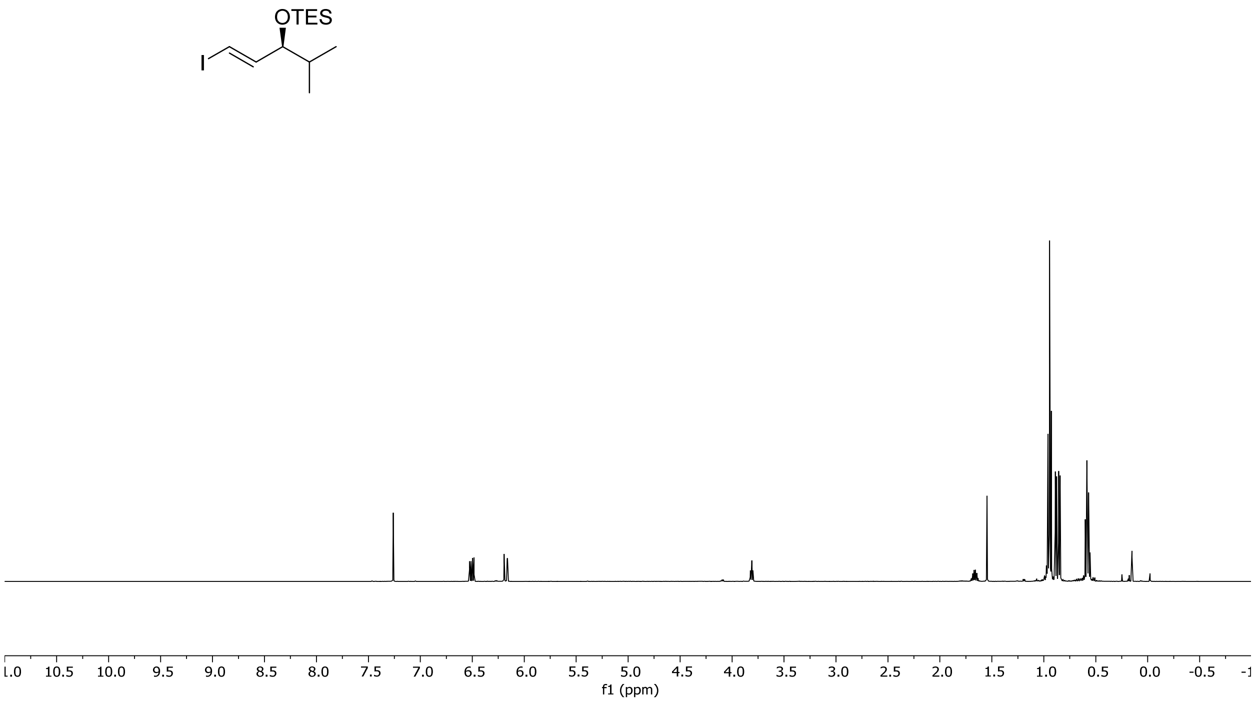
OTES
I
f1 (ppm)

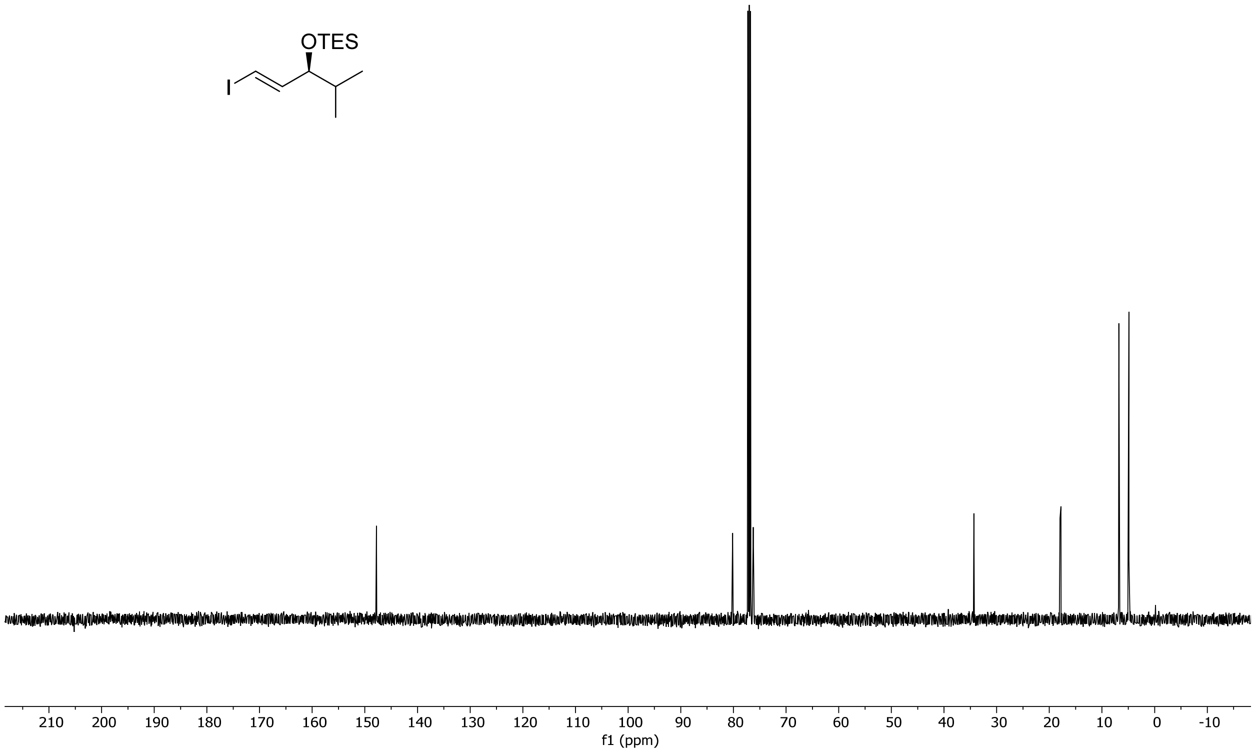
OTES
I
f1 (ppm)

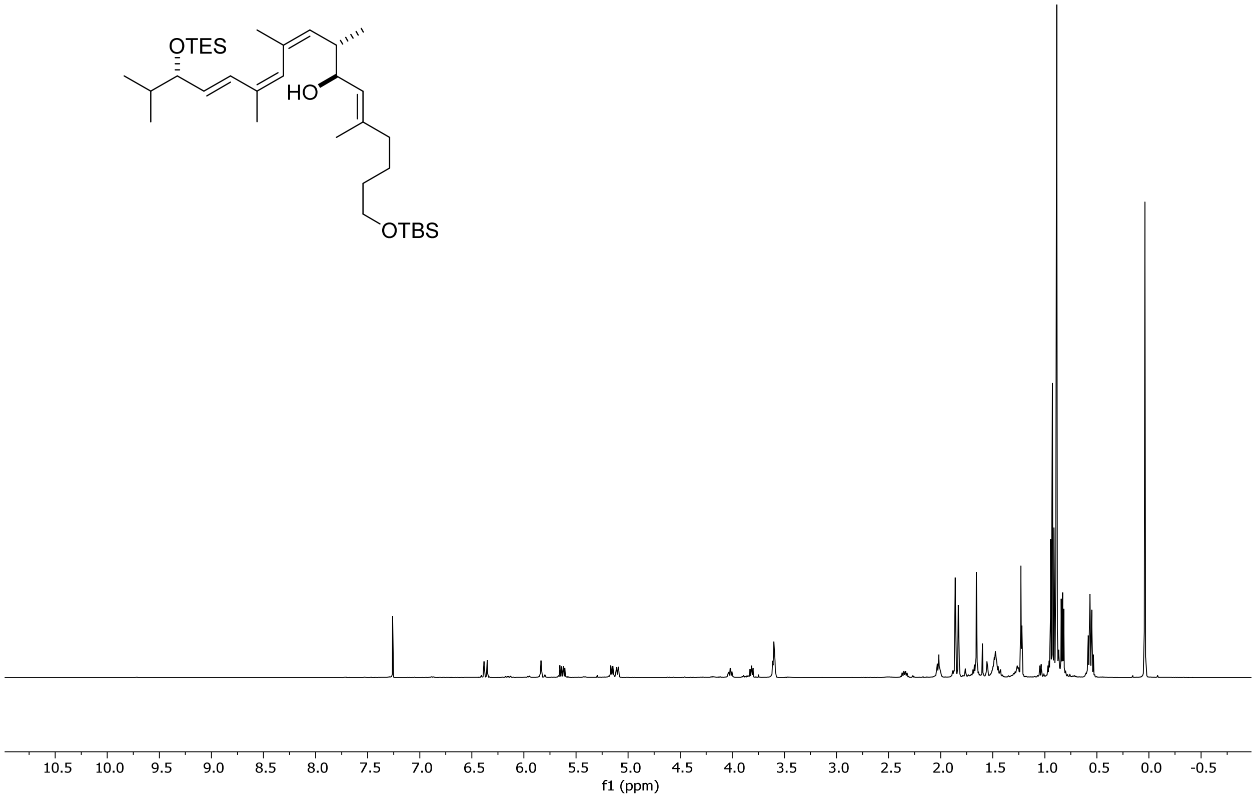
OTES
HO
OTBS
10.5 10.0 9.5 9.0 8.5 8.0 7.5 7.0 6.5 6.0 5.5 5.0 4.5 4.0 3.5 3.0 2.5 2.0 1.5 1.0 0.5 0.0 -0.5
f1 (ppm)

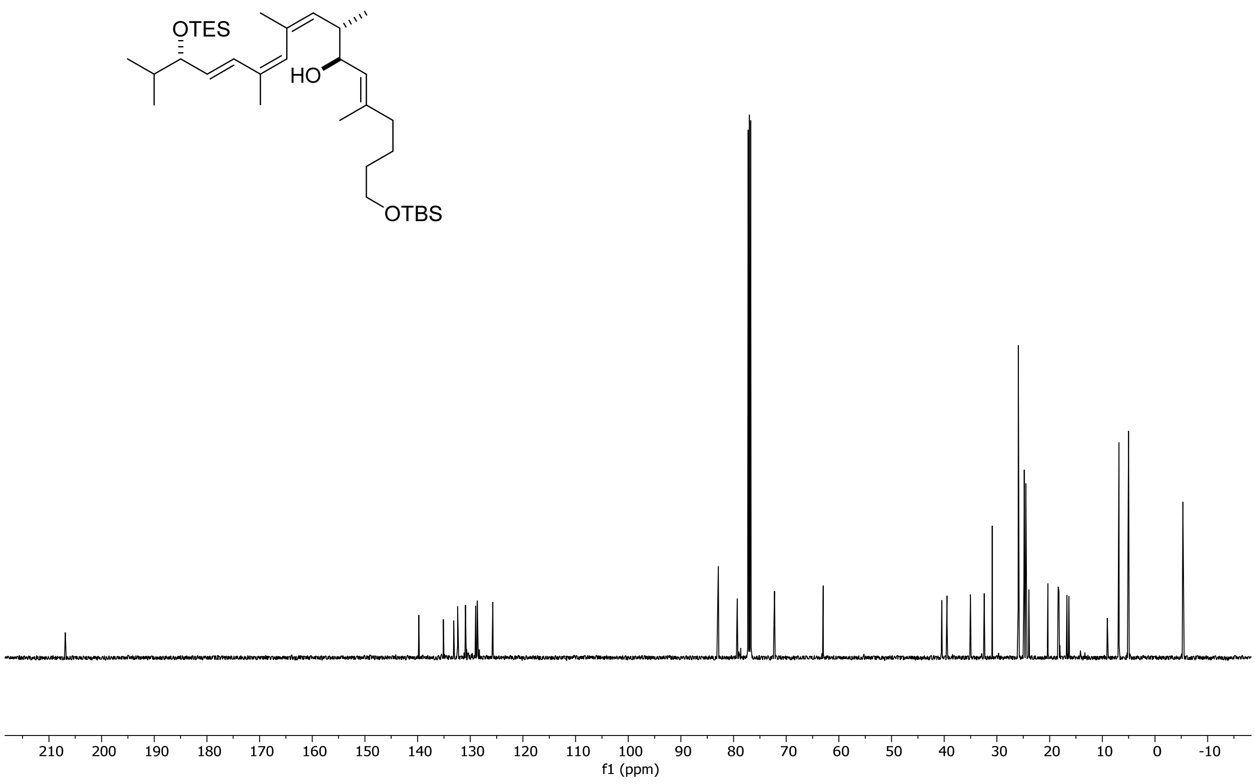
OTES
HO
OTBS
210 200 190 180 170 160 150 140 130 120 110 100 90 80 70 60 50 40 30 20 10 0 -10
f1 (ppm)

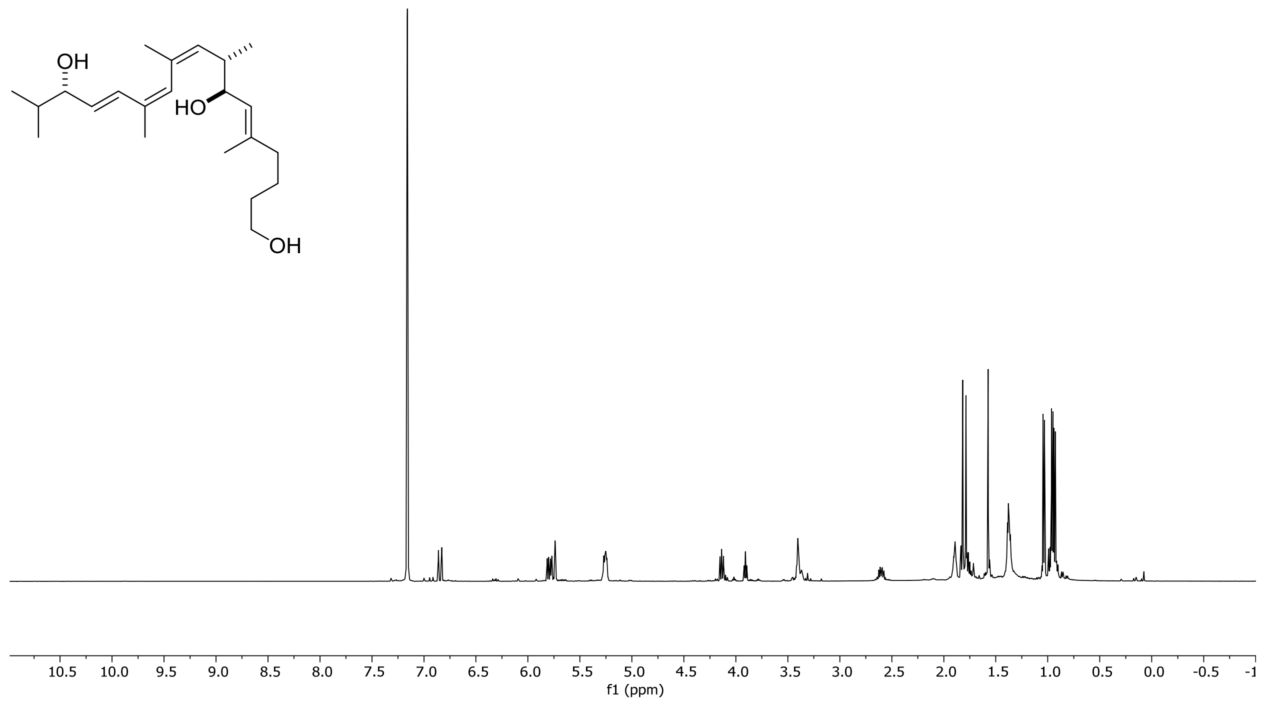
OH
HO
OH
10.5 10.0 9.5 9.0 8.5 8.0 7.5 7.0 6.5 6.0 5.5 5.0 4.5 4.0 3.5 3.0 2.5 2.0 1.5 1.0 0.5 0.0 -0.5 -1
f1 (ppm)

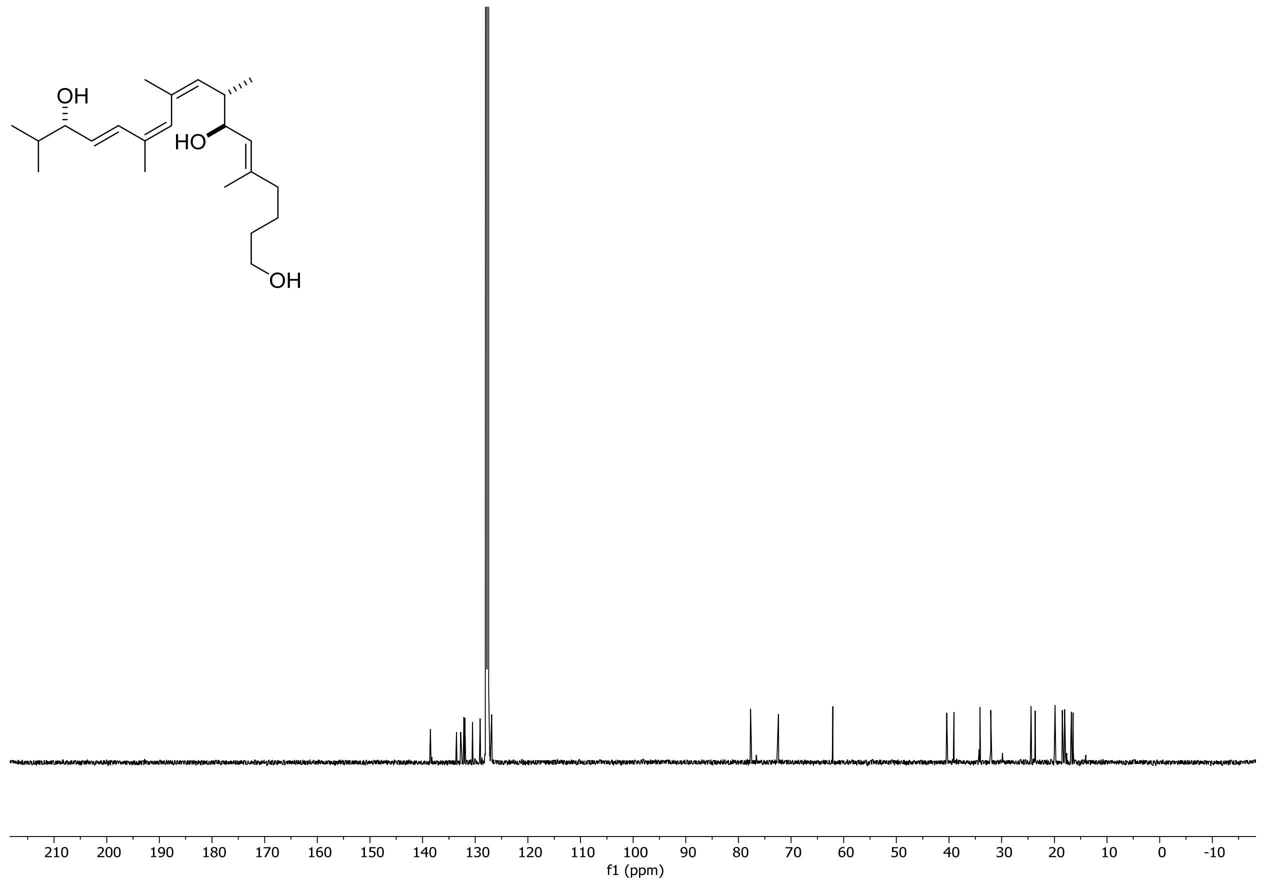
OH
HO
OH
210 200 190 180 170 160 150 140 130 120 110 100 90 80 70 60 50 40 30 20 10 0 -10
f1 (ppm)

Figure S1. NOESY Spectra of *E*- and *Z*-**15** showing correlations for geometry assignment.

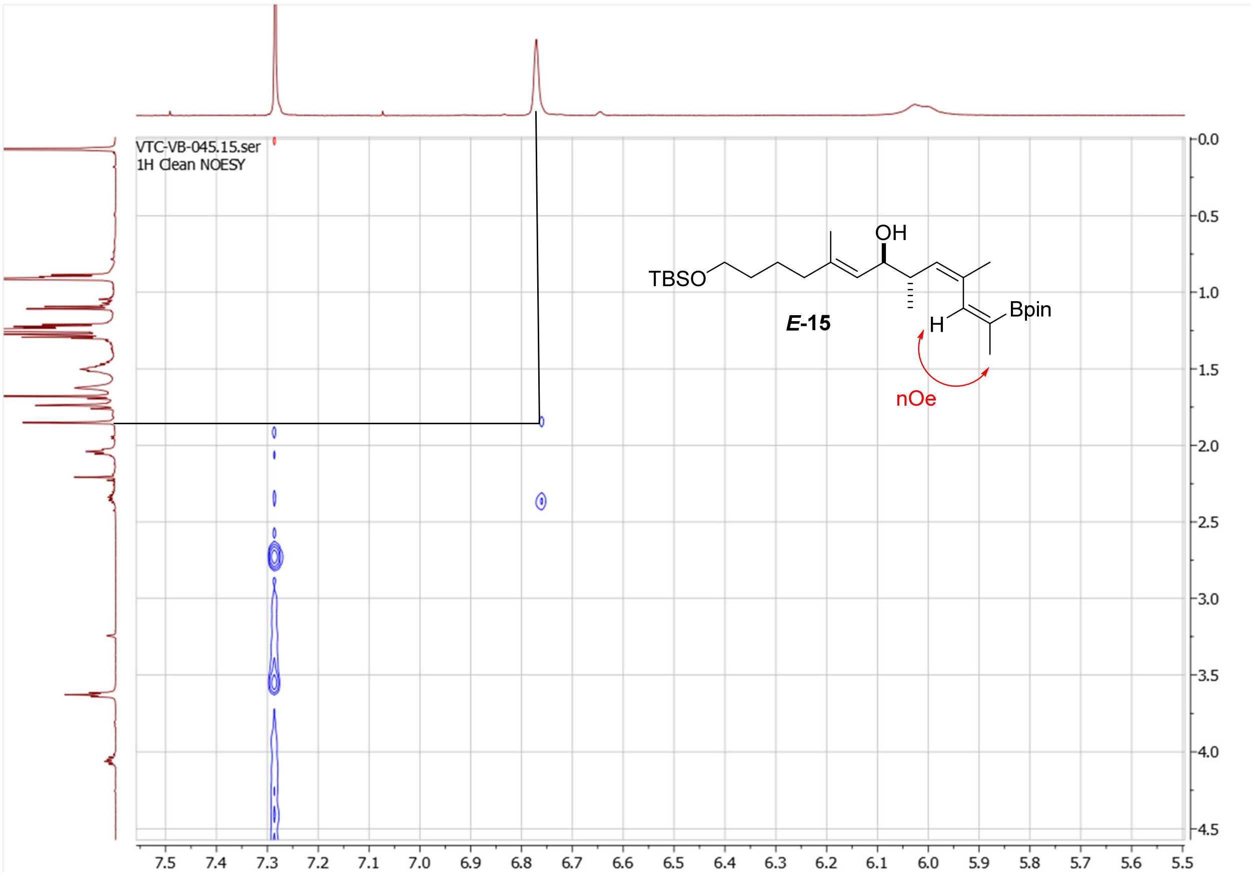

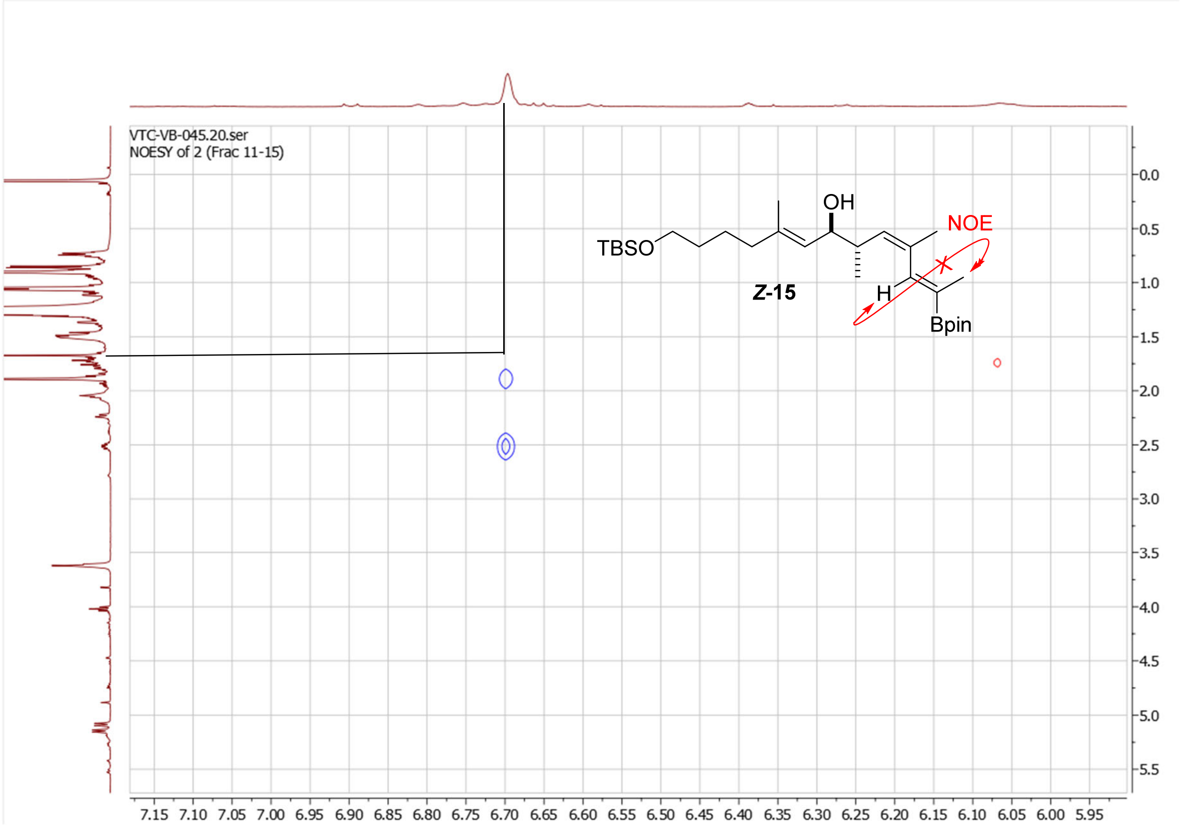

VATPase Assay

A bioassay was performed to test inhibition of V-ATPases in *Arabidopsis thaliana*. Dark grown *Arabidopsis* seedlings undergo etiolation, a process in which the hypocotyl elongates at a rapid rate. This process, which increases the likelihood that embryotic leaves will reach light, relies on increased cell turgor and subsequent cell elongation. V-ATPases create this change in turgor through active transport of protons into the vacuole, thus lowering water potential. Wild-type *Arabidopsis thaliana* of the ecotype Colombia were vernalized (4˚C dark treatment for one week after harvesting) then planted on 1% agar plates pH 5.7 containing .1x Murasinghe and Skoog Media. Compounds **1** or **20** as solutions in DCM were administered by adding to the media to the total concentrations indicated (Table S1). Additionally, control plants were grown in the absence of compounds **1** or **20** in equivalent amounts of DCM. All plants were placed in a 4˚C dark room for two days before being exposed to light for 24 hours to promote germination followed by seven days in aluminum foil to promote etiolation. Hypocotyls were photographed and measured using ImageJ.

Table S1. VATPase assay data.

Compound **1**:

Concentration (μmol)	Avg. length	SD^{A}	n	%inhibition^{B}
control	1.767289	0.245216	38	
0.125	1.555293	0.405454	41	11.99559
0.25	1.664214	0.284594	42	5.832388
0.5	1.686389	0.279155	36	4.577665
1	1.695475	0.289426	40	4.063538
2	1.624868	0.261123	38	8.058728
10	1.303868	0.283156	38	26.22214
20	0.971487	0.29061	39	45.02954

Compound **20**:

Concentration (μmol)	Avg. length	SD^{A}	n	% inhibition^{B}
Control	0.893328	0.316345	32	
10	0.860276	0.226829	29	3.699902
20	0.8634	0.254291	30	3.350183
50	0.670563	0.274142	32	24.9366
100	0.743937	0.314218	63	16.72304
200	0.618778	0.2569	63	30.73343
300	0.475813	0.236606	32	46.7371

Notes for Table: ^{A}Standard Deviation. ^{B}Calculated as (Avg. Length – Avg. $\text{Length}_{\text{control}}$)/Avg. $\text{Length}_{\text{control}}$

COX Inhibition Assay

To assess the ability of compound **20** to inhibit COX enzymes in a dose-dependent manner, compound **20** was serially diluted from 124 to 1.94 µM in DMSO and assayed with an ELISA-based COX (ovine/human) Inhibitor Screening Assay Kit (Cayman Chemical, Ann Arbor, MI) according to the manufacturer's instructions. This is a competition-based assay between prostaglandins (PGs) and a PG-acetylcholinesterase conjugate (PG-AChE) for a limiting amount of PG antiserum. Following the pre-incubation of both COX-1 (ovine) and COX-2 (human recombinant) with compound **20** for 10 minutes at 37°C, the reactions were mixed with arachidonic acid and subsequently quenched with Stannous Chloride. The ELISA-based detection of cyclooxygenase activity indicated no dose-dependent inhibition for either COX-1 or COX-2 by compound **20**. All of the internal controls for this assay were verified, including a standard curve of PG standards. Both COX-1 and COX-2 titrations were performed in duplicate, with duplicate reading in the microplate reader.

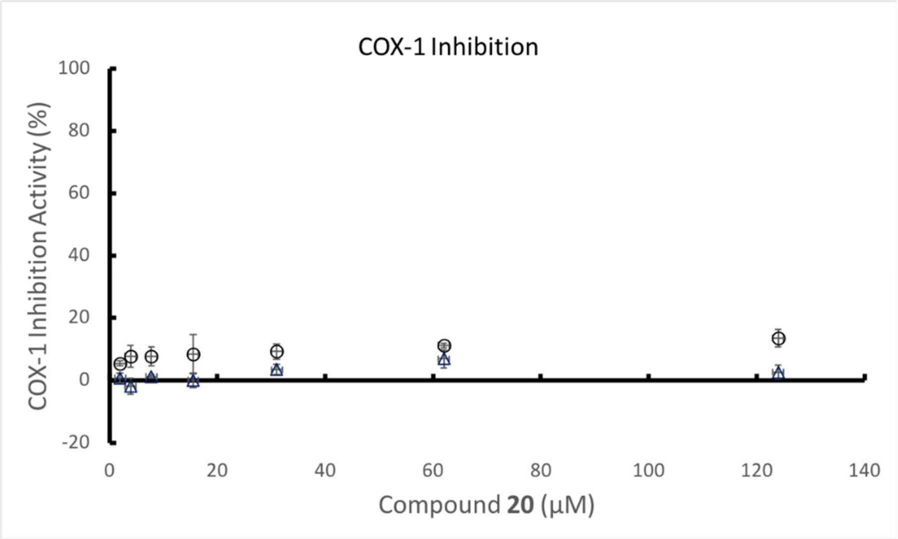

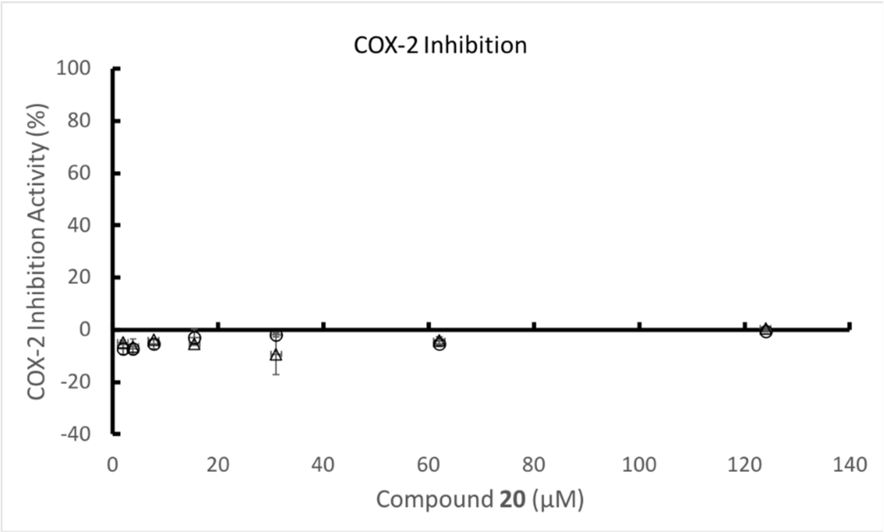

Supplement: RA-009-C9RA07050H-s001 [file RA-009-C9RA07050H-s001.pdf]
